# Supplementary material for: Genomic and socioeconomic drivers of antimicrobial resistance forecast to 2050
Source: Cell Genom. 2026 Jun 3;6(7):101273. doi: 10.1016/j.xgen.2026.101273 (PMC13347946; doi:10.1016/j.xgen.2026.101273)
Supplement: Document S1. Figures S1–S34, Tables S4, S20, and S21, supplemental data, and — supplemental methods [file mmc1.pdf]

Cell Genomics, Volume *6*

Supplemental information

Genomic and socioeconomic drivers of antimicrobial resistance forecast to 2050

Michelle Baker, Alexandre Maciel-Guerra, Ruoqi Wang, Chengchang Luo, Yan Xu, Enzo Guerrero-Araya, Weihua Meng, Ge Wu, Komkiew Pinpimai, Peter Anthony Oyom, Nicola Senin, and Tania Dottorini

Supplemental Information for Genomic and socioeconomic drivers of antimicrobial resistance forecast to 2050

Baker *et al.*

Corresponding author email: tania.dottorini@kcl.ac.uk

This PDF file includes:

Figures S1 to S34
Tables S4, S20 and S21
Supplemental Data 1
Supplemental Methods 1

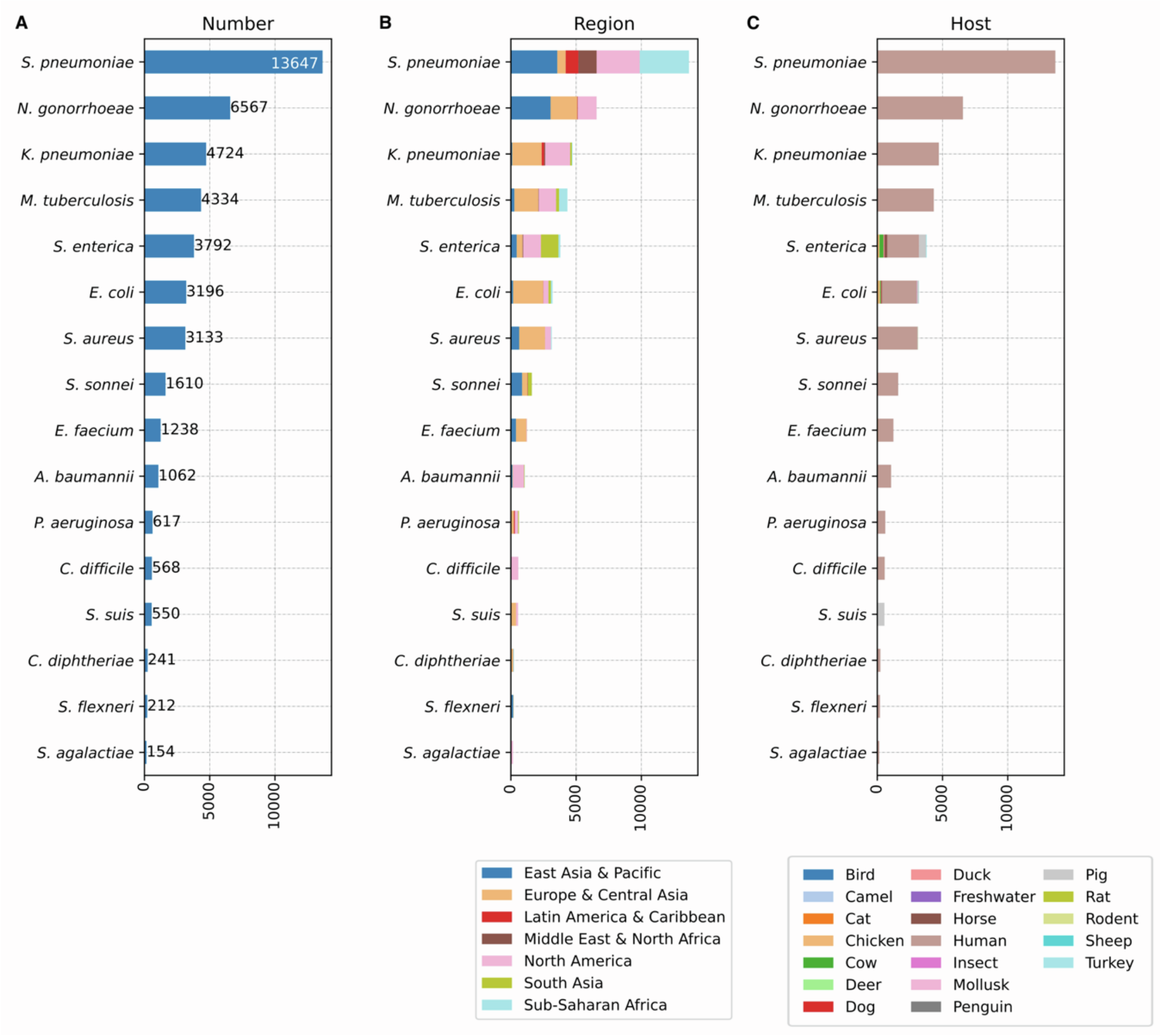

Figure S1. Bar plots showing distribution of isolates selected for analysis from BV-BRC database, related to Figure 1. Bacterial species were included if there were more than 100 high quality DNA sequences with accompanying resistance/susceptibility profiles in the database (see **STAR Methods**). (A) Number of isolates per bacterial species. (B) Region of origin for included isolates, bars coloured by World Bank regions. (C) Host of isolates, bars coloured by host.

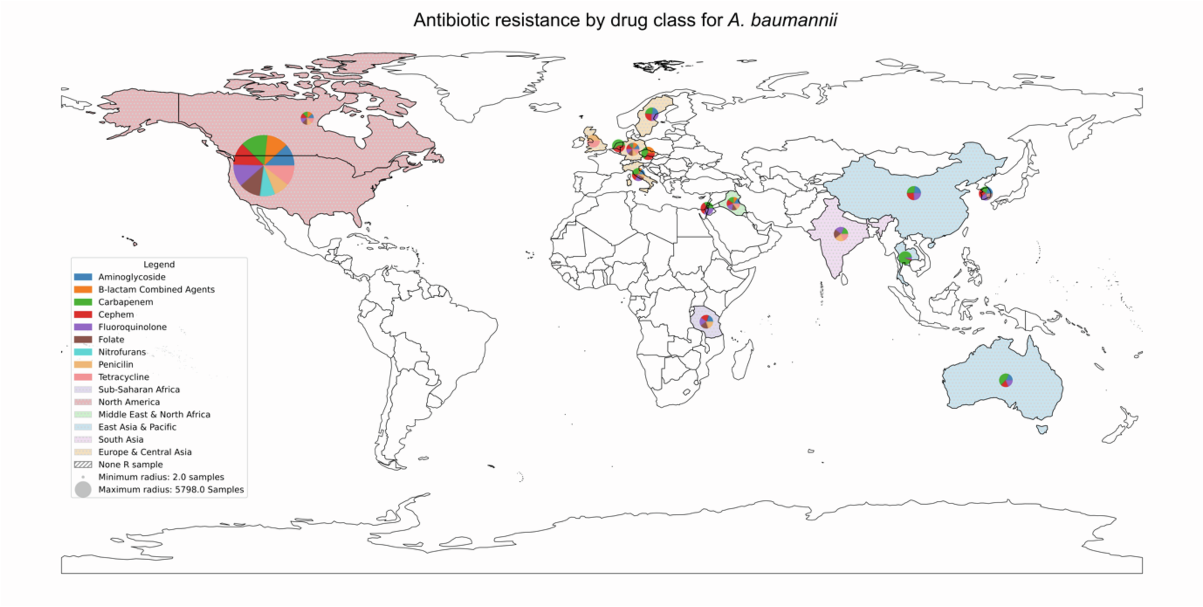

Figure S2. **Geographic distributions of 1,062 *A. baumannii* isolates with 12,074 associated resistance susceptibility phenotypic profiles obtained from the BV-BRC database[1] related to Figure 1.** The pie chart on each country shows the proportion of resistance profiles by class for each country, with the size of the chart indicating the overall number of genomes. The world map[2] has been divided according to the seven World Bank regions (East Asia and Pacific; Europe and Central Asia; Latin America and Caribbean; Middle East and North Africa; North America; South Asia; Sub-Saharan Africa).

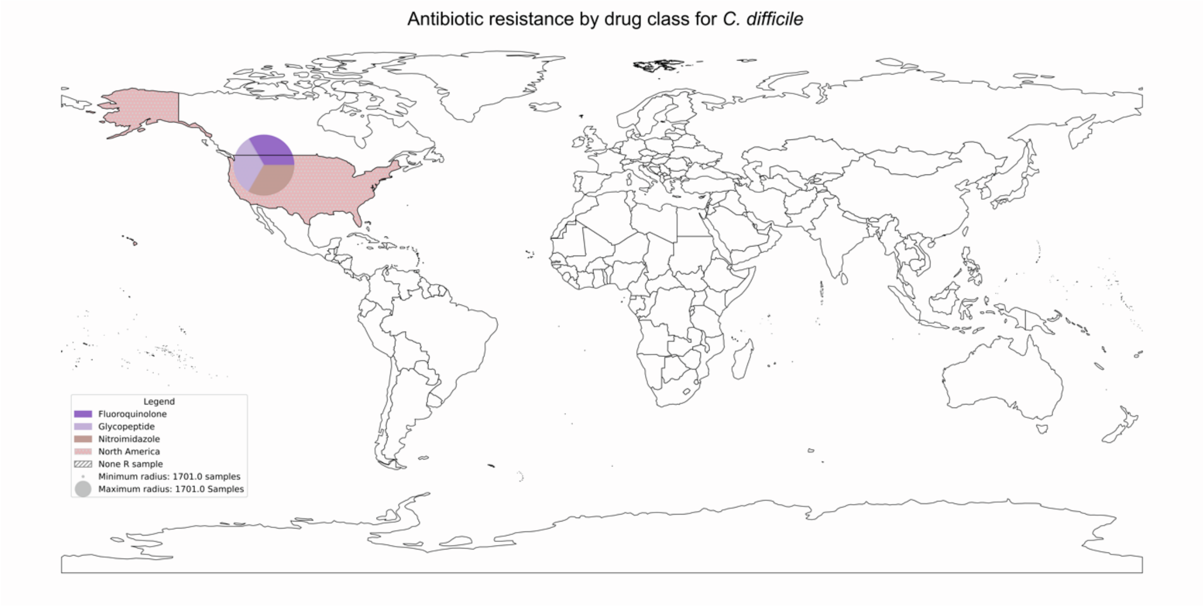

Figure S3. **Geographic distributions of 568 *C. difficile* isolates with 1,701 associated resistance susceptibility phenotypic profiles obtained from the BV-BRC database[1], related to Figure 1**. The pie chart on each country shows the proportion of resistance profiles by class for each country, with the size of the chart indicating the overall number of genomes. The world map[2] has been divided according to the seven World Bank regions (East Asia and Pacific; Europe and Central Asia; Latin America and Caribbean; Middle East and North Africa; North America; South Asia; Sub-Saharan Africa).

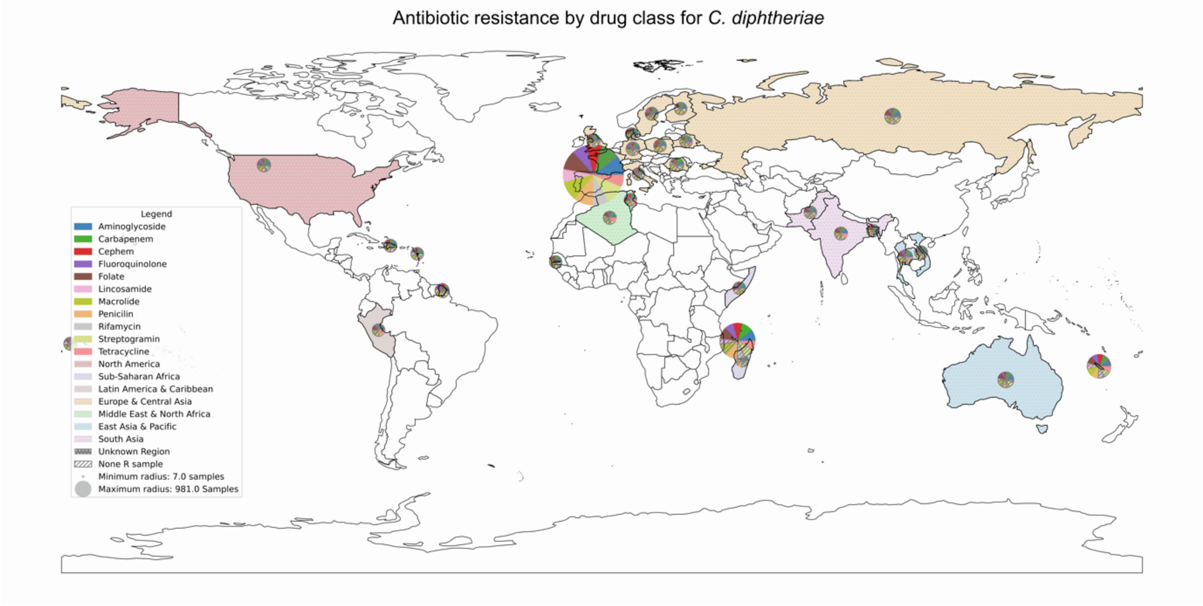

Figure S4. Geographic distributions of 241 *C. diphtheriae* isolates with 3,899 associated resistance susceptibility phenotypic profiles obtained from the BV-BRC database[1], related to Figure 1. The pie chart on each country shows the proportion of resistance profiles by class for each country, with the size of the chart indicating the overall number of genomes. The world map[2] has been divided according to the seven World Bank regions (East Asia and Pacific; Europe and Central Asia; Latin America and Caribbean; Middle East and North Africa; North America; South Asia; Sub-Saharan Africa).

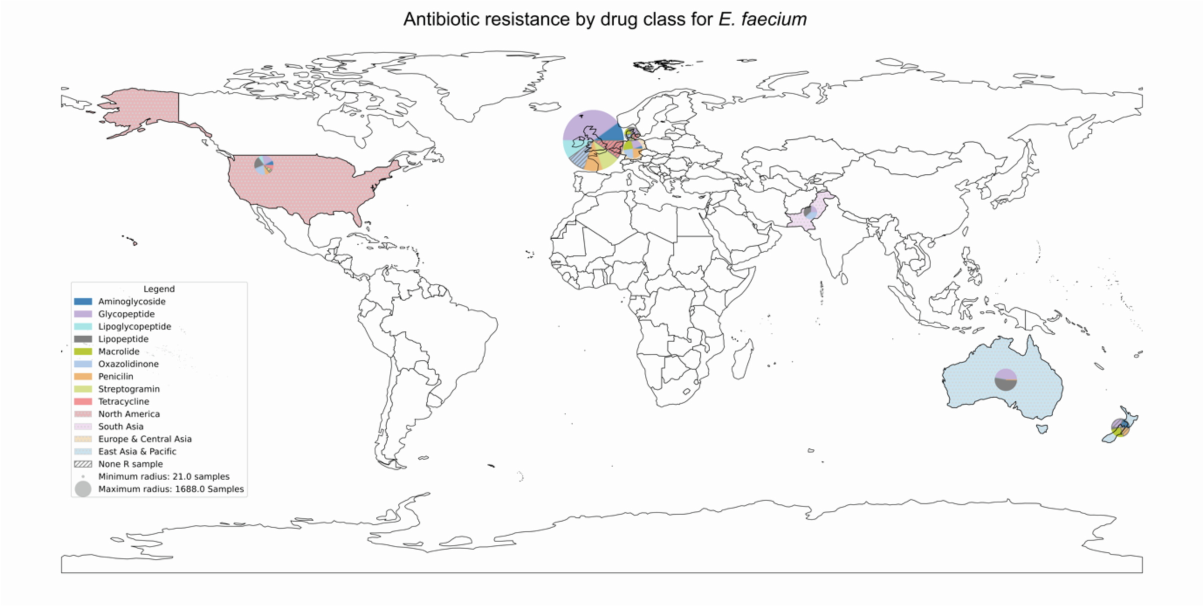

Figure S5. Geographic distributions of 1,238 *E. faecium* isolates with 2,960 associated resistance susceptibility phenotypic profiles obtained from the BV-BRC database[1], related to Figure 1. The pie chart on each country shows the proportion of resistance profiles by class for each country, with the size of the chart indicating the overall number of genomes. The world map[2] has been divided according to the seven World Bank regions (East Asia and Pacific; Europe and Central Asia; Latin America and Caribbean; Middle East and North Africa; North America; South Asia; Sub-Saharan Africa).

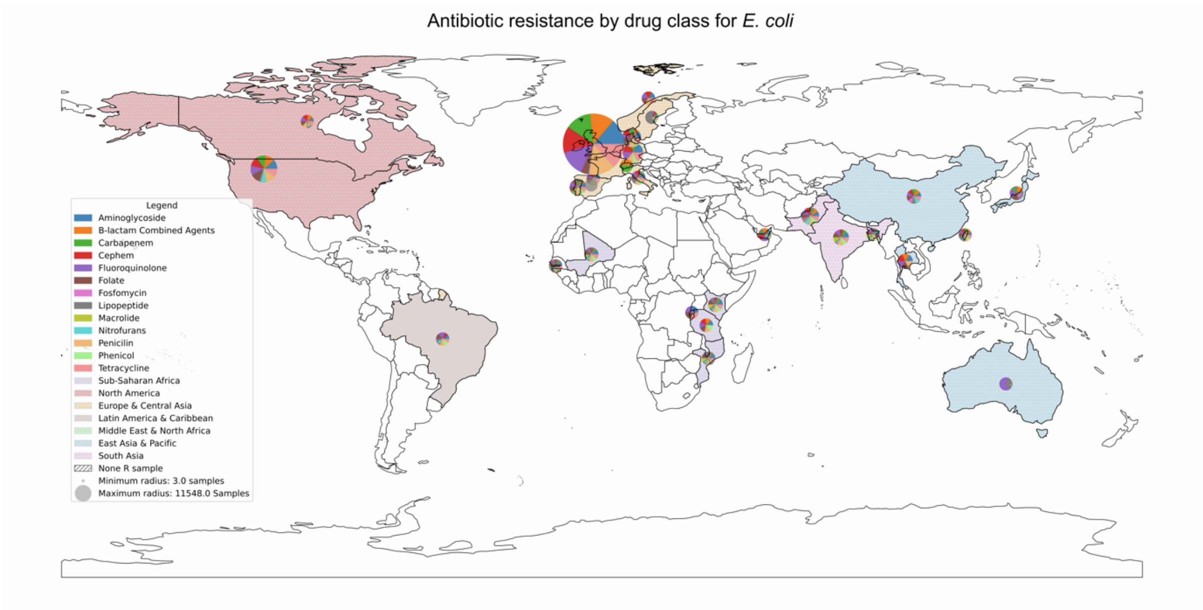

Figure S6. Geographic distributions of 3,196 *E. coli* isolates with 39,855 associated resistance susceptibility phenotypic profiles obtained from the BV-BRC database[1], related to Figure 1. The pie chart on each country shows the proportion of resistance profiles by class for each country, with the size of the chart indicating the overall number of genomes. The world map[2] has been divided according to the seven World Bank regions (East Asia and Pacific; Europe and Central Asia; Latin America and Caribbean; Middle East and North Africa; North America; South Asia; Sub-Saharan Africa).

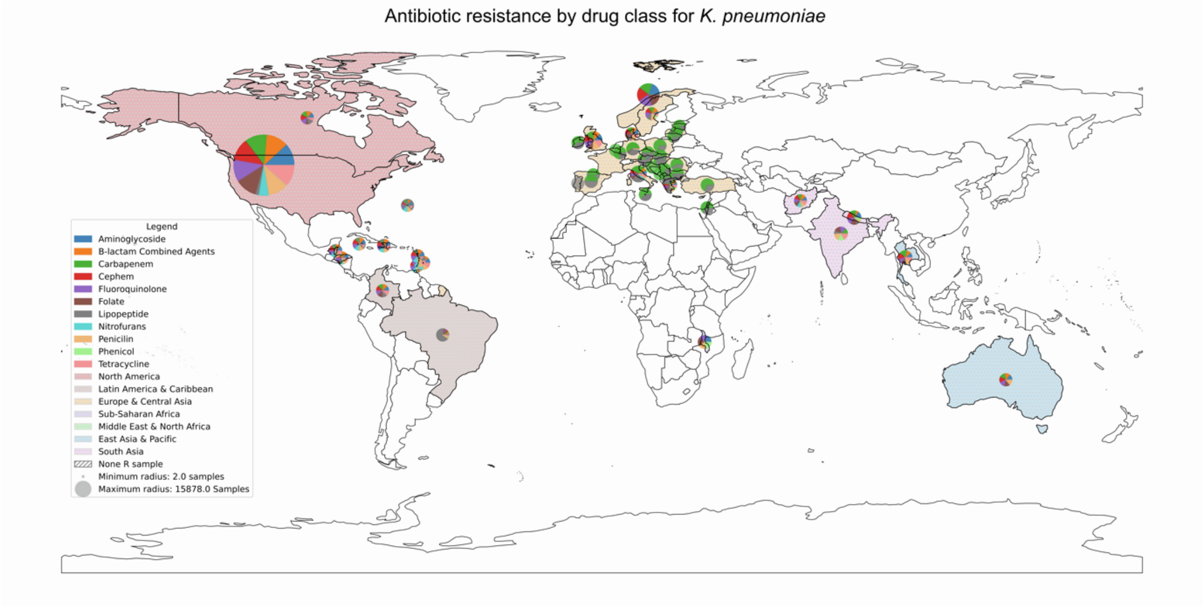

Figure S7. Geographic distributions of 4,724 *K. pneumoniae* isolates with 51,896 associated resistance susceptibility phenotypic profiles obtained from the BV-BRC database[1] **related to Figure 1.** The pie chart on each country shows the proportion of resistance profiles by class for each country, with the size of the chart indicating the overall number of genomes. The world map[2] has been divided according to the seven World Bank regions (East Asia and Pacific; Europe and Central Asia; Latin America and Caribbean; Middle East and North Africa; North America; South Asia; Sub-Saharan Africa).

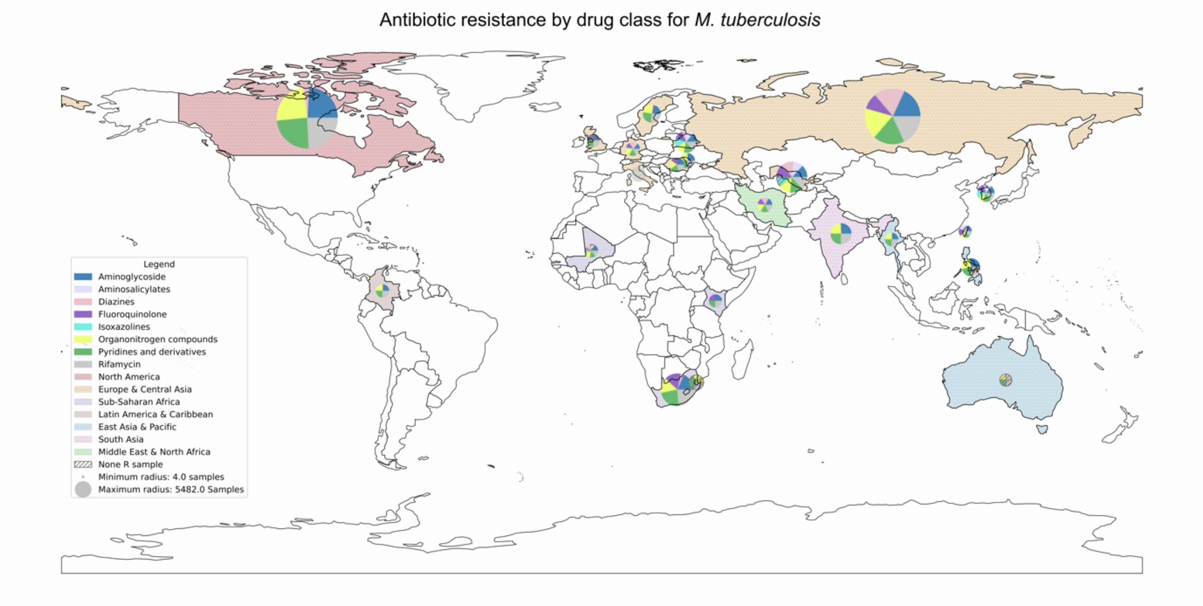

Figure S8. **Geographic distributions of 4,334 *M. tuberculosis* isolates with 24,710 associated resistance susceptibility phenotypic profiles obtained from the BV-BRC database[1], related to Figure 1**. The pie chart on each country shows the proportion of resistance profiles by class for each country, with the size of the chart indicating the overall number of genomes. The world map[2] has been divided according to the seven World Bank regions (East Asia and Pacific; Europe and Central Asia; Latin America and Caribbean; Middle East and North Africa; North America; South Asia; Sub-Saharan Africa).

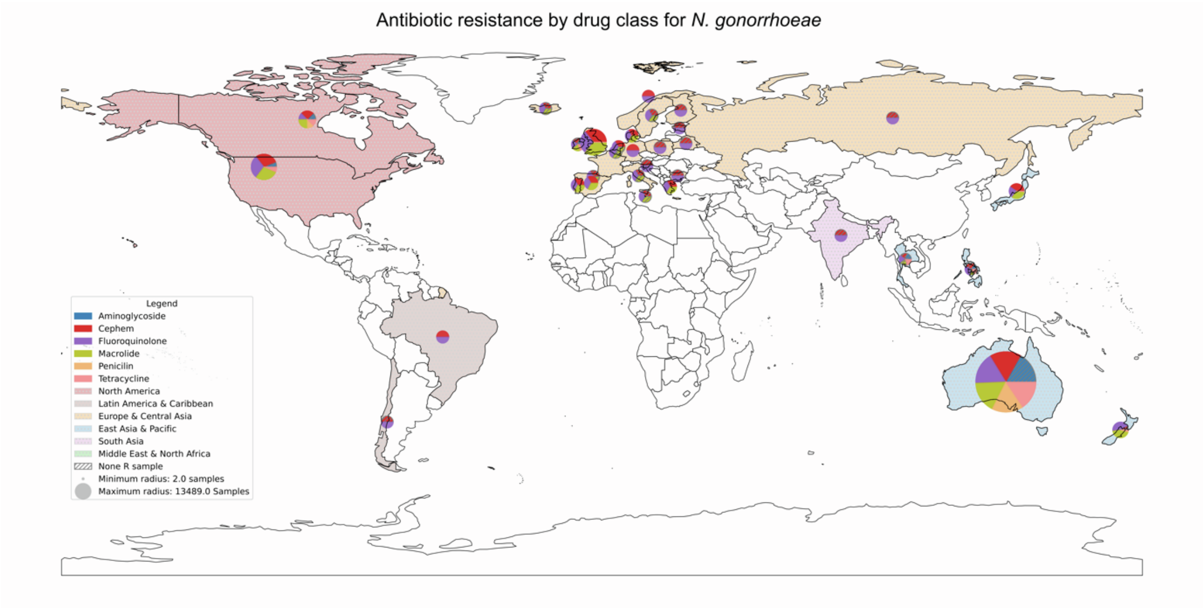

Figure S9. **Geographic distributions of 6,567 *N. gonorrhoeae* isolates with 30,741 associated resistance susceptibility phenotypic profiles obtained from the BV-BRC database[1], related to Figure 1**. The pie chart on each country shows the proportion of resistance profiles by class for each country, with the size of the chart indicating the overall number of genomes. The world map[2] has been divided according to the seven World Bank regions (East Asia and Pacific; Europe and Central Asia; Latin America and Caribbean; Middle East and North Africa; North America; South Asia; Sub-Saharan Africa).

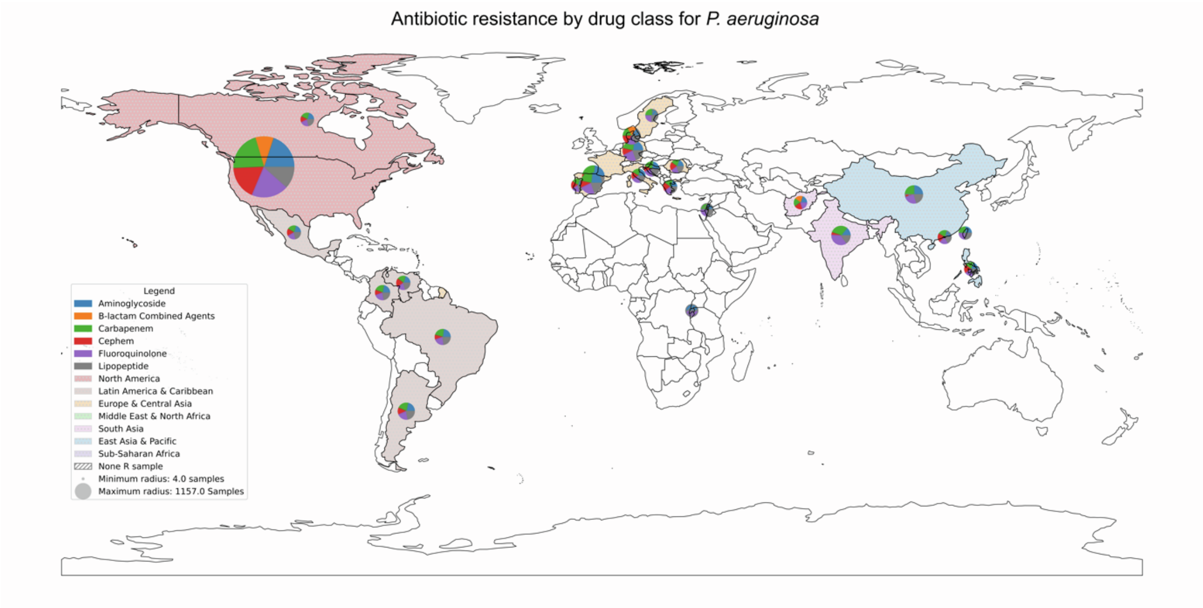

Figure S10. **Geographic distributions of 617 *P. aeruginosa* isolates with 3,531 associated resistance susceptibility phenotypic profiles obtained from the BV-BRC database[1], related to Figure 1**. The pie chart on each country shows the proportion of resistance profiles by class for each country, with the size of the chart indicating the overall number of genomes. The world map[2] has been divided according to the seven World Bank regions (East Asia and Pacific; Europe and Central Asia; Latin America and Caribbean; Middle East and North Africa; North America; South Asia; Sub-Saharan Africa).

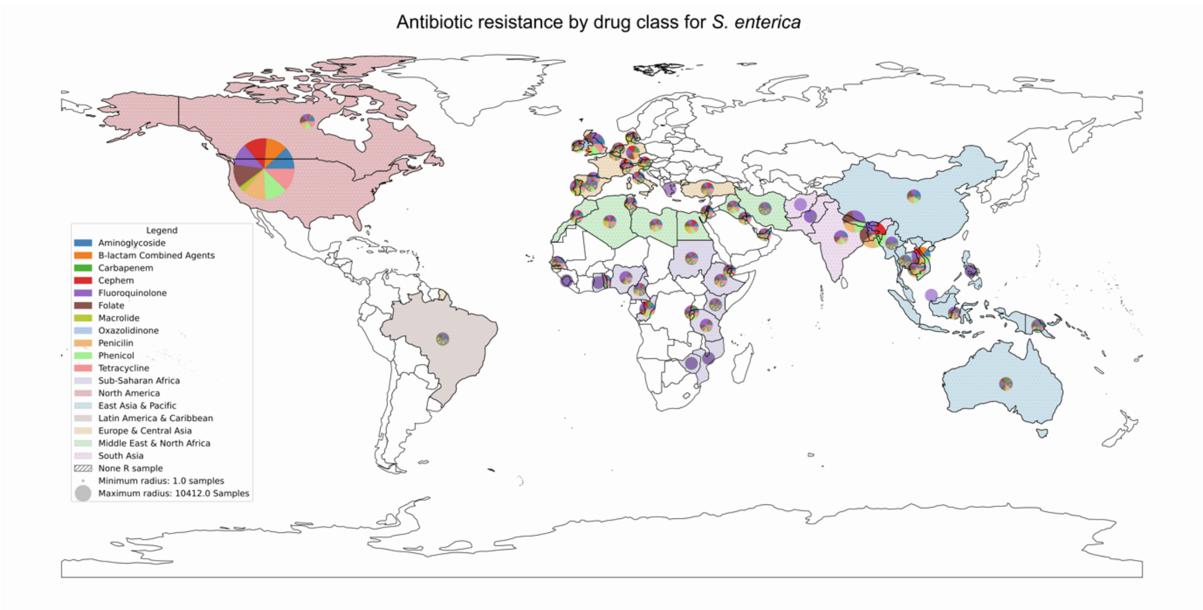

Figure S11. Geographic distributions of 3792 *S. enterica* isolates with 33,562 associated resistance susceptibility phenotypic profiles obtained from the BV-BRC database[1], related to Figure 1. The pie chart on each country shows the proportion of resistance profiles by class for each country, with the size of the chart indicating the overall number of genomes. The world map[2] has been divided according to the seven World Bank regions (East Asia and Pacific; Europe and Central Asia; Latin America and Caribbean; Middle East and North Africa; North America; South Asia; Sub-Saharan Africa).

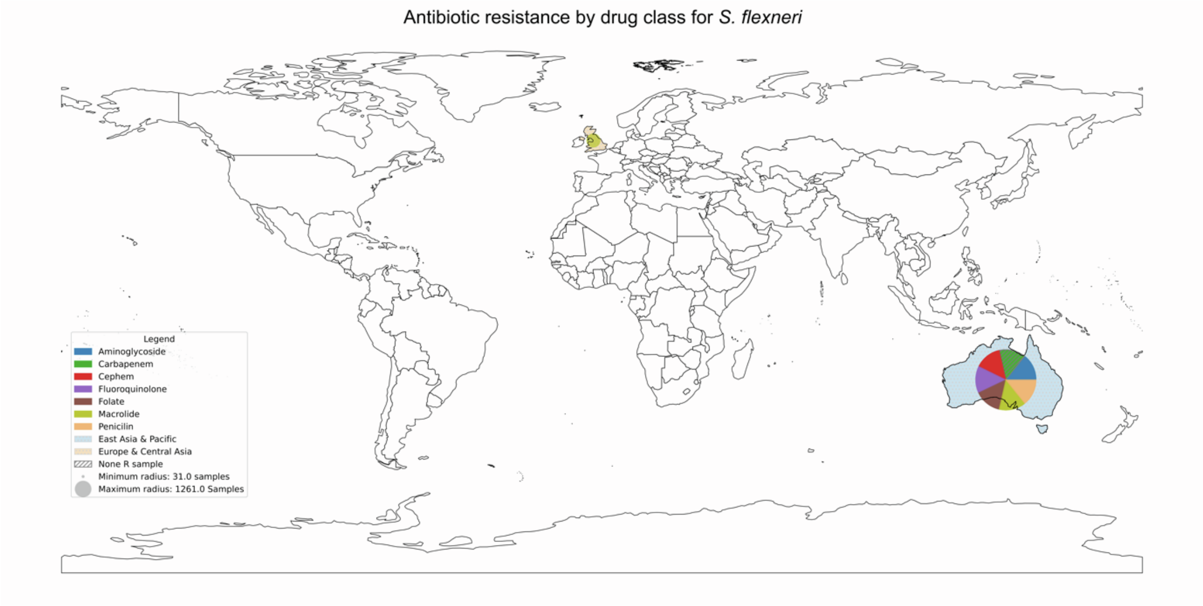

Figure S12. Geographic distributions of 202 *S. flexneri* isolates with 1,399 associated resistance susceptibility phenotypic profiles obtained from the BV-BRC database[1], related to Figure 1. The pie chart on each country shows the proportion of resistance profiles by class for each country, with the size of the chart indicating the overall number of genomes. The world map[2] has been divided according to the seven World Bank regions (East Asia and Pacific; Europe and Central Asia; Latin America and Caribbean; Middle East and North Africa; North America; South Asia; Sub-Saharan Africa).

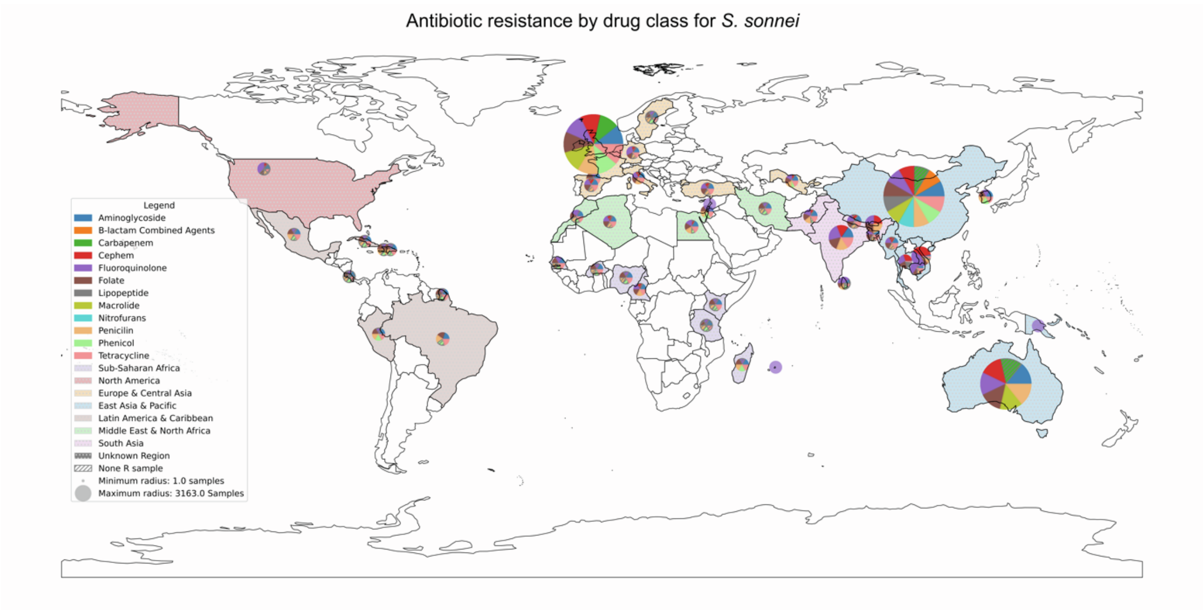

Figure S13. Geographic distributions of 1591 *S. sonnei* isolates with 17,617 associated resistance susceptibility phenotypic profiles obtained from the BV-BRC database[1], related to Figure 1. The pie chart on each country shows the proportion of resistance profiles by class for each country, with the size of the chart indicating the overall number of genomes. The world map[2] has been divided according to the seven World Bank regions (East Asia and Pacific; Europe and Central Asia; Latin America and Caribbean; Middle East and North Africa; North America; South Asia; Sub-Saharan Africa).

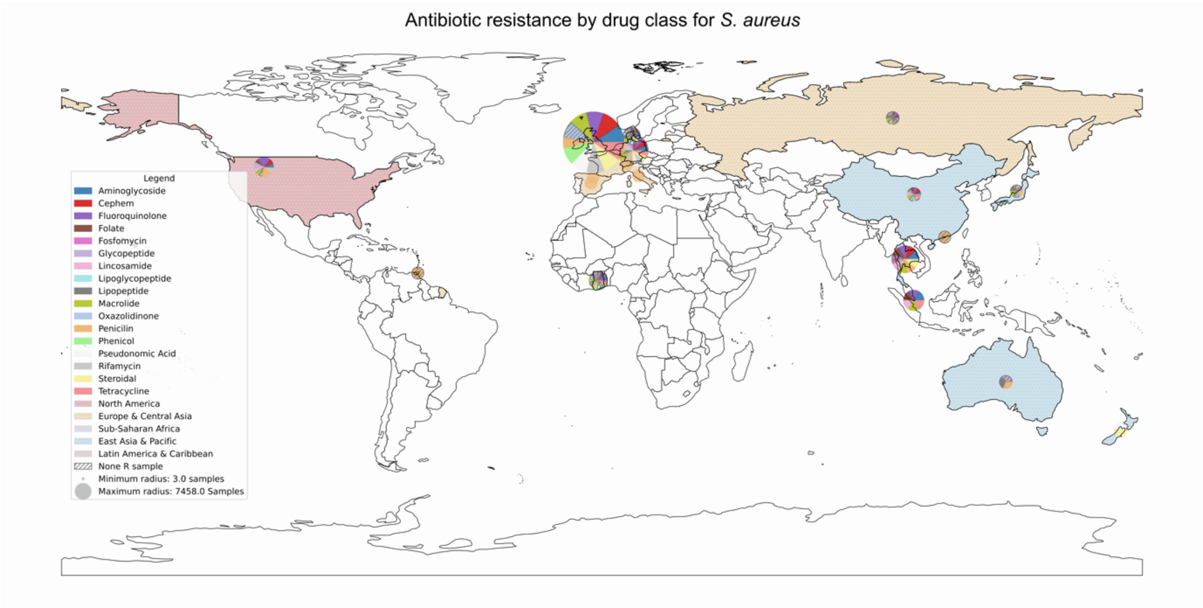

Figure S14. Geographic distributions of 3,133 *S. aureus* isolates with 18,839 associated resistance susceptibility phenotypic profiles obtained from the BV-BRC database[1], related to Figure 1. The pie chart on each country shows the proportion of resistance profiles by class for each country, with the size of the chart indicating the overall number of genomes. The world map[2] has been divided according to the seven World Bank regions (East Asia and Pacific; Europe and Central Asia; Latin America and Caribbean; Middle East and North Africa; North America; South Asia; Sub-Saharan Africa).

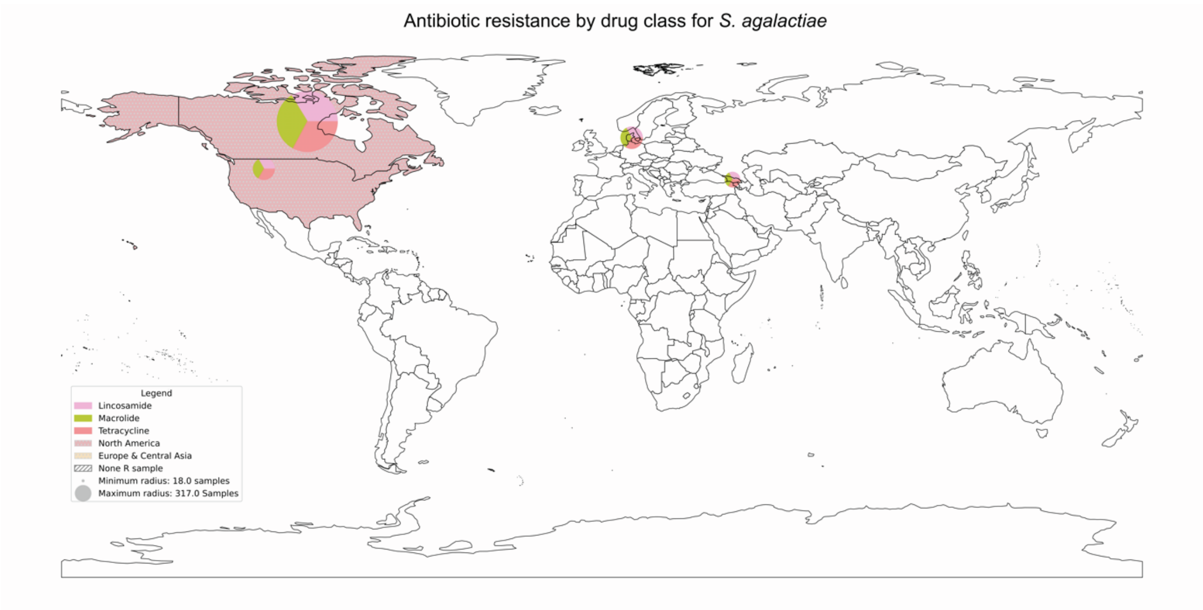

Figure S15. Geographic distributions of 154 *S. agalactiae* isolates with 458 associated resistance susceptibility phenotypic profiles obtained from the BV-BRC database[1], related to Figure 1. The pie chart on each country shows the proportion of resistance profiles by class for each country, with the size of the chart indicating the overall number of genomes. The world map[2] has been divided according to the seven World Bank regions (East Asia and Pacific; Europe and Central Asia; Latin America and Caribbean; Middle East and North Africa; North America; South Asia; Sub-Saharan Africa).

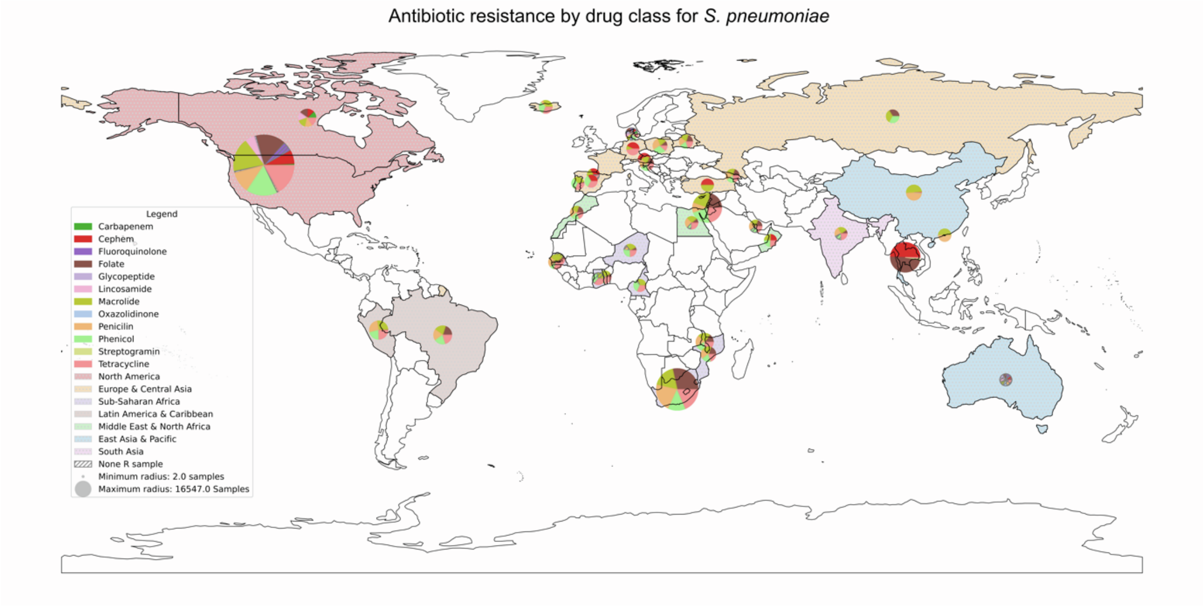

Figure S16. Geographic distributions of 13,647 *S. pneumoniae* isolates with 52,756 associated resistance susceptibility phenotypic profiles obtained from the BV-BRC database[1], related to Figure 1. The pie chart on each country shows the proportion of resistance profiles by class for each country, with the size of the chart indicating the overall number of genomes. The world map[2] has been divided according to the seven World Bank regions (East Asia and Pacific; Europe and Central Asia; Latin America and Caribbean; Middle East and North Africa; North America; South Asia; Sub-Saharan Africa).

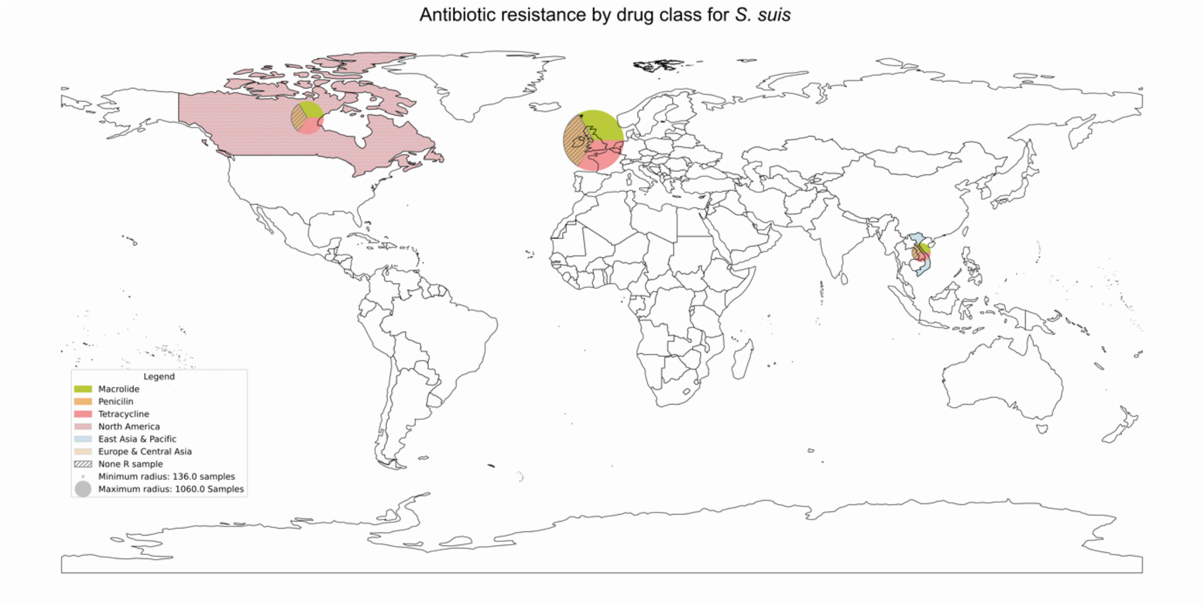

Figure S17. Geographic distributions of 550 *S. suis* isolates with 2,180 associated resistance susceptibility phenotypic profiles obtained from the BV-BRC database[1], related to Figure 1. The pie chart on each country shows the proportion of resistance profiles by class for each country, with the size of the chart indicating the overall number of genomes. The world map[2] has been divided according to the seven World Bank regions (East Asia and Pacific; Europe and Central Asia; Latin America and Caribbean; Middle East and North Africa; North America; South Asia; Sub-Saharan Africa).

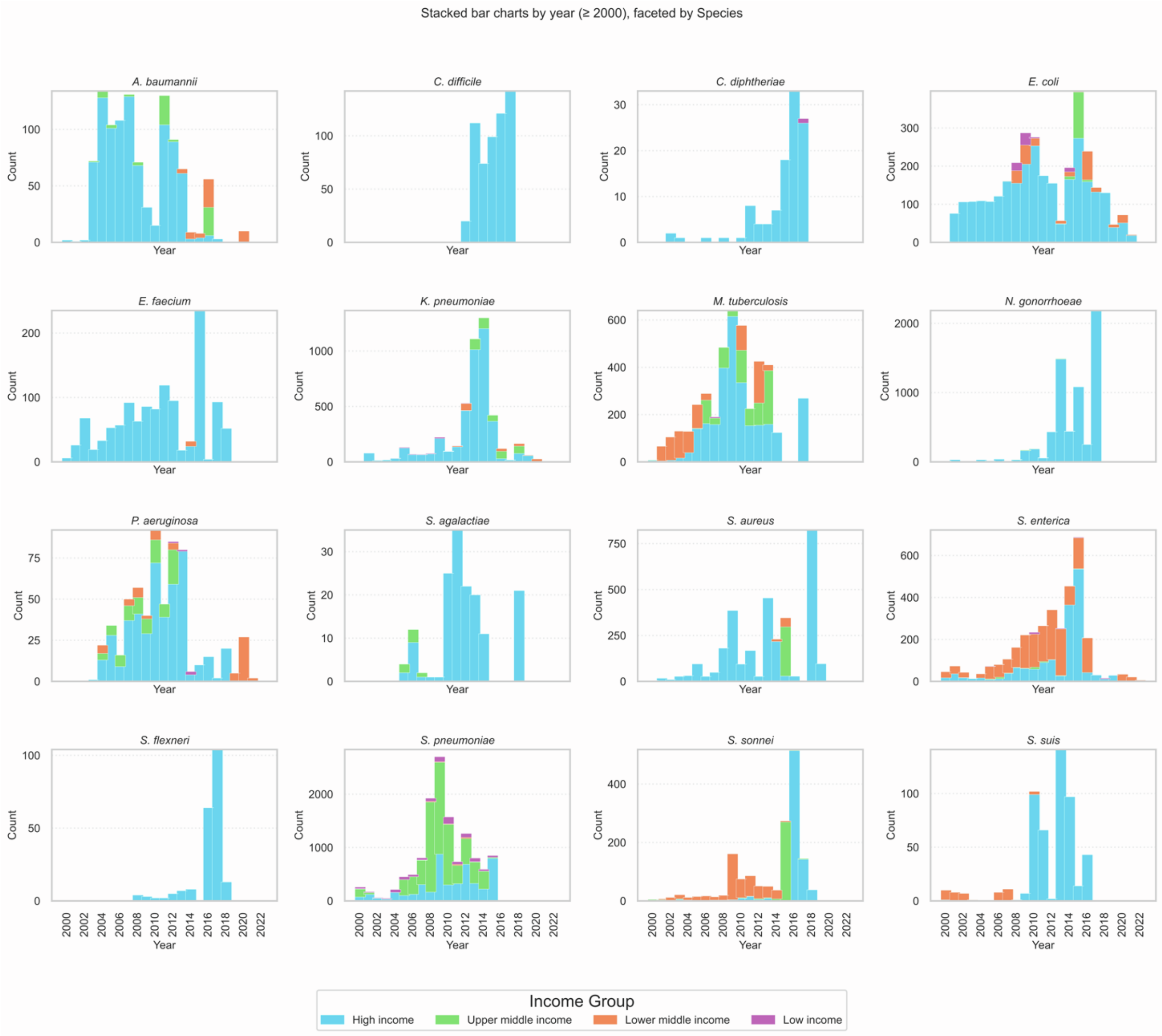

Figure S18. Stacked bar charts showing the distribution of sequence data across years by income group, related to STAR Methods. Data are truncated to only show data from the year 2000 onwards for visual clarity, one subpanel is shown for each bacterial species, stratified by World Bank income group, based on the isolates used, **Table S1**. Counts are stacked by income group: high income (blue), upper middle income (green), lower middle income (orange), and low income (red).

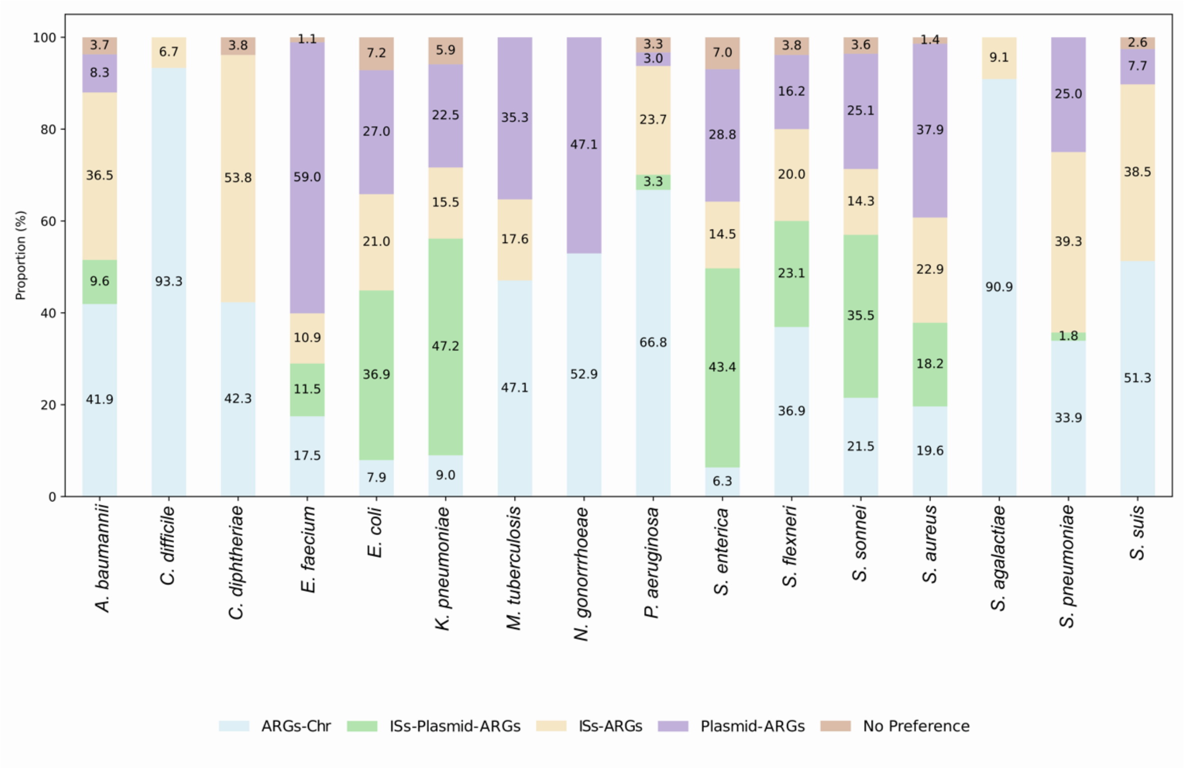

Figure S19. **Stacked bar plot showing the proportion of AMR genomic traits and their predominant location across 16 bacterial species, related to Figure 1.** Each feature can be present in four locations (ARG located in the chromosome = ARGs-Chr, ARG located in the chromosome and integrated within an IS = ISs-ARGs, ARG located in the plasmid and integrated within an IS = ISs-Plasmid-ARGs and ARGs present on plasmids but not within an insertion sequence = Plasmid-ARGs). The proportion of isolates carrying the AMR genomic trait at each location is given in **Table S5**. If more than 50% of the isolates contain the AMR genomic trait in a specific location, this location is considered predominant, otherwise it is labelled as no preference.

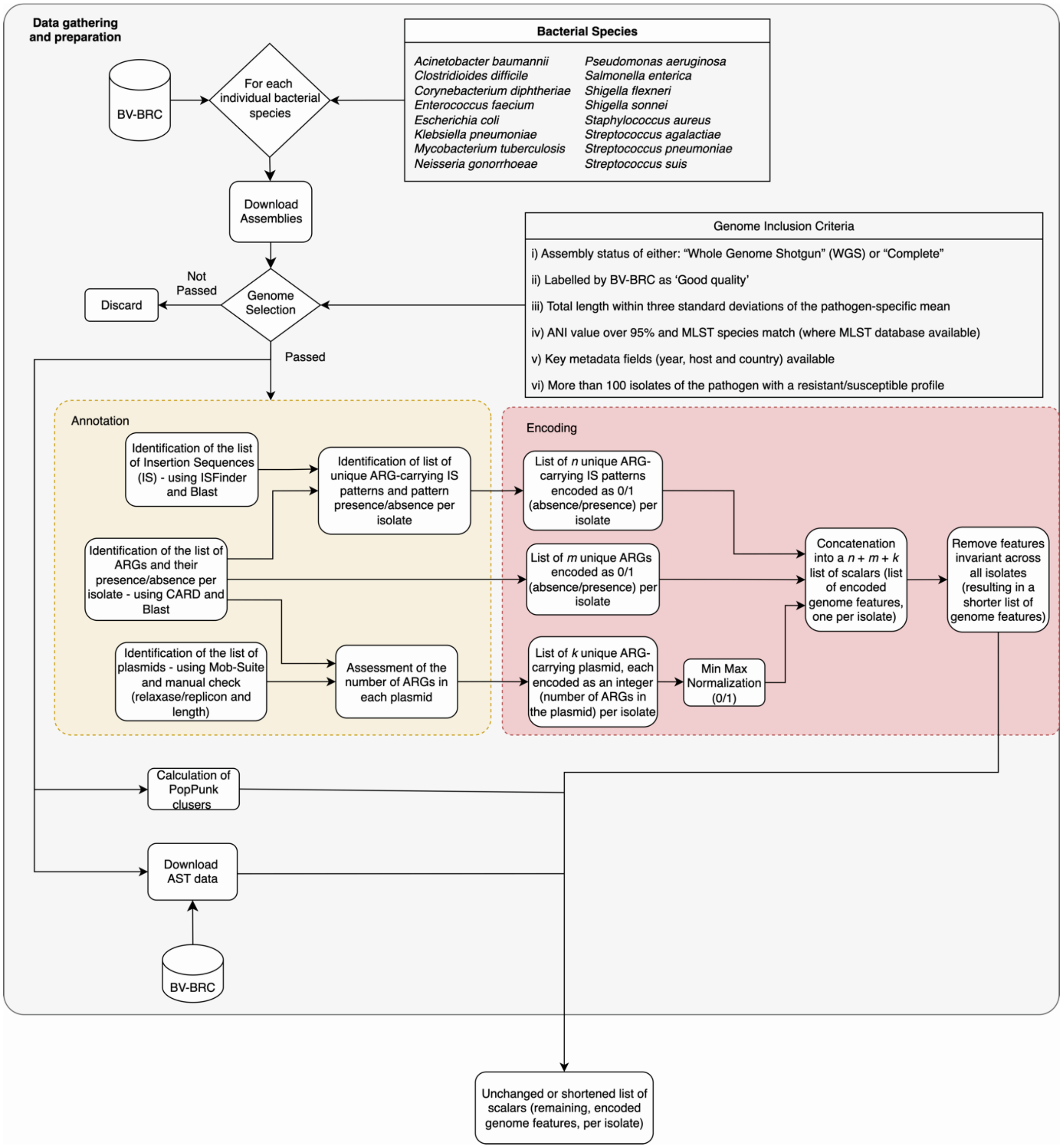

Figure S20. Data analysis and processing part 1: Data gathering and preparation, related to Figure 2. Activities are grouped into modules, represented as panels of different colours; the arrows represent the data flow.

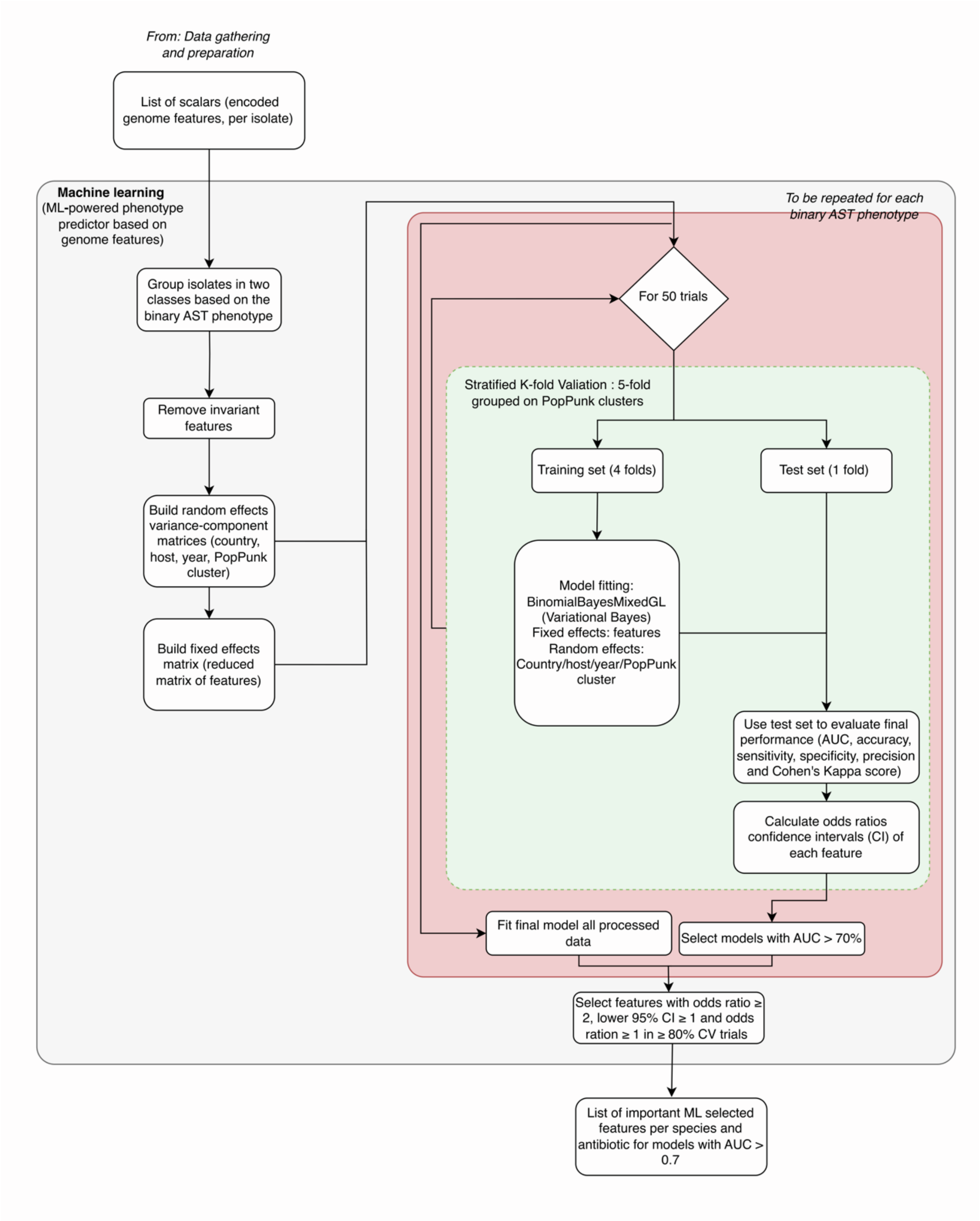

Figure S21. Data analysis and processing part 2: Machine learning, related to Figure 2. Activities are grouped into modules, represented as panels of different colours; the arrows represent the data flow.

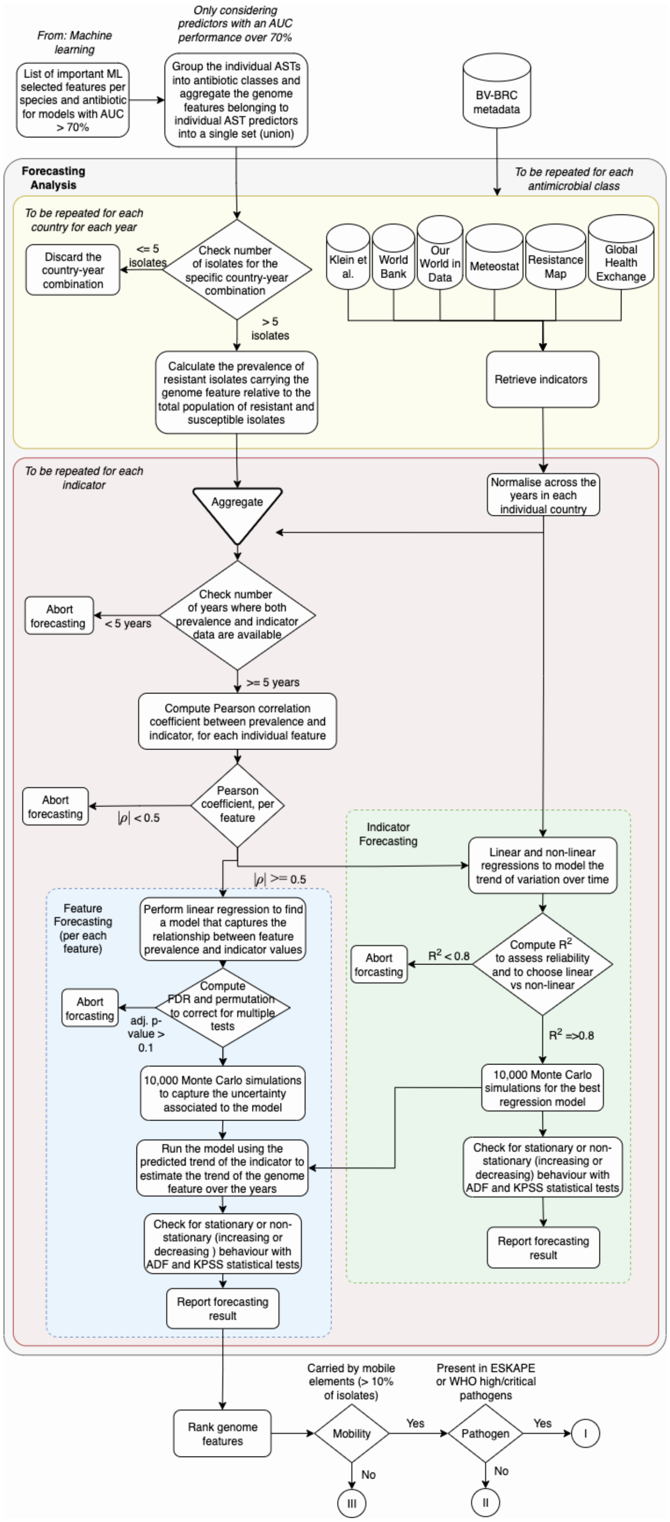

Figure S22. Data analysis and processing part 3: Forecasting analysis, related to Figure 2. Activities are grouped into modules, represented as panels of different colours; the arrows represent the data flow.

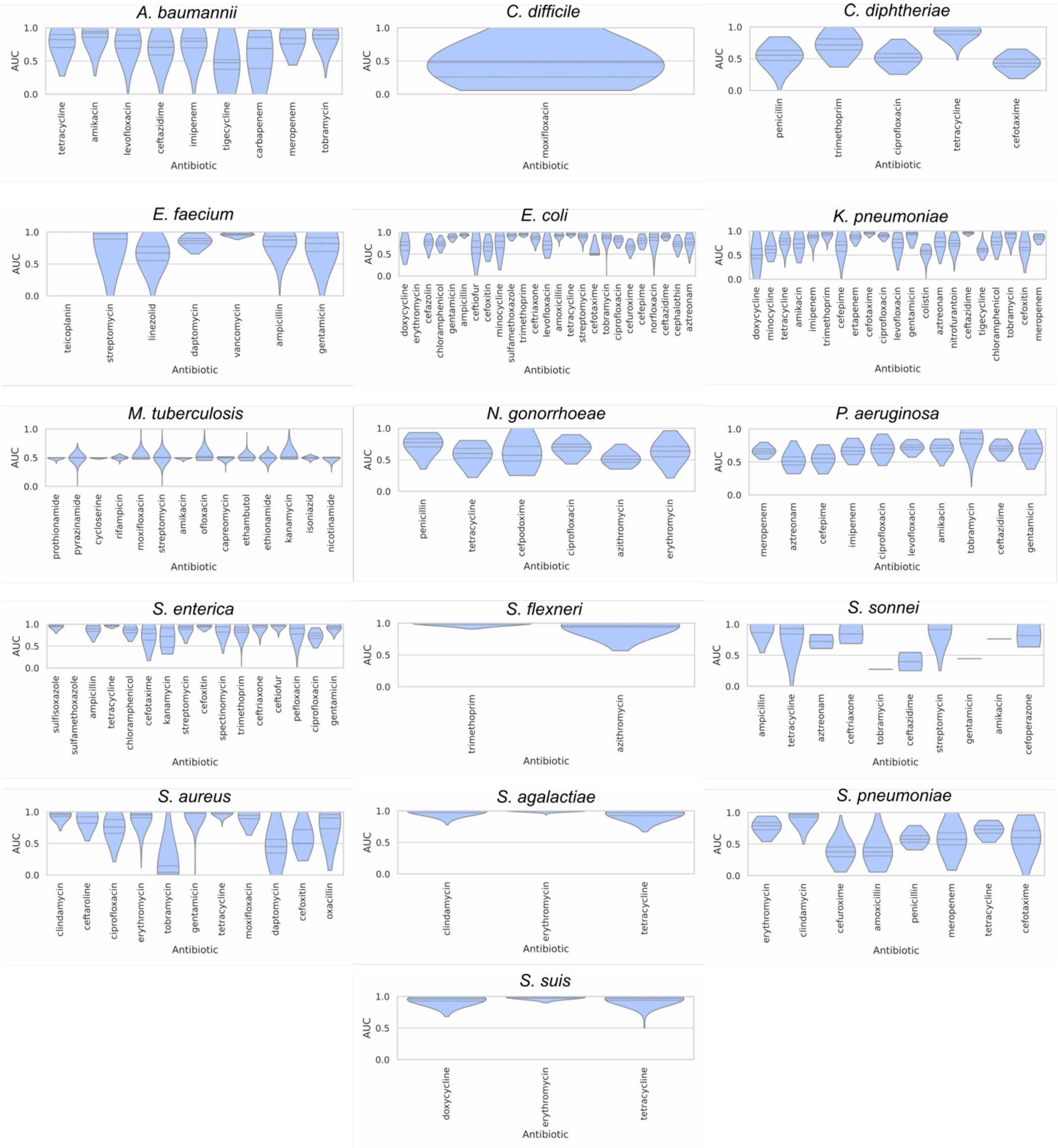

Figure S23. **Machine learning performance AUC by species and antibiotic model, related to STAR Methods.** The supervised ML pipeline predicts the resistance/susceptibility profiles of all 16 bacterial species. ML performance results are given for the area under the curve AUC, calculated from 50 training runs for each antimicrobial model.

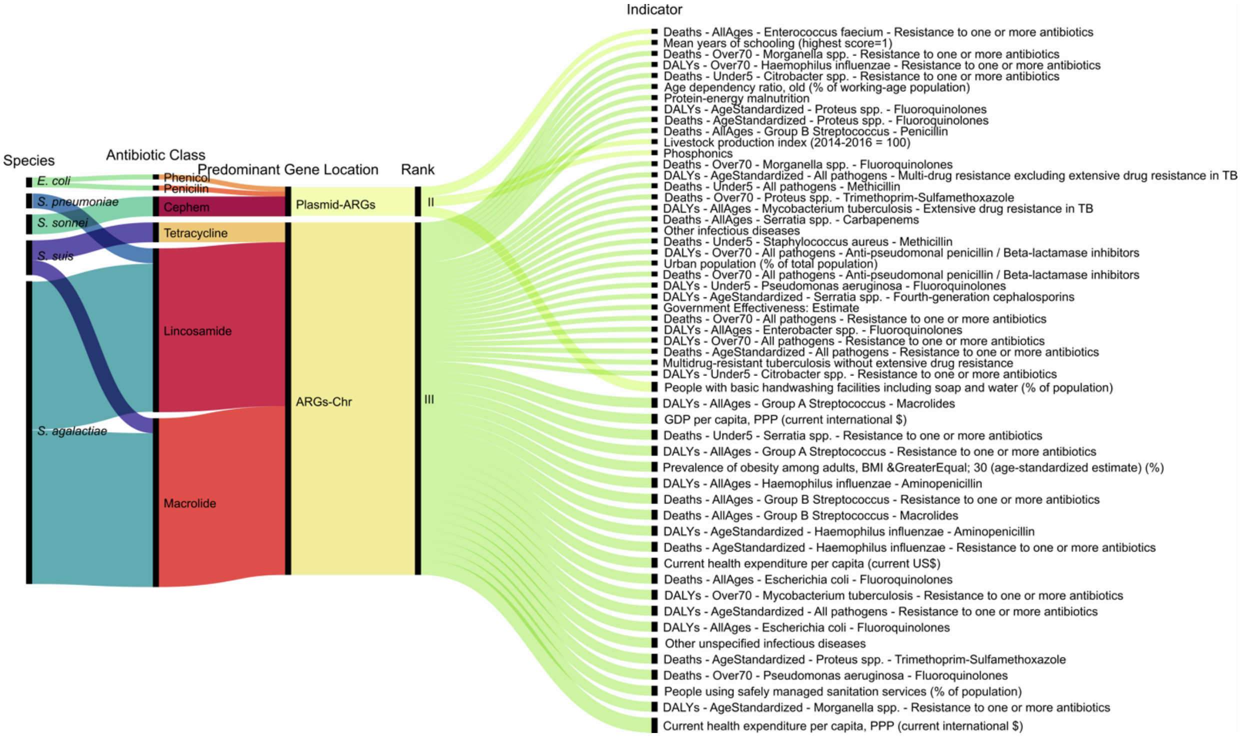

Figure S24. Sankey flow diagram illustrating forecast associations involving the ARG *ermB,* related to STAR Methods. The diagram depicts relationships between *ermB*, the bacterial species in which it was selected, the associated antibiotic classes identified by the machine learning models, the predominant gene location, feature rank (I/II), and the corresponding indicators. Only associations where *ermB* was selected by the ML models and linked to increasing or stationary indicators are shown. Line thickness reflects the number of distinct indicators connected to each node.

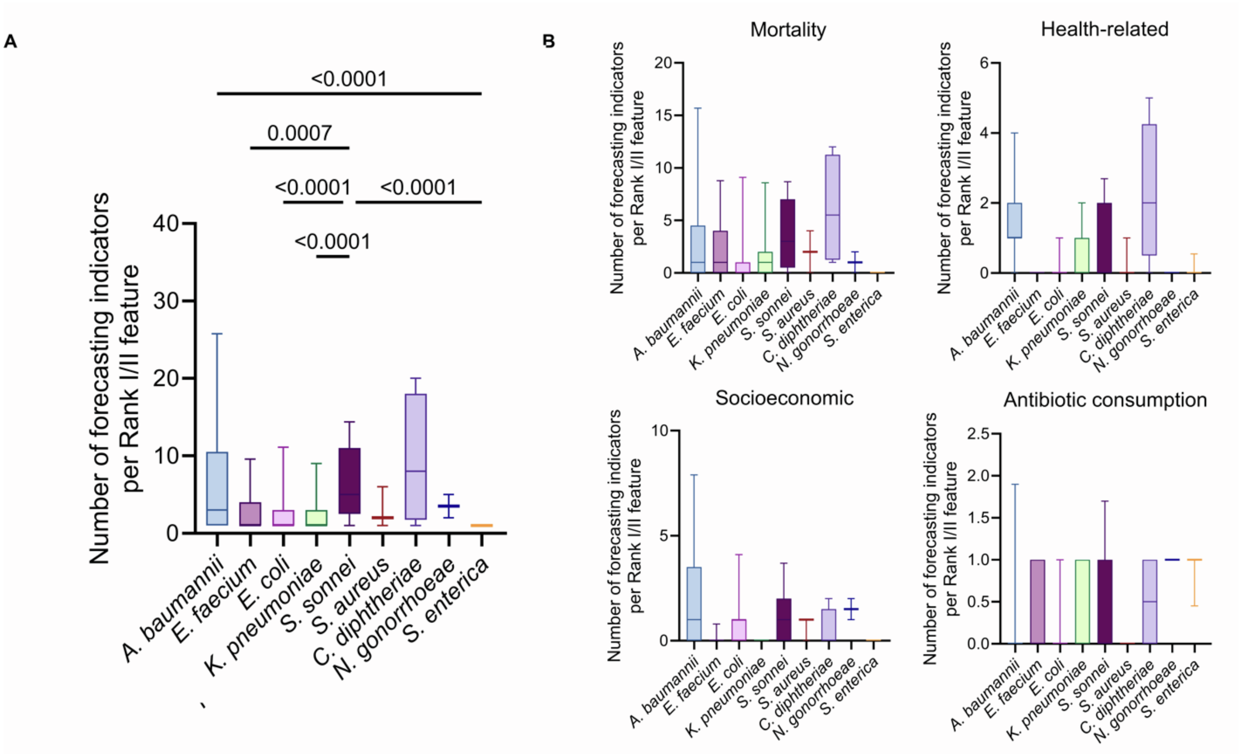

Figure S25. **Number of increasing or stationary forecasting indicators per rank I/II genomic feature, related to Figure 5.** (A) overall number of forecasting indicators per feature including antibiotic consumption, health-related, socioeconomic, environmental and mortality indicators. Significant statistical differences shown as line with adjusted *p*-value above plot (Kruskal-Wallis, two-tailed). (B) number of forecasting indicators per feature shown separately for antibiotic consumption, health-related, socioeconomic and mortality indicators. In each plot the box shows the interquartile range of the data with the median denoted by a horizontal line, and the whiskers indicate the range.

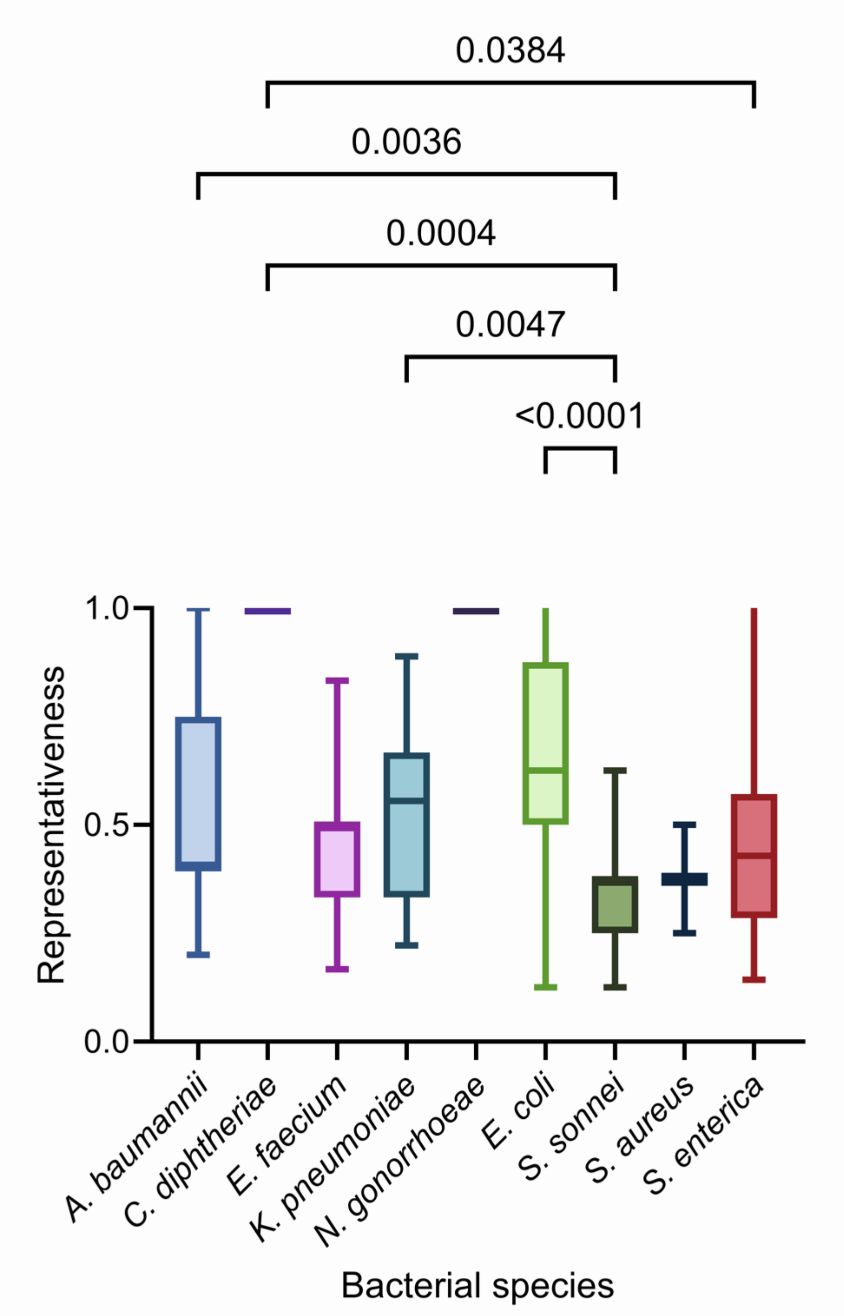

Figure S26. **Boxplot of the representativeness of the selected rank I/II features across each pathogen relative to the ML models, related to Figure 3.** For each pathogen we calculated a representativeness coefficient for the selected rank I/II features, defined as the number of resistance classes (in aggregated ML models with an AUC > 0.7) in which the feature was selected, relative to the number in which it was present in the input. For each pathogen, the box shows the interquartile range of the data with the median denoted by a horizonal line, and the whiskers indicate the range. Significantly different distributions are indicated on the plot with horizontal brackets and the adjusted *p*-value given (Kruskal-Wallis, two-tailed, Dunn's adj. *p*-values).

K. pneumoniae

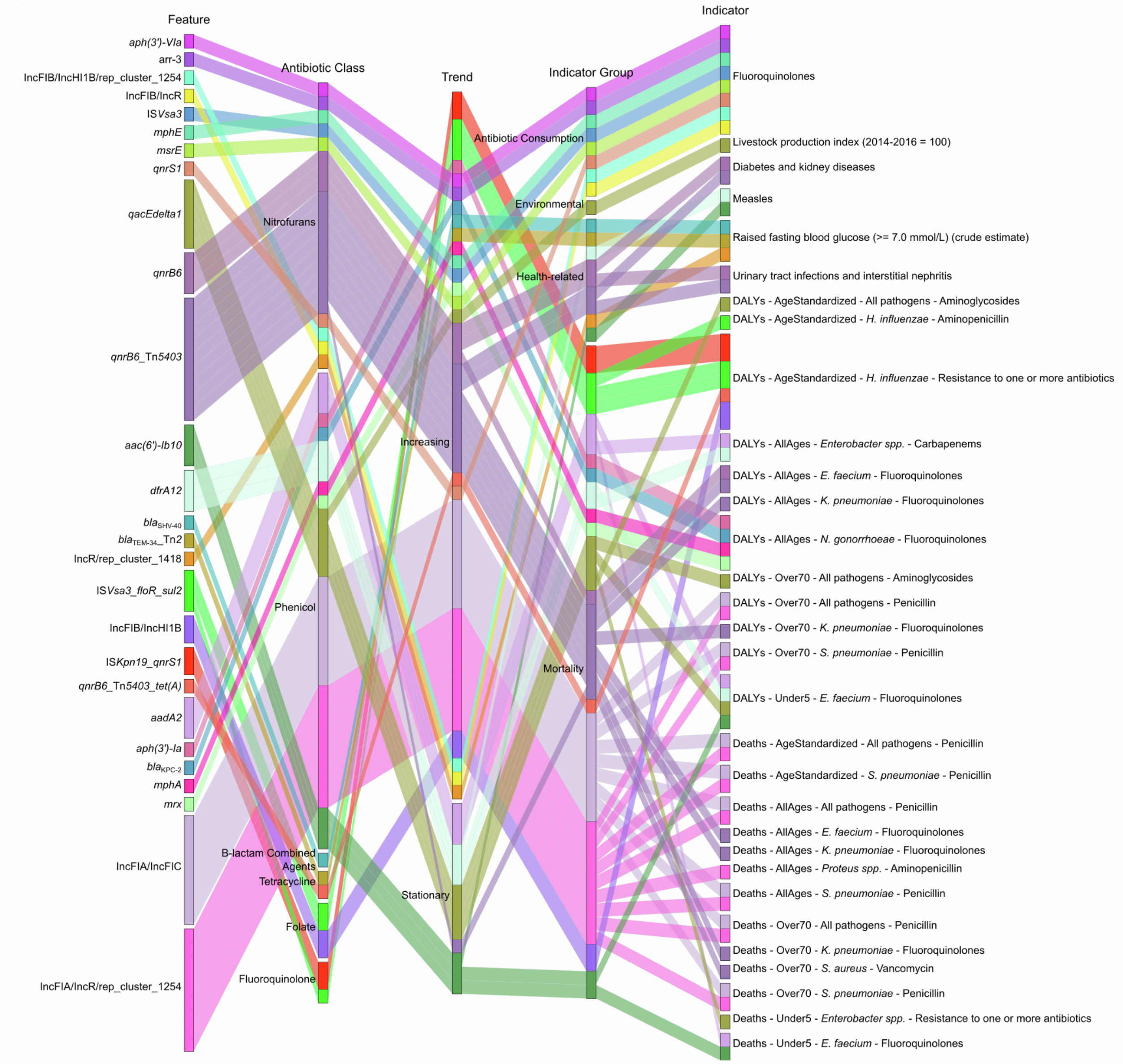

Figure S27. *K. pneumoniae* Sankey flow diagram, related to Figure 5. This Sankey diagram illustrates the relationships between AMR genomic features (Feature), their associated antibiotic class identified via ML (Antibiotic Class), the projected trend of each indicator (whether the indicator was forecast to increase or remain stationary, Trend), indicator groups (Indicator Group), and specific indicators (Indicator) within each group. Only rank I/II features associated with increasing or stationary indicators are displayed. The thickness of the links represents the number of different indicators, while the colours correspond to the associated genomic feature.

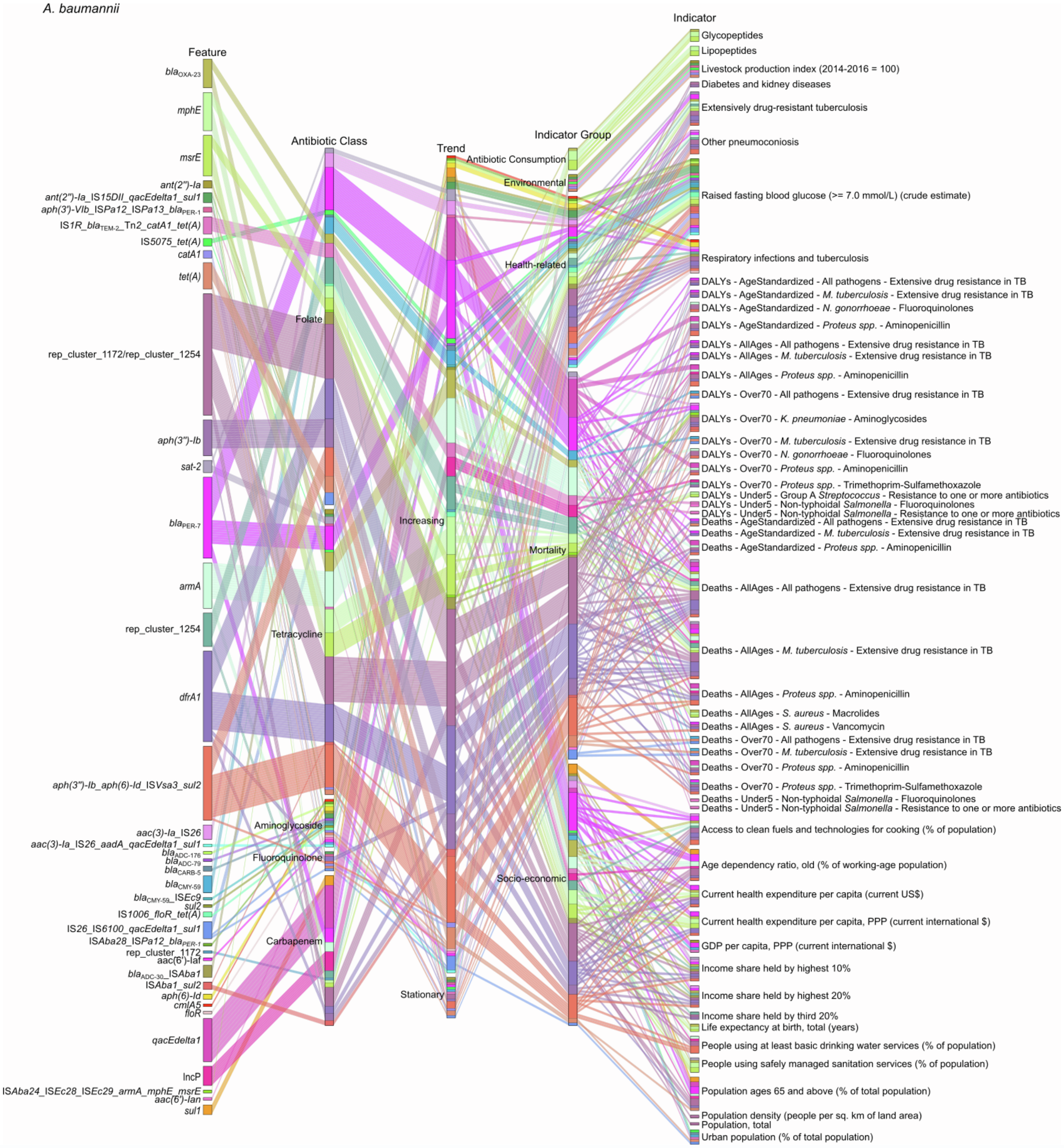

Figure S28. ***A. baumannii Sankey*** **flow diagram, related to Figure 5.** This Sankey diagram illustrates the relationships between AMR genomic features (Feature), their associated antibiotic class identified via ML to each feature (Antibiotic Class), the projected trend of each indicator (whether the indicator was forecast to increase or remain stationary, Trend), indicator groups (Indicator Group), and specific indicators (Indicator) within each group. Only rank I/II features associated with increasing or stationary indicators are displayed. The thickness of the links represents the number of different indicators, while the colours correspond to the associated genomic feature.

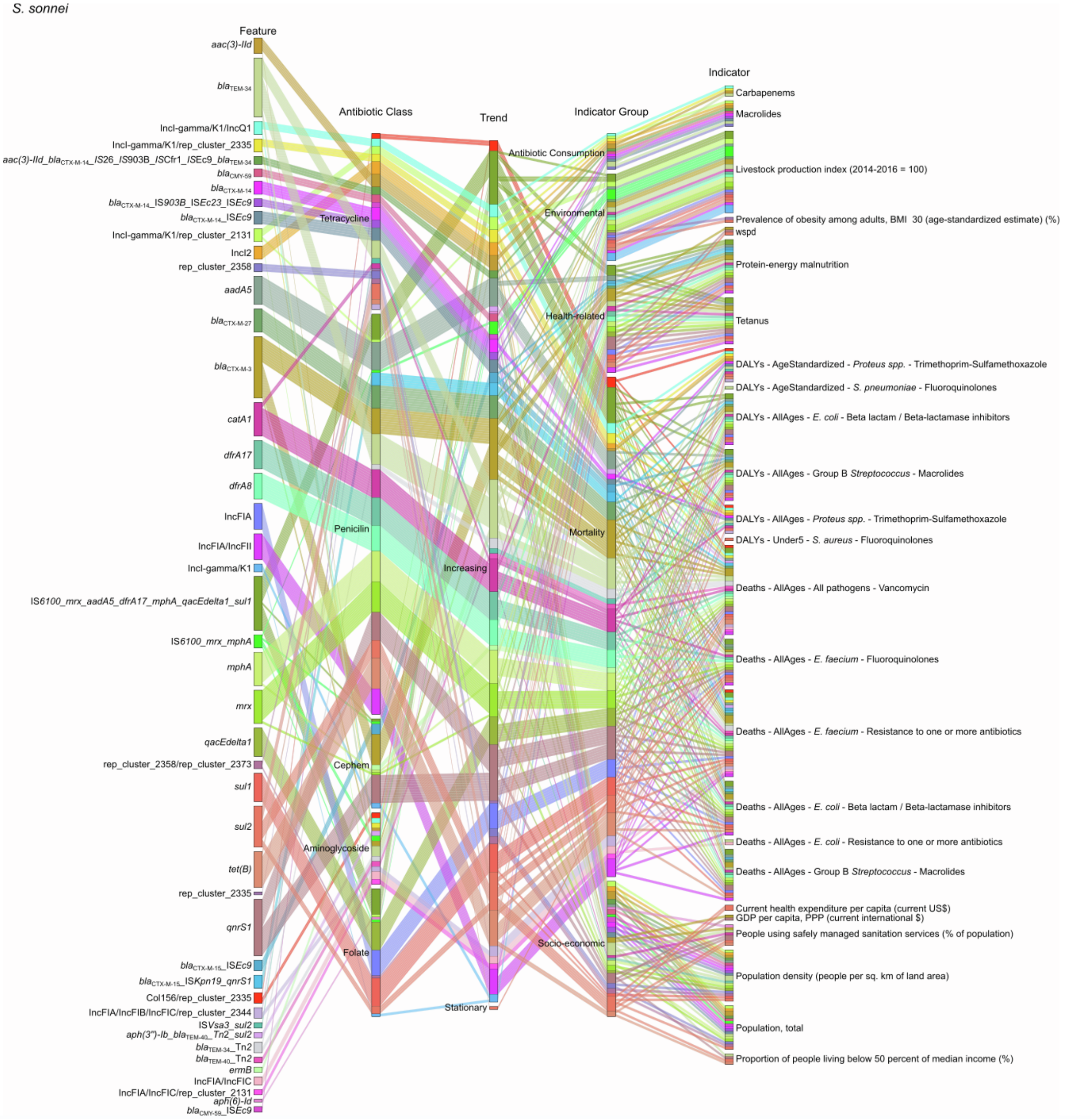

Figure S29. ***S. sonnei*** **Sankey flow diagram related to Figure 5.** This Sankey diagram illustrates the relationships between AMR genomic features (Feature), their associated antibiotic class identified via ML to each feature (Antibiotic Class), the projected trend of each indicator (whether the indicator was forecast to increase or remain stationary, Trend), indicator groups (Indicator Group), and specific indicators (Indicator) within each group. Only rank I/II features associated with increasing or stationary indicators are displayed. The thickness of the links represents the number of different indicators, while the colours correspond to the associated genomic feature.

E. coli

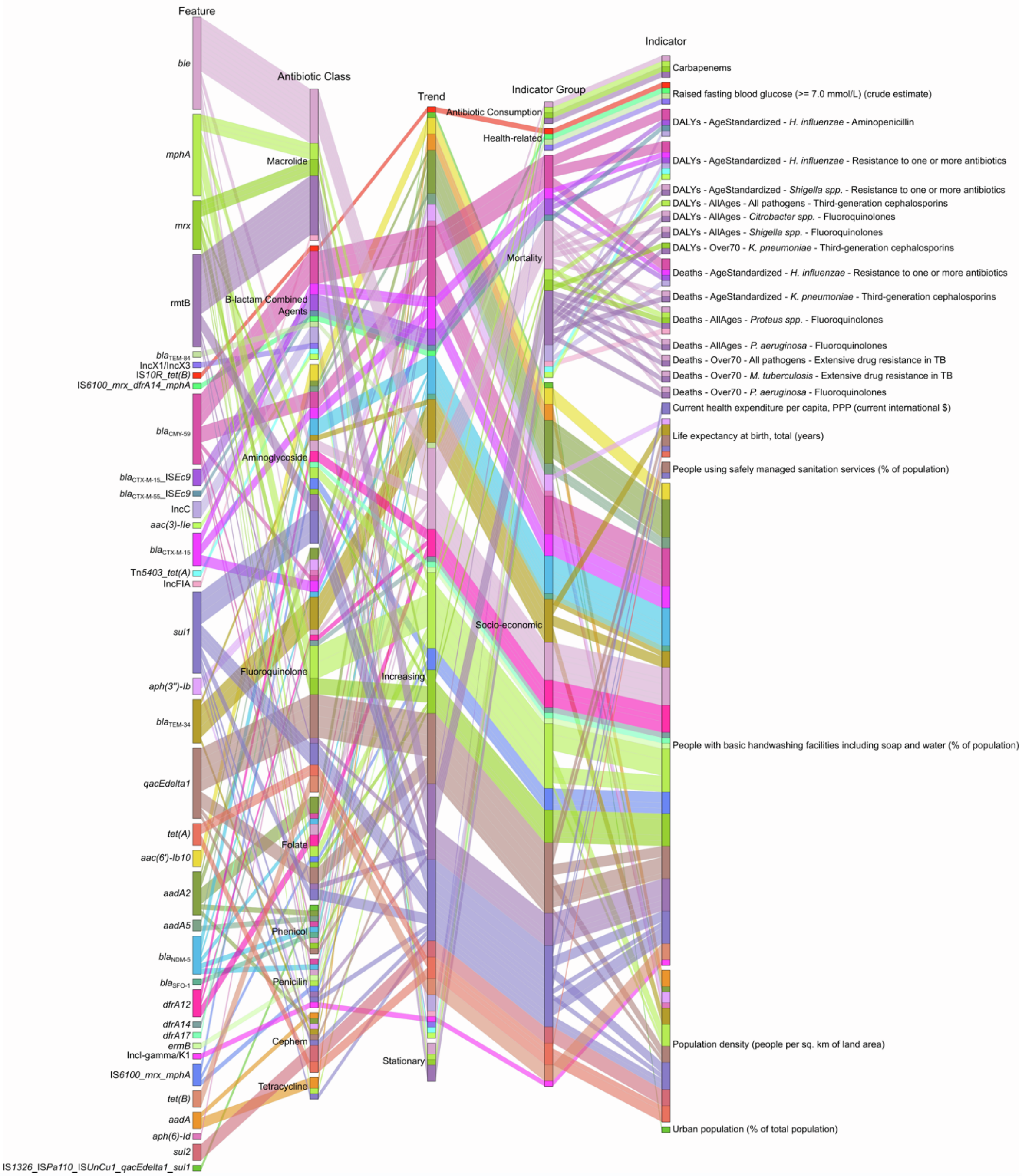

Figure S30. ***E. coli* Sankey flow diagram, related to Figure 5.** This Sankey diagram illustrates the relationships between AMR genomic features (Feature), their associated antibiotic class identified via ML to each feature (Antibiotic Class), the projected trend of each indicator (whether the indicator was forecast to increase or remain stationary, Trend), indicator groups (Indicator Group), and specific indicators (Indicator) within each group. Only rank I/II features associated with increasing or stationary indicators are displayed. The thickness of the links represents the number of different indicators, while the colours correspond to the associated genomic feature.

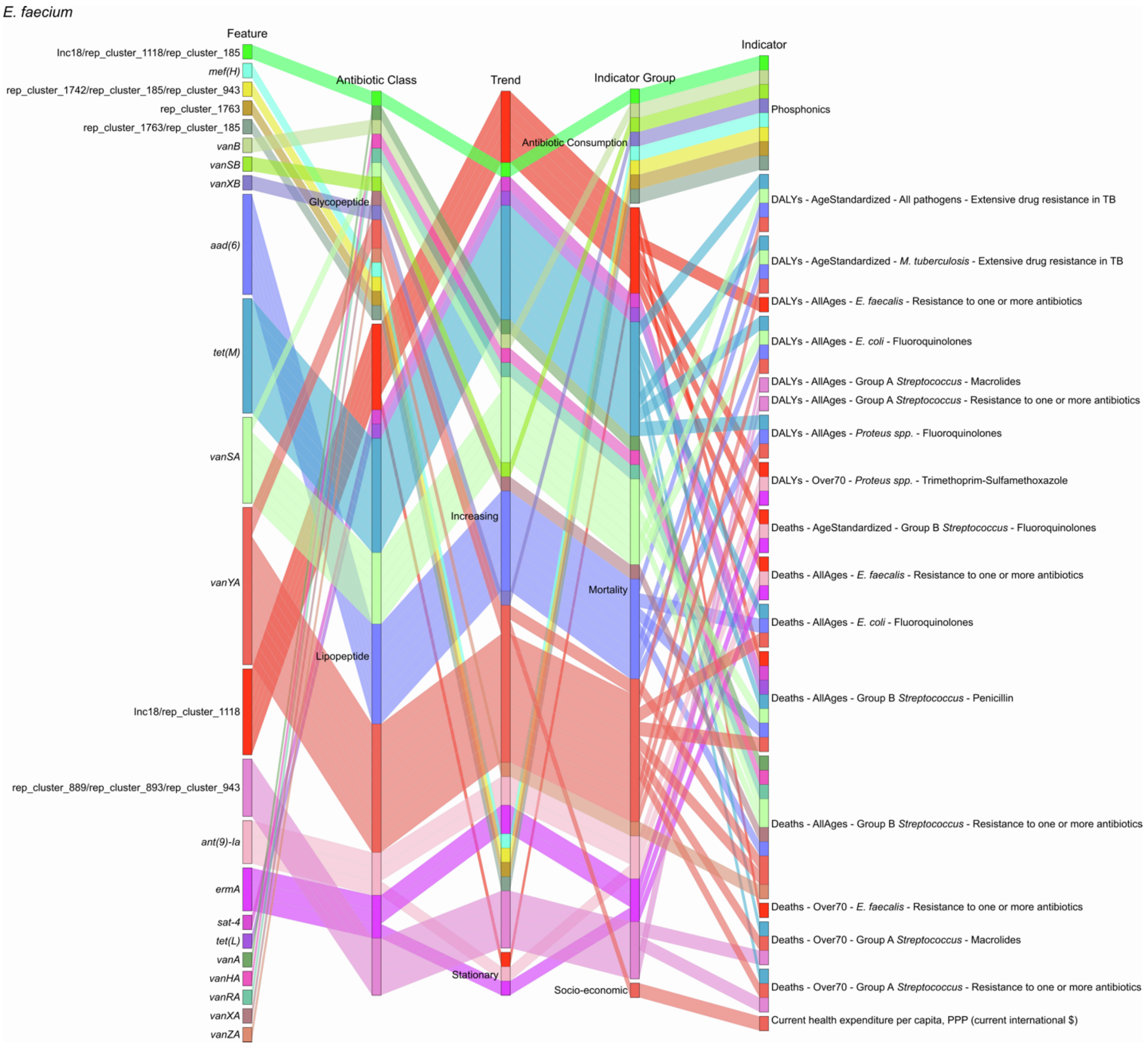

Figure S31. *E. faecium* Sankey flow diagram, related to Figure 5. This Sankey diagram illustrates the relationships between AMR genomic features (Feature), their associated antibiotic class identified via ML to each feature (Antibiotic Class), the projected trend of each indicator (whether the indicator was forecast to increase or remain stationary, Trend), indicator groups (Indicator Group), and specific indicators (Indicator) within each group. Only rank I/II features associated with increasing or stationary indicators are displayed. The thickness of the links represents the number of different indicators, while the colours correspond to the associated genomic feature.

S. enterica

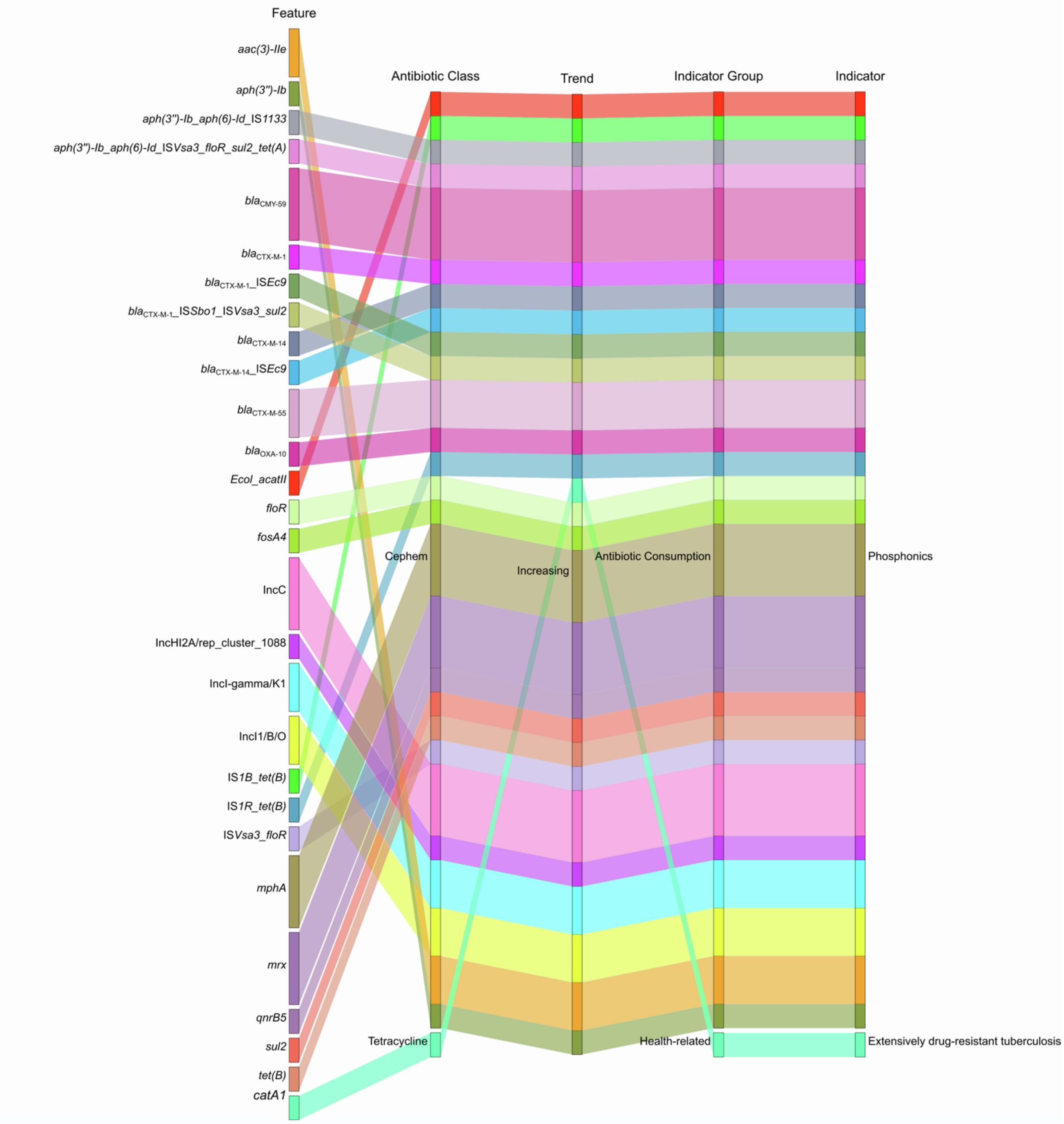

Figure S32. *S. enterica* Sankey flow diagram, related to Figure 5. This Sankey diagram illustrates the relationships between AMR genomic features (Feature), their associated antibiotic class identified via ML to each feature (Antibiotic Class), the projected trend of each indicator (whether the indicator was forecast to increase or remain stationary, Trend), indicator groups (Indicator Group), and specific indicators (Indicator) within each group. Only rank I/II features associated with increasing or stationary indicators are displayed. The thickness of the links represents the number of different indicators, while the colours correspond to the associated genomic feature.

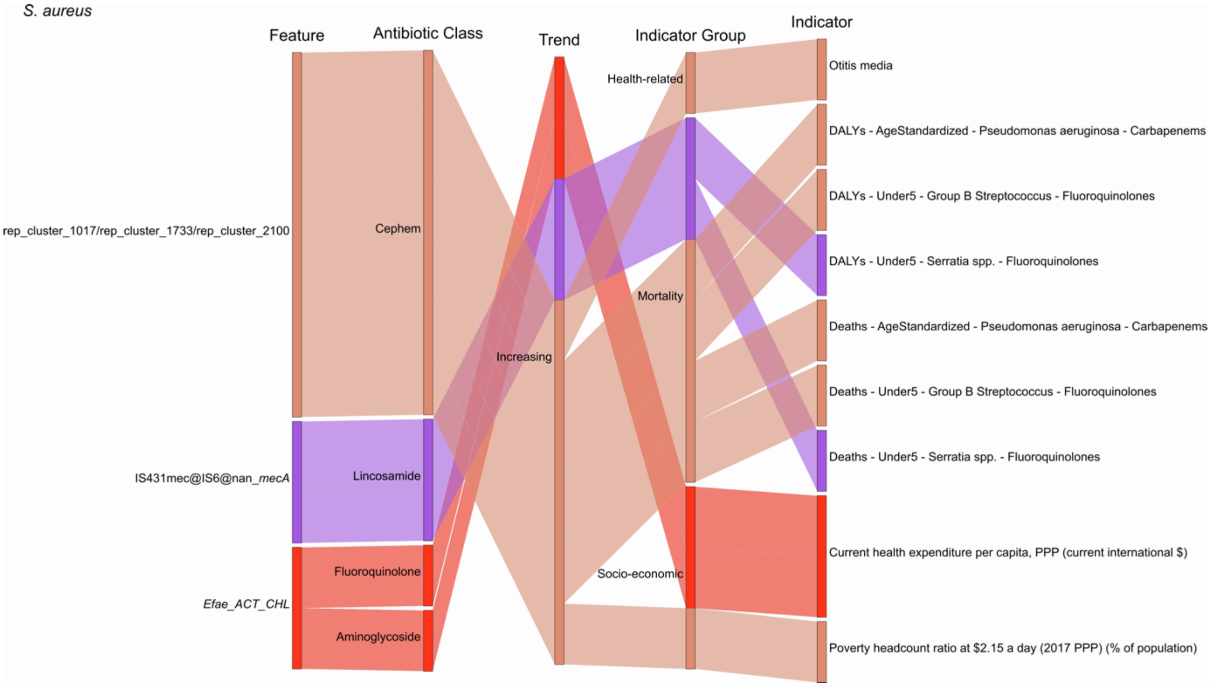

Figure S33. *S. aureus* Sankey flow diagram, related to Figure 5. This Sankey diagram illustrates the relationships between AMR genomic features (Feature), their associated antibiotic class identified via ML to each feature (Antibiotic Class), the projected trend of each indicator (whether the indicator was forecast to increase or remain stationary, Trend), indicator groups (Indicator Group), and specific indicators (Indicator) within each group. Only rank I/II features associated with increasing or stationary indicators are displayed. The thickness of the links represents the number of different indicators, while the colours correspond to the associated genomic feature.

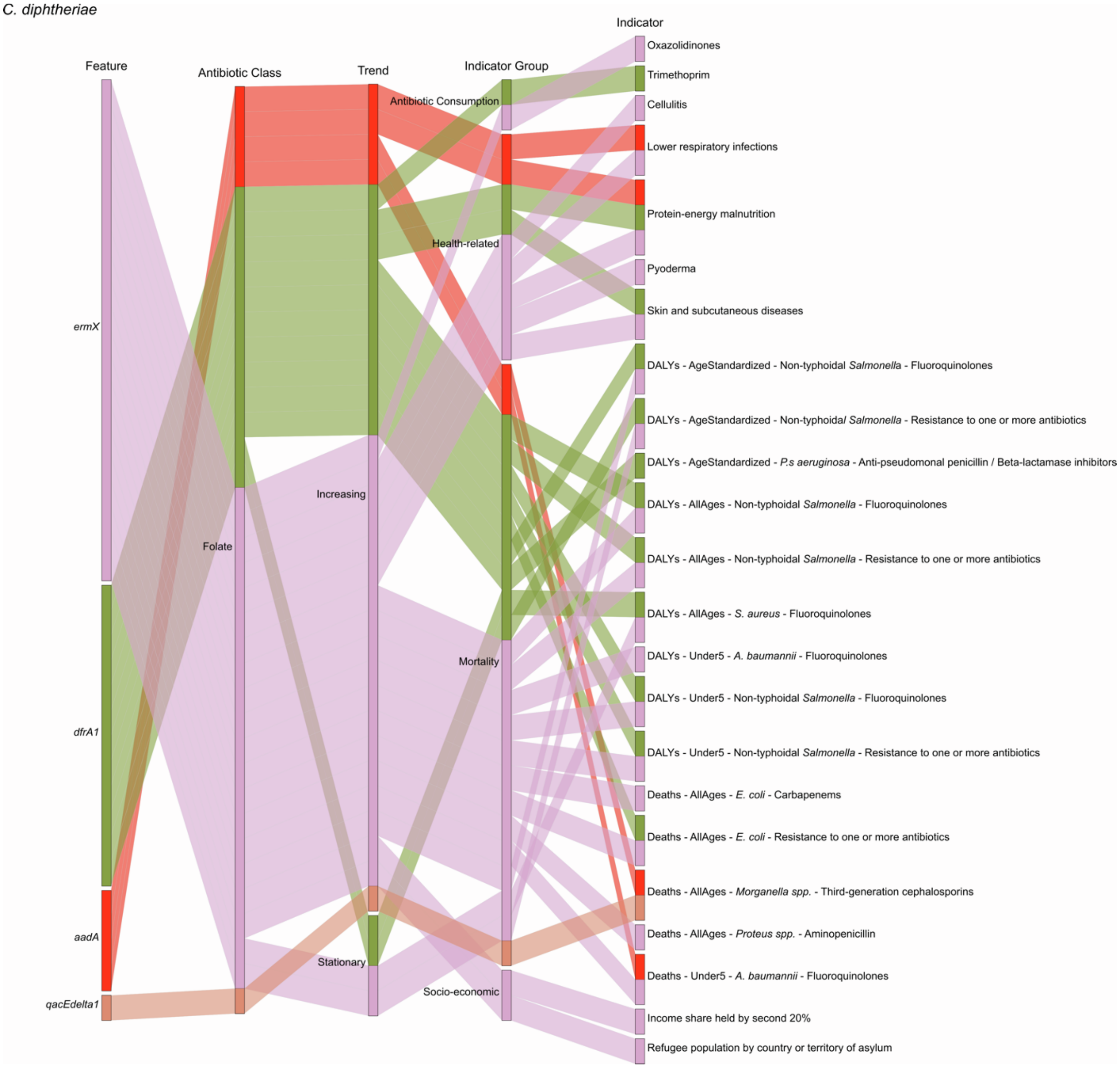

Figure S34. *C. diphtheriae* Sankey flow diagram, related to Figure 5. This Sankey diagram illustrates the relationships between AMR genomic features (Feature), their associated antibiotic class identified via ML to each feature (Antibiotic Class), the projected trend of each indicator (whether the indicator was forecast to increase or remain stationary, Trend), indicator groups (Indicator Group), and specific indicators (Indicator) within each group. Only rank I/II features associated with increasing or stationary indicators are displayed. The thickness of the links represents the number of different indicators, while the colours correspond to the associated genomic feature.

Species and infection site (infection site for prevalence only)	Spearman correlation (data aggregated by year and country)	Spearman correlation (data aggregated by year and income group)
E. coli BSI	-0.09	-0.19
E. coli UTI	0.16	0.34
A. baumannii BSI	-0.06	0.00
K. pneumoniae BSI	-0.01	-0.14
K. pneumoniae UTI	-0.04	0.07
S. enterica BSI	0.02	-0.02
S. enterica GI	0.00	0.26
S. aureus BSI	0.03	0.18
N. gonorrhoeae UG	0.10	0.15

Table S4. **Spearman correlation coefficients between GLASS body-site-specific pathogen prevalence rates (incidence per million population) and counts of the number of sequence isolates in the study, related to STAR Methods**. Data are aggregated by country and year in column 2 and World Bank income group (FY26 classification)[3] and year in column 3.

Species	Reference Name	GenBank Accession	Use
A. baumannii	*A. baumannii* ATCC 19606	CP045110.1	ANI
C. difficile	*C. difficile* S-0253	CP076401.1	ANI
C .diphtheriae	*C. diphtheriae* NCTC11397	LN831026.1	ANI
E. faecium	*E. faecium* SRR24	CP038996.1	ANI
E. coli	*E. coli* str. K-12 substr. MG1655	U00096.3	ANI
K. pneumoniae	*K. pneumoniae* subsp. pneumoniae HS11286	CP003200.1	ANI
M. tuberculosis	*M. tuberculosis* H37Rv	AL123456.3	ANI
N. gonorrhoeae	*N. gonorrhoeae* TUM19854	AP023069.1	ANI
P. aeruginosa	*P. aeruginosa* PAO1	AE004091.2	ANI
S. enterica	*S. enterica* subsp. enterica serovar Typhimurium str. LT2	AE006468.2	ANI
S. flexneri	*S. flexneri* 2a str. 301	AE005674.2	ANI
S. sonnei	*S. sonnei* strain ATCC 29930	CP026802.1	ANI
S. aureus	*S. aureus* subsp. aureus NCTC 8325	CP000253.1	ANI
S. agalactiae	*S. agalactiae* strain NGBS128	CP012480.1	ANI
S. pneumoniae	*S. pneumoniae* assembly NCTC7465	LN831051.1	ANI
S. suis	*S. suis* BM407	FM252032.1	ANI
S. flexneri	*S. flexneri* 2a assembly ASM2962549v1	GCF_029625495.1	KmerID
S. flexneri	*S. flexneri* assembly ASM2235464v1	GCF_022354645.1	KmerID
S. flexneri	*S. flexneri* assembly ASM295033v1	GCF_002950335.1	KmerID
S. flexneri	*S. flexneri* G1663 assembly ASM102185v1	GCF_001021855.1	KmerID
S. flexneri	*S. flexneri* 1b assembly ASM3050607v1	GCF_030506075.1	KmerID
S. flexneri	*S. flexneri* 1a assembly ASM157812v1	GCF_001578125.1	KmerID
S. flexneri	*S. flexneri* assembly ASM2473232v1	GCF_024732325.1	KmerID
S. flexneri	*S. flexneri* assembly ASM2235396v1	GCF_022353965.1	KmerID
S. flexneri	*S. flexneri* 1bassembly ASM3050605v1	GCF_030506055.1	KmerID
S. flexneri	*S. flexneri* 2a str 2457T	NC_004741.1	KmerID
S. flexneri	*S. flexneri* 2002017	GCA_000022245.1	KmerID
S. sonnei	*S. sonnei* assembly ASM972999v1	GCF_009729995.1	KmerID
S. sonnei	*S. sonnei* assembly ASM360642v1	GCF_003606425.1	KmerID
S. sonnei	*S. sonnei* assembly ASM3829866v1	GCF_038298665.1	KmerID

S. sonnei	*S. sonnei* assembly ASM3704248v1	GCF_037042485.2	KmerID
S. sonnei	*S. sonnei* assembly ASM966451v1	GCF_009664515.1	KmerID
S. sonnei	*S. sonnei* assembly ASM3836717v1	GCF_038367175.1	KmerID
S. sonnei	*S. sonnei* assembly ASM3836735v1	GCF_038367355.1	KmerID
S. sonnei	*S. sonnei* assembly ASM244253v1	GCF_002442535.1	KmerID
S. sonnei	*S. sonnei* assembly ASM2590845v1	GCF_025908455.1	KmerID
S. sonnei	*S. sonnei* Ss046	NC_007384.1	KmerID
S. sonnei	*S. sonnei* 53G	GCA_000283715.1	KmerID

Table S20. Reference genomes and accessions used for ANI- and KmerID-based species validation of genomes included in the study, related to STAR Methods.

Species	Total ARG-IS Combinations	Mean Isolates per ARG-IS	Standard Deviation	Range	Mean Isolates for Selected Features (rank I/II)
A. baumannii	187	8.7	36.2	1-455	25.3
C. difficile	2	1.0	0.0	-	-
C. diphtheriae	11	5.6	10.0	1-35	35.0
E. faecium	41	3.7	5.7	1-29	-
E. coli	584	6.1	26.2	1-384	77.2
K. pneumoniae	769	10.4	56.0	1-1006	95.0
M. tuberculosis	2	3.0	2.8	1-5	-
N. gonorrhoeae	0	-	-	-	-
P. aeruginosa	67	2.3	6.0	1-48	-
S. enterica	256	6.4	14.0	1-116	21.9
S. flexneri	45	4.4	11.4	1-77	-
S. sonnei	106	8.1	29.5	1-208	49.2
S. aureus	88	17.1	95.3	1-880	880.0
S. agalactiae	1	1.0	-	-	-
S. suis	23	6.0	15.0	1-72	-

Table S21. **Number and descriptive statistics (mean, standard deviation and range) of ARG-carrying IS combinations in the machine learning inputs and the mean number of isolates per ARG-carrying IS combination, related to STAR Methods.**

Supplemental Data 1: Forecasting analysis of *M. tuberculosis*, *N. gonorrhoeae*, and *C. difficile*

The machine learning pipeline was unable to robustly correlate AMR phenotypes with AMR genomic features in *M. tuberculosis*, *N. gonorrhoeae*, and *C. difficile*. Despite this, given the public-health importance of these pathogens, forecasting analysis was conducted, considering all AMR genomic features found in these pathogens, using the same pipeline as shown in **Figure S22**. Only a small number of features were forecast to increase in prevalence in association with increasing/stationary indicators in each species (**Table S9**), seven in *M. tuberculosis* (*aac(2')-Ic*, *bla$_C$*, *efpA*, *erm(37), mfpA*, *rv1877*, *rv2856*), all associated with cycloserine resistance; two in *N. gonorrhoeae* (IncP, bla$_{TEM-135}$) associated with fluoroquinolone and penicillin resistance; and four in *C. difficile* (*cfrE*, *aph(3')-IIIa, ermB, sat-4*) associated with fluoroquinolone resistance.

The genomic feature *ermB* in *C. difficile* was associated with 144 indicators of which 91.6% were mortality or health-related, reflecting the importance of *C. difficile* as a nosocomial infection[4]. In *M. tuberculosis*, 18 indicators were associated with each of the seven genomic features. Of these indicators 11 were mortality indicators, three health-related and four were socioeconomic. Notably, two of the health-related indicators were related to diabetes risk factors (raised blood glucose and obesity levels), suggesting that the well-established comorbid relationship between *M. tuberculosis* and diabetes[5] is associated with increasing resistance in this species. Increasing prevalence of *N. gonorrhoeae* was associated with three mortality indicators, two socioeconomic indicators and antibiotic consumption of lipopeptides. Notably both socioeconomic indicators were related to increasing elderly populations ("population ages 65 and above" and "age dependency ratio"), potentially reflecting a growing concern of increasing rates of *N. gonorrhoeae* infections among older individuals[6] and an increased likelihood of resistant strains being isolated in older individuals[7].

Supplemental Methods 1

When using mixed indicator sources, definitional heterogeneity is an inherent limitation of global-scale analyses and cannot be completely removed. In this analysis, we sought to minimise this by restricting our data sources to repositories that apply standardised collection, curation, and quality-control procedures, collate data at a global level, and provide a clear definition and source for each indicator used (Table S2). Each data provider used in this study employs extensive data harmonisation and validation.

Importantly, indicators were analysed independently within the forecasting framework and were not aggregated into composite indices spanning heterogeneous repositories. Each indicator entered the model as a separate temporal trajectory.

All indicators were scaled between 0 and 1 prior to modelling to standardise numerical ranges and avoid artefacts arising from differences in measurement units. This scaling harmonises magnitude but does not alter construct definitions. Associations are therefore interpreted directionally (increasing, decreasing or stationary) rather than as absolute cross-domain effect size comparisons.

Countries with insufficient temporal overlap between genomic data and indicator time series were excluded to ensure adequate longitudinal alignment.

Specifically:

- World Bank: standardises data using harmonised accounting frameworks, alignment to national standards, statistical modelling of missing values, and cross validation procedures. Although data gaps and definitional heterogeneity cannot be fully resolved, the World Bank applies harmonised accounting frameworks and metadata standards that support cross-country trend analysis, which aligns with the trend-based forecasting objective of this study (https://datatopics.worldbank.org/world-development-indicators/sources-and-methods.html)
- ResistanceMap" antibiotic consumption data are derived from IQVIA MIDAS sales data curated from national sample surveys and converted into defined daily doses using WHO methodology (https://resistancemap.onehealthtrust.org/MethodologyAU.php).
-OurWorldinData: applies a robust and scalable data-cleaning, unit-harmonisation, age-standardisation and quality-control pipelines (https://github.com/owid/etl).
- GHDx: provides extensive metadata, including coverage, and definitions, standardised country coding, age-standardised estimates, harmonised units, and multi-layer validation of completeness and plausibility (https://www.healthdata.org/data-tools-practices/data-collection).

Robustness to Coverage and Source Heterogeneity

To assess whether the completeness across source types differed and mixing repositories disproportionately influenced the results, we examined the distribution of retained high-priority feature–indicator associations across indicator source categories (World Bank, ResistanceMap, Our World in Data, GHDx). Across repositories there were no statistical differences in the completeness of non-mortality indicators, only differences between mortality (GHDx) and non-mortality indicators (Kruskal-Wallis test: $H = 926.4$, $p = 5.14 \times 10^{-198}$; Dunn's post-hoc test with Bonferroni correction: GHDx vs Klein *et al*., Meteostat, Our World in Data, ResistanceMap and World Bank all adjusted *p*-values ≈ 0; Klein *et al.* vs Meteostat, Our World in Data, ResistanceMap and World Bank all adjusted *p*-values ≈ 1; Meteostat vs Our World in Data and ResistanceMap adjusted *p*-values ≈ 1; Meteostat vs World Bank adjusted *p*-value = 0.9573; Our World in Data vs ResistanceMap and World Bank all adjusted *p*-values ≈ 1; ResistanceMap vs World Bank adjusted *p*-value = 0.3985). Additionally, we assessed the forecastable associations relative to the number of input indicators across repositories, for those with greater than 10 indicators (World Bank, Our World in Data, GHDx) using two-tailed pairwise proportio*n Z-tests* (*GHDx vs ResistanceMap: Z = 0.511, p*-value=0.610; *GHDx vs WorldBank Data: Z = 0.007, p*-value=0.995; ResistanceMap vs WorldBank: Z=0.443, *p*-value=0.656), indicating that no single repository accounted for a disproportionate share of forecastable associations, and that the findings are not driven by any single data provider. Meteostat (n=6), Klein *et al*. (n=2) and Our World in Data (n=6) were excluded from the proportion Z-test due to their smaller number of indicators (n < 10), which precluded reliable statistical testing.

Despite these mitigation strategies, residual heterogeneity across countries and reporting systems remains unavoidable in global comparative analyses and should be considered when interpreting cross-country indicator associations.

Supplemental References

1. BV-BRC (2024). The Bacterial and Viral Bioinformatics Resource Center (BV-BRC). https://www.bv-brc.org/ Last accessed 01-09-24.
2. Natural Earth (2024). Natural Earth Data. https://www.naturalearthdata.com/ Last accessed 01-09-24.
3. World Bank Group (2025). World Bank country and lending groups FY26 (Last accessed 01-01-2026). https://datahelpdesk.worldbank.org/knowledgebase/articles/906519-world-bank-country-and-lending-groups.
4. Akorful, R.A.A., Odoom, A., Awere-Duodu, A., and Donkor, E.S. (2025). The Global Burden of Clostridioides difficile Infections, 2016-2024: A Systematic Review and Meta-Analysis. Infect Dis Rep *17*. https://doi.org/10.3390/idr17020031.
5. Boadu, A.A., Yeboah-Manu, M., Osei-Wusu, S., and Yeboah-Manu, D. (2024). Tuberculosis and diabetes mellitus: The complexity of the comorbid interactions. International Journal of Infectious Diseases *146*, 107140. https://doi.org/10.1016/j.ijid.2024.107140.
6. Kumar, B., Kaushal, I., Narayanan, B., and Narang, T. (2025). Sexually transmitted infections in the elderly: A growing concern in geriatric care. Indian J Sex Transm Dis AIDS *46*, 16-21. https://doi.org/10.4103/ijstd.ijstd_108_24.
7. Sarenje, K.L., Ngalamika, O., Maimbolwa, M.C., Siame, A., Munsaka, S.M., and Kwenda, G. (2022). Antimicrobial resistance of Neisseria gonorrhoeae isolated from patients attending sexually transmitted infection clinics in Urban Hospitals, Lusaka, Zambia. BMC Infect Dis *22*, 688. https://doi.org/10.1186/s12879-022-07674-y.
