# Supplementary material for: Genomic and socioeconomic drivers of antimicrobial resistance forecast to 2050
Source: Cell Genom. 2026 Jun 3;6(7):101273. doi: 10.1016/j.xgen.2026.101273 (PMC13347946; doi:10.1016/j.xgen.2026.101273)
Supplement: Document S2. Article plus supplemental information [file mmc20.pdf]

Cell Genomics

Article

Genomic and socioeconomic drivers of antimicrobial resistance forecast to 2050

Graphical abstract

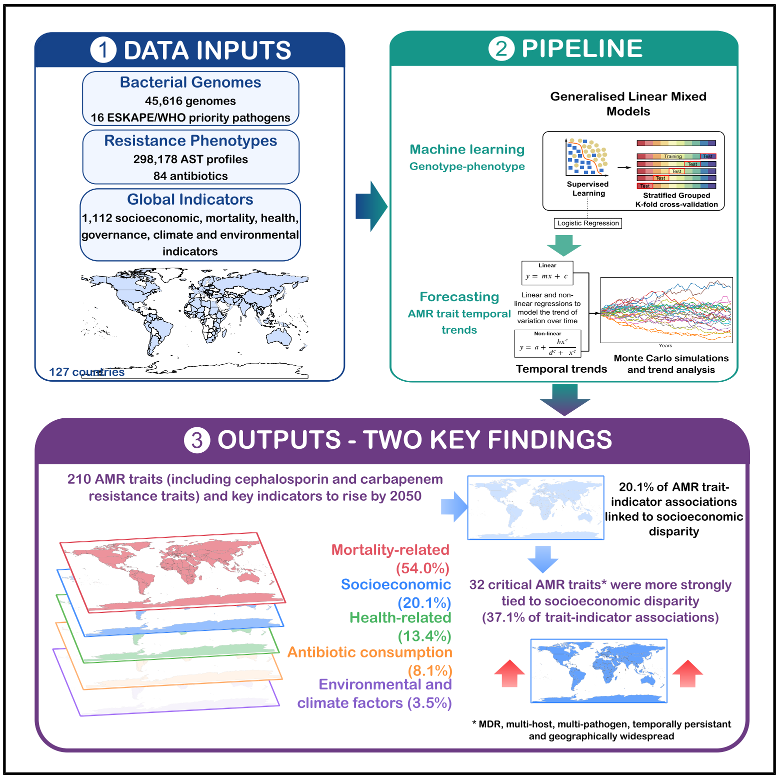

Highlights

- Forecasting models link rising AMR traits to socioeconomic and health data globally
- 210 pathogen-specific resistance traits across ESKAPE/WHO pathogens to rise by 2050
- Cephalosporin and carbapenem traits will increase in WHO critical pathogens
- 32 most critical rising traits strongly linked to socioeconomic disparity

Authors

Michelle Baker, Alexandre Maciel-Guerra, Ruoqi Wang, ..., Peter Anthony Oyom, Nicola Senin, Tania Dottorini

Correspondence

tania.dottorini@kcl.ac.uk

In brief

Antimicrobial resistance is an escalating global threat, yet its biological and social determinants remain poorly understood. Baker et al. used machine learning integrating global genomics, resistance phenotypes, socioeconomic variables, antibiotic consumption, climate data, and health data to forecast AMR traits to 2050. Critical rising resistance trends were strongly associated with socioeconomic inequality.

Baker et al., 2026, Cell Genomics 6, 101273
July 8, 2026 © 2026 The Author(s). Published by Elsevier Inc.
https://doi.org/10.1016/j.xgen.2026.101273

CellPress

Cell Genomics

CellPress
OPEN ACCESS

Article

Genomic and socioeconomic drivers of antimicrobial resistance forecast to 2050

Michelle Baker,[1,2] Alexandre Maciel-Guerra,[1,3] Ruoqi Wang,[3] Chengchang Luo,[3] Yan Xu,[3] Enzo Guerrero-Araya,[3] Weihua Meng,[3] Ge Wu,[3] Komkiew Pinpimai,[1,3] Peter Anthony Oyom,[1] Nicola Senin,[4] and Tania Dottorini[2,5,*]

[1]Faculty of Medicine and Health Sciences, Biodiscovery Institute, University of Nottingham, Nottingham NG7 2RD, UK

[2]Faculty of Life Sciences & Medicine, School of Immunology and Microbial Sciences, Department of Infectious Diseases, King's College London, London SE1 9RT, UK

[3]Centre for Smart Food Research, Nottingham Ningbo China Beacons of Excellence Research and Innovation Institute, University of Nottingham Ningbo China, Ningbo 315100, P.R. China

[4]Department of Engineering, University of Perugia, 06125 Perugia, Italy

[5]Lead contact

*Correspondence: tania.dottorini@kcl.ac.uk

https://doi.org/10.1016/j.xgen.2026.101273

SUMMARY

Antimicrobial resistance (AMR) is rising worldwide, and a better understanding of the genetic and socioeconomic determinants tied to it may establish a vantage point for surveillance and intervention. Unfortunately, the interactions between antibiotics, pathogens, and their environments are complex and deeply intertwined. Here, we present a novel machine learning and forecasting approach, integrating genomics, antibiotic phenotyping, and socioeconomic and environmental variables, designed to uncover hidden correlations and trends. Through the analysis of 45,616 bacterial genomes from 16 pathogens, 298,178 resistance profiles, and 1,112 social, economic, and environmental indicators collected across 127 countries, we identified 210 pathogen-specific AMR traits projected to increase by 2050, together with the key indicators associated with these trends. These traits were identified using structure-aware mixed-effects models with cluster-grouped cross-validation, controlling for lineage dependence. The 32 most critical rising traits were strongly linked to indicators of socioeconomic disparity. These findings provide a roadmap for targeted AMR interventions.

INTRODUCTION

Antimicrobial resistance (AMR) is among the most pressing public health threats globally,[1] with an estimated 10 million deaths per year by 2050.[2] While many governments have implemented new surveillance and prevention programs,[3] a reliable assessment of the current AMR situation and future trends is essential to ensure their effectiveness. Recent genomics applications have supported better AMR surveillance,[4] but the importance of developing novel frameworks to integrate genomic data into forecasting models has recently been highlighted as a necessary step toward making reliable predictions of future AMR trends.[5]

A recent study by Naghavi et al.,[1] building on earlier works,[6–8] produced a forecast of the global AMR burden to 2050, estimating the likely deaths and disability-adjusted life years (DALYs) attributable to AMR based on 22 different pathogens in 204 countries, using AMR rates and a range of indicators including mortality, antibiotic sales and usage, and hospital discharge data.

Despite these advancements, research on AMR remains fragmented, with no studies to date integrating social, economic, and environmental indicators with AMR genomic traits at a granular level to produce robust data-driven forecasts.

AMR is triggered by an intricate interplay between multidrug-resistant (MDR) traits, mobile genetic elements (MGEs), cross-species and multi-host transmission, and key social determinants of health, all of which are collectively correlated with current AMR and future trends. Consequently, a deep understanding of these interactions is crucial not only to understand these complex networks but also for building accurate forecasting models.

Hence, critical questions remain unanswered in relation to which health-related, socioeconomic, and environmental factors are associated with the dissemination of the genomic traits of AMR and influence their transmission within and across bacterial species, hosts (e.g., humans), and geographies. Perhaps the most important questions are which AMR traits (ARGs and/or MGEs carrying ARGs) linked to treatment failure will spread globally over the next 25 years, posing a global health risk, and what will be the main associations tied to these trends? And how can we forecast these trends to inform pre-emptive interventions?

Gaining insight into such complex networks of AMR trait-factor interactions requires a broad-range observational perspective encompassing bacterial genome makeup, AMR and MDR traits, MGEs, hosts, geographic distributions, phenotypic resistance data, and other diverse factors involving societies and

This is an open access article under the CC BY license (http://creativecommons.org/licenses/by/4.0/).

economies. However, the search for correlations hidden beneath such complex aggregates of multiscale, heterogeneous variables poses a significant challenge, and attempts to make use of such correlations to investigate AMR are scarce. This is not because of a lack of data, but because adequate methods for aggregating, homogenizing, and analyzing such big data are absent.

Our hypothesis is that by integrating machine learning (ML) and predictive modeling to analyze genomic (whole-genome sequencing), phenotyping, and social determinants of health data, we can bridge these gaps in knowledge.

In this study, we developed a novel ML method, coupled with genomics, phenotyping, and predictive modeling, to achieve three key objectives.

Firstly, we aimed to globally identify genomic traits, specifically ARGs and their associated MGEs, strongly associated with observed AMR phenotypes. To do this, we developed a data analysis pipeline based on ML to analyze a global dataset of bacterial genomes, antibiotic susceptibility testing (AST) data, and contextual indicators (see results and STAR Methods).

Secondly, we aimed to investigate which AMR traits, among those identified in the first part of the analysis, were projected to rise over the next 25 years, along with the main social, economic, and environmental indicators shaping these trends. Using Pearson correlation, we linked the prevalence of AMR traits (the number of resistant isolates carrying each AMR trait relative to the total population of resistant and susceptible isolates, irrespective of whether they carry the trait) to 1,112 indicators spanning the seven World Bank regions. Temporal trends were projected using linear and non-linear regression and Monte Carlo simulations for uncertainty and statistical trend analysis. AMR traits whose prevalence was projected to rise by 2050, along with their associated indicators expected to increase or remain stable, were further analyzed.

Thirdly, we aimed to identify which of the AMR traits projected to increase by 2050 may present the highest risk as a global health concern. This was achieved by ranking the AMR traits based on clinical relevance,[9] presence in ESKAPE[10] and WHO lists of critical/high-priority pathogens,[11] mobility, temporal persistence, and spread across hosts and geographies.

Our results demonstrated an intricate interplay between AMR and MDR traits, MGEs, bacterial species, hosts (humans, animals, and environments), and key social, economic, and environmental indicators, which are collectively linked to current and future AMR trends up to 2050. At the core of our results, 210 AMR traits were identified possessing the strongest correlation to AMR resistance, a demonstrated increase of prevalence globally over the next 25 years, and featuring strong correlation with several social, economic, and environmental indicators, with mortality being the strongest, followed by social determinants of health, including socioeconomic disparities and antibiotic consumption. Interestingly, while mortality is correlated with projection trends of most AMR traits, socioeconomic disparities emerge as the main associations for the 32 AMR traits identified as critical threats. We believe our work provides actionable insights into tackling AMR challenges.

RESULTS

Global pattern of 45,616 bacterial genomes and associated AST profiles

We analyzed genomes and associated resistance-susceptibility phenotypic profiles from the BV-BRC database[12] (Table S1), together with antibiotic consumption, socioeconomic information, mortality, health-related, and environmental indicators sourced from public repositories (STAR Methods; Table S2).

Only genomes and associated resistance profiles passing specific quality filters (Figure S1; STAR Methods) were considered; these comprised 16 different bacterial species, including six ESKAPE and nine WHO critical/high-priority pathogens[11] (*Acinetobacter baumannii*, *Clostridioides difficile*, *Corynebacterium diphtheriae*, *Enterococcus faecium*, *Escherichia coli*, *Klebsiella pneumoniae*, *Mycobacterium tuberculosis*, *Neisseria gonorrhoeae*, *Pseudomonas aeruginosa*, *Salmonella enterica*, *Shigella flexneri*, *Shigella sonnei*, *Staphylococcus aureus*, *Streptococcus agalactiae*, *Streptococcus pneumoniae*, and *Streptococcus suis*). Genomes spanned 127 countries (Figure 1A), with the number of countries varying by bacterial species (between 1 and 57 countries per bacterial species) and isolate counts per country (range: 1–9,915). Resistance distribution across antibiotic classes also exhibited significant geographical and bacterial variation (Figures S2–S17). Comparison of the number of genomes across income groups (Tables S3 and S4; Figure S18) with pathogen prevalence rates collated under the WHO GLASS program[13] yielded only weak/moderate correlations between pathogen prevalence and sequence availability (Spearman's ρ ranging from -0.19 to 0.34). The negative correlations for some pathogen-syndrome combinations (e.g., *E. coli* BSI [bloodstream infection] and *K. pneumoniae* BSI by income group) likely reflect an inverse relationship between disease burden and sequencing capacity. High-income countries (HICs) tend to have lower BSI rates caused by these pathogens.

Standard bioinformatics pipelines were used to identify all ARGs and ARG-carrying MGEs and their locations, categorized as ARGs-Chr (ARGs located on the bacterial chromosome itself), ISs-ARGs (ARGs that are integrated into the chromosome near insertion sequences), ISs-plasmid-ARGs (ARGs present near insertion sequences on plasmids), or plasmid-ARGs (ARGs located on plasmids but not within an insertion sequence) (STAR Methods), allowing for post hoc analysis of different mechanisms of transfer and spread of resistance based on their location (STAR Methods). The AMR trait locations varied across species (Figures 1A and S19; Table S5). Identified AMR traits spanned a wide range of contig lengths (interquartile range [IQR]: 3.8–197 kbp) with a median contig length of 72 kbp. In *S. enterica*, *K. pneumoniae*, and *S. aureus*, over 79% of traits were located in mobile regions, either as ISs-plasmid-ARGs, plasmid-ARGs, or ISs-ARGs. *S. agalactiae* and *C. difficile* did not possess any plasmid-located traits, with over 90% of their traits classified as ARGs-Chr, while the remaining traits were ISs-ARGs. Similarly, *C. diphtheriae* contained mostly chromosomal ARGs (ARGs-Chr and ISs-ARGs), with some occurring in both forms. *A. baumannii*, *E. faecium*, *K. pneumoniae*, and *S. enterica* exhibited great diversity in ARG locations within the same isolate, with certain ARGs found in multiple locations and

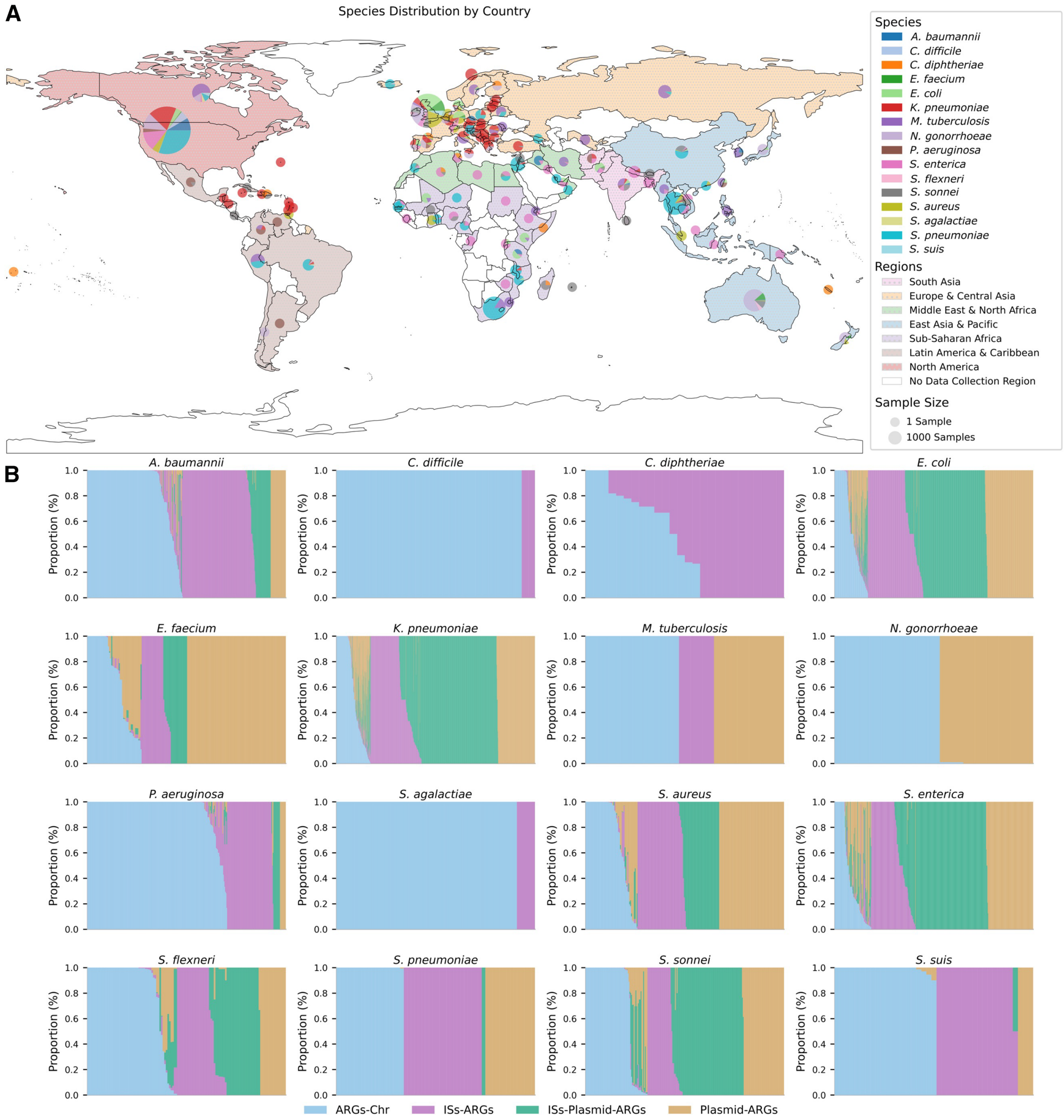

Figure 1. Geographic distribution of bacterial isolates and AMR genomic traits

(A) Geographic distribution of the 45,616 genomes (16 bacterial species) and 298,178 associated resistance phenotypic profiles. Pie charts show the proportion of data per species in each country; chart size indicates total genome counts. The world map[14] is divided into the seven World Bank regions.

(B) Distribution of AMR trait locations across all isolates of each bacterial species. Stacked bars indicate the proportion of isolates with each trait carried as ARGs-Chr (light blue), ISs-ARGs (pink), ISs-plasmid-ARGs (green), and plasmid-ARGs (orange).

configurations (Table S6). For example, in *K. pneumoniae* isolate 1328414.3, *aadA2* occurred chromosomally as ISs-ARGs within 5 kbp[15,16] of IS*26* and IS*6100* (co-localized with *qacEdelta1* and *sul1*). In addition, it was found on a plasmid of replicon type IncFIB/IncFII/rep_cluster_2183, within 5 kbp of IS*26* and IS*6100* carrying *qacEdelta1*, *sul1*, *mrx*, *dfrA12*, and *mphA* as an ISs-plasmid-ARG. Furthermore, within the same isolate, *aadA2* was also found on a second plasmid of replicon type IncR, located within 5 kbp of IS*Kpn26*, which carried *aadA2*, *aadA*, *cmlA1*, and *sul3*. These co-occurrences suggest possible inter-plasmid or plasmid-chromosome transfer.

ML identifies 1,797 unique AMR traits that predict resistance

To determine which genomic traits, herein collectively termed genomic features (short for AMR genomic features), correlate with the observed AST profiles, we developed an ML pipeline adapted from previous results[15–20] (see STAR Methods and Figure 2; see details in Figures S20–S22). Random effects accounted for population structure, geography, year, and host. Interclass correlation coefficients (mean ICCs: population structure: 0.16, geography: 0.23, year: 0.09, and host: 0.12) indicated that these factors contributed non-negligible variance (Table S7).

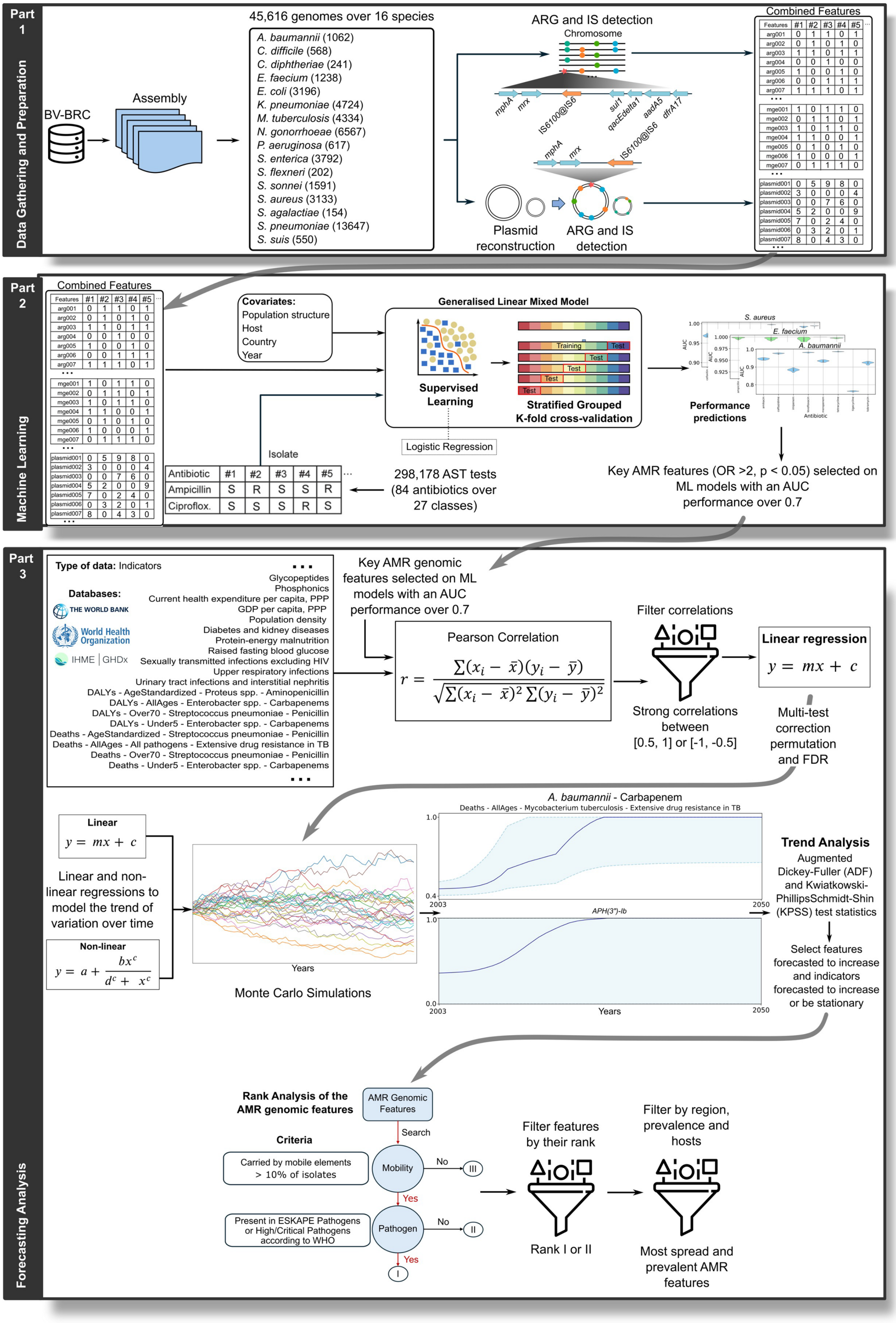

Figure 2. Overview of the data analysis pipeline

Schematic of the three-stage pipeline: bioinformatic feature identification, machine learning prediction of resistance, and forecasting analysis of feature prevalence and ranking by clinical risk. Detailed workflows are provided in Figures S20–S22.

CellPress
OPEN ACCESS

Of the 16 species in the study, 14 bacterial species had at least one predictor model achieving an acceptable[21] area under the curve (AUC) > 0.7 and were retained for downstream analysis (Table S7). Among these, 13 species had at least one model with an AUC > 0.8, and 11 species had at least one model with an AUC > 0.9.[22] Feature-pathogen pairs were retained based on effect size, statistical significance, and cross-validation stability criteria (STAR Methods); the resulting features and odds ratios are detailed in Table S8. Two species (*M. tuberculosis* and *C. difficile*) had no antibiotic models with an acceptable AUC (>0.7), and *N. gonorrhoeae* lacked statistically significant features (Figure S23). Nevertheless, given the global public health importance of these pathogens, a forecasting analysis of all AMR genomic features within these species was conducted. Ten feature-pathogen pairs in these species were forecast to increase over time; however, it is important to note that these genomic AMR features could not be robustly linked to phenotypic resistance and thus may not be representative of clinical resistance (Data S1; Table S9).

In total, 2,470 pathogen-specific genomic features (hereafter feature-pathogen pairs) were selected across 14 species. Collectively, these corresponded to 1,797 unique features (Table S10). Of these, 252 were ARGs, 312 ISs-ARGs, 417 ISs-plasmid-ARGs, 647 plasmid-ARGs, and 842 found in mixed locations.

The number of selected features per model ranged from 1 to 207 (median = 53). Gram-negative species had significantly more selected features ($n = 73$) than Gram-positive species ($n = 13.5$; $p = 5.102 \times 10^{-12}$, Mann-Whitney, two-tailed), a difference that persisted after explicit modeling of lineage structure within the mixed-effects framework and may reflect a greater tendency for Gram-negative strains to evolve resistance, though it could also be influenced by biases in AMR gene representation in databases.

Across all 14 species, 353 features were shared between at least two species: 7 ARGs-Chr, 95 plasmid-ARGs, 12 ISs-plasmid-ARGs, 3 ISs-ARGs, and 236 in mixed locations. A clear Gram stain distinction was observed: 320 were exclusive to Gram-negative species, 19 to Gram-positive species, and 14 in both. Among the 14 features shared between Gram-negative and Gram-positive species, eight ARGs (*aph(3″)-Ib*, *aph(3′)-Ia*, *aph(6)-Id*, *dfrA1*, *dfrA16*, *qacEdelta1*, *sul1*, and *aadA*) were selected exclusively between some Gram-negative species and the Gram-positive *C. diphtheriae*, where these genes have also previously been reported.[23,24] The ARG *lnuA* was detected only in *S. aureus* and *S. enterica*, with closely related variants (e.g., *linG*) previously reported in both species.[24,25] An *S. aureus*-origin plasmid (rep_cluster_1017/rep_cluster_1733/rep_cluster_2100) was detected in both *S. aureus* and *A. baumannii*, although interspecies transfer has not yet been demonstrated experimentally. Three tetracycline resistance genes (*tetW*, *tetM*, and *tetS*) with broad host range were shared across multiple Gram-positive and Gram-negative species. The most widely shared feature, selected by both Gram-negative and Gram-positive bacterial species, was the ARG *ermB*, an MDR gene known to confer resistance to streptogramins, lincosamides, and macrolides. The macrolide resistance gene *ermB* was identified as a critical predictor in models across nine bacterial species spanning both Gram-positive and Gram-negative groups. Analysis of its genomic contexts (Table S11) showed that although *ermB* is frequently associated with shared MGEs within Gram-positive and Gram-negative species, no identical MGEs were observed between the two groups, and in Gram-positive species, *ermB* was mostly carried chromosomally. These findings indicate that while *ermB* is widely disseminated across taxonomic classes, its mobility is facilitated by distinct, lineage-compatible vectors rather than a single conserved cross-group element. Interestingly, the second-most-shared ARG selected in ML models across 8 bacterial species, including *C. diptheriae*, was *qacEdelta1*, a gene conferring resistance to biocides. This finding raises concerns about the potential co-evolution of antibiotic and biocide resistance.

Furthermore, when considering the antibiotics grouped into classes, among the 2,470 feature-pathogen pairs uncovered by the ML analysis, 663 pairs were found in critically important antibiotic classes (626 linked to cephem, 19 to colistin, and 119 to carbapenem, including 102 linked to multiple classes), particularly against Gram-negative bacterial species (see Table S12).

Health, mortality, environmental, and socioeconomic indicators predict AMR features that pose future global health risks

Of the ML-selected AMR feature-pathogen combinations across the 14 species passing the ML selection, 1,416 showed strong and significant correlations ($\rho > | 0.5 |$, $p < 0.05$), with a median of 13 indicators per feature (range: 1–632) (Table S13). The ten most frequently correlated indicators included eight linked to morbidity and mortality indicators, one poverty-related indicator, and one environmental indicator.

Reconstructing historical trends and forecasts of feature prevalence (joint burden prevalence, STAR Methods) and strongly correlated indicators identified 304 genomic feature-pathogen pairs with increasing prevalence over time. These comprised 34 ARGs-Chr, 54 Plasmid-ARGs, 7 ISs-plasmid-ARGs, 19 ISs-ARGs, and 199 ARGs with mixed locations. Of these, 257 were associated with rising indicator trends, 62 with stationary, and 189 with decreasing indicators, with some feature-pathogen pairs linked to multiple indicators (Tables S14, S15, and S16). Decreasing-indicator-pair trends were excluded from further analysis, leaving a total of 280 increasing or stationary feature-pathogen pairs.

Sensitivity analysis using marginal and conditional prevalence (STAR Methods) yielded substantial concordance (75% and 71% overlap of feature-pathogen pairs identified by the primary metric were also identified using the marginal and conditional definitions, respectively, with Spearman's rank coefficients of 0.886 for the marginal definition and 0.82 for the conditional definition (Tables S17 and S18).

The principal conclusions of the study are supported under all prevalence definitions, with each highlighting complementary aspects of AMR dynamics.

Of the 280 AMR feature-pathogen pairs predicted to increase or remain stationary, 58.6% were MDR, 83.5% were found in two or more World Bank geographic regions, 65.7% were present in more than one species, and 92.5% persisted across multiple years (Figure 3). Co-occurrence of multidrug resistance,

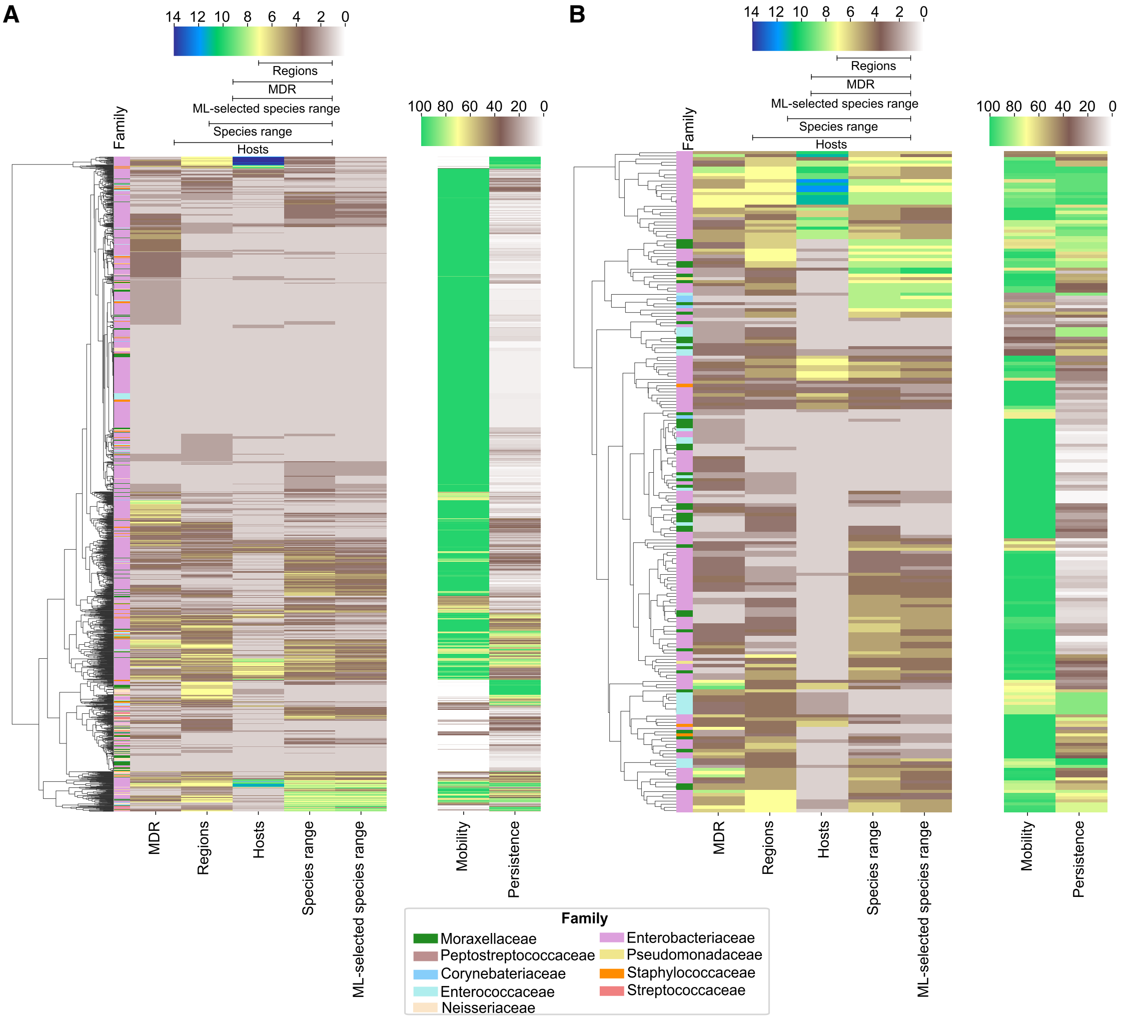

Figure 3. Metadata associated with ML-identified AMR genomic features

Clustered heatmaps illustrating AMR genomic features in feature-pathogen pairs identified by ML (A) and the subset predicted to increase in prevalence, linked to rising or stable indicators (B). Rows show feature-pathogen pairs grouped by family-level taxonomy; columns indicate MDR status, geographic spread (World Bank region), host range, species range, ML-selected species range, mobility, and temporal persistence.

geographical spread, and lateral transmission was higher in the 280 feature-pathogen pairs than in the overall ML-selected resistance-based feature set, indicating a concerning link between increasing AMR prevalence, indicator trends, and AMR-related genomics.

High-risk AMR features projected to rise by 2050

To prioritize the features posing the greatest threat to human health, we ranked the 280 increasing or stationary features based on clinical significance[9] (STAR Methods; Figures 2, part 3, and S22).

The classification resulted in 98 rank I, 112 rank II, and 70 rank III feature-pathogen pairs, encompassing 210 unique features. Twenty-four features appeared in multiple feature-pathogen pairs with varying rankings, reflecting differences in mobility properties across species. Only rank I and II features were retained for further analysis.

The 210 rank I/II feature-pathogen pairs (encompassing 157 unique features) spanned nine species, including ESKAPE[10] (*A. baumannii*, *E. faecium*, *K. pneumoniae*, and *S. aureus*) and WHO critical/high-priority pathogens[11] (carbapenem-resistant *A. baumannii*, 3rd-generation cephalosporin-resistant *E. coli*, fluoroquinolone-resistant *S. enterica*, vancomycin-resistant *Enterococcus faecium*, and fluoroquinolone-resistant *S. sonnei*). These pairs exhibited a notable enrichment of critical risk factors: 63.3% exhibited multidrug resistance, 33.8% were multi-host, 47.6% were persistent across years (i.e., present in $\geq$30% of years with available genomes), and 81.0% were found across multiple world regions (Figure 4; Table S19). Notably, within the five species in which *ermB* was forecastable, it was classified as rank I/II in Gram-negative pathogens, where it was predominantly carried as a plasmid-ARG feature. In contrast, among Gram-positive pathogens, it ranked III because it was carried almost exclusively as a non-mobile

CellPress
OPEN ACCESS

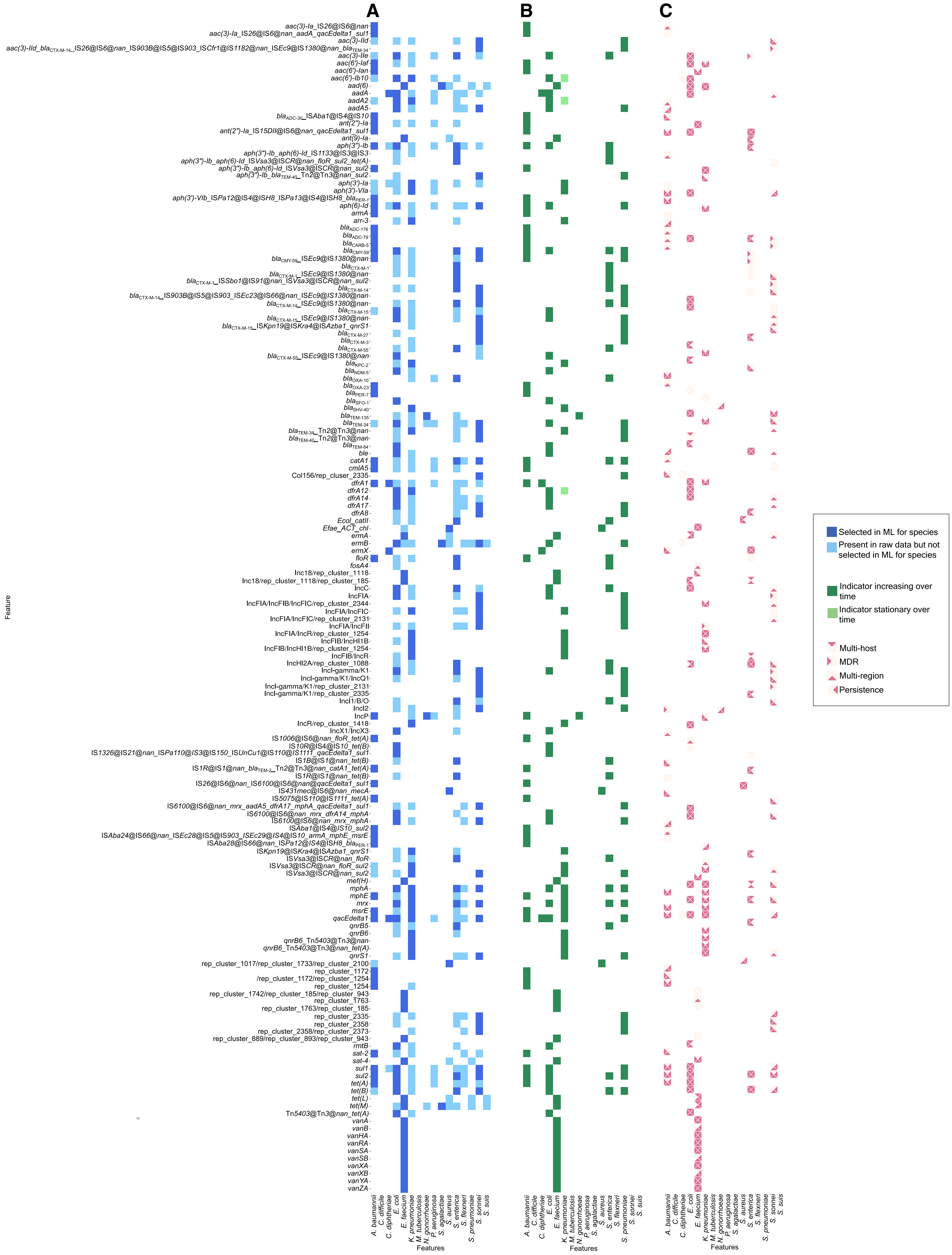

(legend on next page)

ARGs-Chr feature (Figure S24). In both Gram-negative and Gram-positive species, it was associated primarily with mortality-related indicators. Folate (47.1%, $n = 99$) and cephem (46.6%, $n = 98$) resistance were the most frequently associated resistance classes among the identified rank I/II features, followed by aminoglycosides (45.2%, $n = 95$) and penicillin (41.0%, $n = 86$) resistance. Genes such as *bla*$_{\text{CMY-59}}$ and *bla*$_{\text{CTX-M-1}}$ in *S. enterica* and *bla*$_{NDM\text{-}5}$ in *E. coli* and *bla*$_{\text{KPC-2}}$ in *K. pneumoniae*, important for cephem and carbapenem resistance, were also uncovered by the forecasting analysis.

Among the 210 feature-pathogen pairs, 32 unique features (*aac(3)-IIe*, *aac(6′)-Ib10*, *aadA*, *aadA2*, *aadA5*, *aph(3″)-Ib*, *aph(3′)-Ia*, *aph(6)-Id*, *catA1*, *bla*$_{\text{CMY-59}}$, *bla*$_{\text{CTX-M-15}}$, *bla*$_{CTX\text{-}M\text{-}15}$_ISEc9, *dfrA12*, *dfrA14*, *dfrA17*, IncFIA, IncFIB/IncHI1B, IncHI2A/rep_cluster_1088, IncI-gamma/K1, IncX1/IncX3, IS*6100_mrx_dfrA14_mphA*, IS*6100_mrx_mphA*, *mphA*, *mrx*, *qacEdelta1*, *qnrS1*, *sul1*, *sul2*, *bla*$_{\text{TEM-34}}$, *tet(A)*, *tet(B)*, and Tn*5403_tet(A)*) across 38 feature-pathogen pairs were classified as critical threats (STAR Methods).

Network analysis of rank I/II genomic features, locations (ARGs-Chr, ISs-ARGs, ISs-plasmid-ARGs, and plasmid-ARGs), indicators, and species (Figures 5, 6, and 7) showed that 64% of pairs associated with only one indicator type (health-related, socioeconomic, mortality, environmental, or antibiotic consumption) and 36% with multiple indicator groups: one was connected to five groups, 15 were connected to four, 33 pairs to three, and 27 pairs to two (Figure 5A). Among those associated with five or four indicators, 11 features (*aadA5*, *catA1*, *dfrA17*, *mphA*, *mrx*, *qacEdelta1*, *sul1*, *sul2*, bla$_{\text{TEM-34}}$, *tet(A)*, and *tet(B)*) were among the 32 most critical threats and were associated with mortality, environmental, and socioeconomic indicators. Additionally, *bla*$_{\text{TEM-34}}$ was also connected to antibiotic consumption. Comparisons across bacterial species revealed significant differences in the number of indicators associated with each feature (Kruskal-Wallis, two-tailed, $p = 1.962 \times 10^{-12}$; Figure S25A). When analyzed by individual indicator type, all categories showed significant species-specific differences (Kruskal-Wallis, two-tailed, adjusted $p \leq 0.0001$; Figure S25B). Overall, *S. sonnei* was more strongly correlated with environmental indicators, while *E. coli* and *S. enterica* were more strongly associated with antibiotic consumption indicators than other bacterial species. IS*6100_mrx_aadA5_dfr17_mphA_qacEdelta1_sul1* was associated with mortality, health-related, and environmental indicators, suggesting a correlation with clinical settings. Interestingly, this feature also showed high synteny sequence conservation across the four pathogens, most likely suggesting cross-species transmission (Figure 5B).

Genomic location, feature type, resistance profiles, and bacterial species of rank I/II features

Only two rank I/II features were shared between Gram-positive and Gram-negative bacteria, namely *aadA* and *qacEdelta1* (Figure 6A). Gram-positive species (*C. diphtheriae*, *E. faecium*, and *S. aureus*) exhibited minimal feature sharing, even among themselves, whereas Gram-negative species displayed a high degree of shared features (*S. sonnei*, *S. enterica*, *E. coli*, *K. pneumoniae*, *P. aeruginosa*, and *A. baumannii*).

S. aureus and *S. enterica* were associated exclusively with features linked to increasing indicators. *C. diphtheriae*, *E. faecium*, *E. coli*, *S. sonnei*, *K. pneumoniae*, and *A. baumannii* had features associated with both increasing and stationary indicators.

Species-specific differences were observed in the location of AMR features and MDR. The genomic location of features varied across bacterial species, but *C. diphtheriae*, *S. enterica*, *E. coli*, *K. pneumoniae*, and *S. aureus* exhibited no significant difference (Kruskal-Wallis, two-tailed, Dunn's adjusted $p > 0.05$), with many of their features being plasmid-ARGs. *S. sonnei* showed a similar distribution across all genome locations except ISs-ARGs, having significantly fewer compared to *E. coli*, *A. baumannii*, and *C. diphtheriae* (Kruskal-Wallis, two-tailed, Dunn's adjusted $p < 0.05$). *A. baumannii* carried significantly fewer features as plasmid-ARGs compared to *E. coli*, *S. sonnei*, and *E. faecium* but significantly more ISs-ARGs compared to *E. coli*, *E. faecium*, *K. pneumoniae*, *S. enterica*, and *S. sonnei* (Kruskal-Wallis, two-tailed, Dunn's adjusted $p < 0.05$).

Among the 32 AMR features identified as critical threats, all ARGs had both a mobile and non-mobile genomic configurations and generally appeared in similar proportions among the selected bacterial species. The gene *aph(3″)-Ib* was an exception, predominantly found as ISs-ARGs in *A. baumannii* (86.4%), whereas in *E. coli* and *S. enterica*, it was primarily detected as plasmid-ARGs (63.8% and 69.2%, respectively).

As expected, due to intrinsic resistance and bacterial species-specific sensitivities, resistance classes varied among features within each bacterial species (Figure 6B). While the median number of resistance classes in *E. coli* and *K. pneumoniae* features was higher (median = 6 and 5, respectively) than other pathogens, a direct comparison of resistance features across all bacterial species is not possible due to data gaps, which result in a varying number of antibiotics and classes trained in the ML models (range: 1–16 antibiotics and 1–9 classes). However, when normalized by feature-pathogen pair representativeness across all the ML models trained per species (Figure S26), we observe that *E. coli*, *K. pneumoniae*, and *S. enterica* have higher representativeness than *S. sonnei* (Kruskal-Wallis, two-tailed, Dunn's adjusted $p = 0.0138$, 0.0001, and 0.0001, respectively), but all other species are not statistically different.

Host and geographic distribution of rank I/II features

Among the 157 unique features, 40% (63/157) of them were detected in at least one host beyond humans (Figure 7A). A significantly greater proportion of rank I/II features were shared between humans and food animals—including pigs, chickens, sheep, and birds—compared to all ML-selected features (two-tailed z test, $p = 0.00004$). However, 28.6% of rank I/II features

Figure 4. Heatmaps of the 157 unique rank I/II features (from 210 feature-pathogen pairs)
(A) ML feature selection by species: dark blue, the feature was present on the pathogen and selected, and light blue, the feature was present but not selected.
(B) Indicator trends associated with feature forecast to increase by 2050: dark green, increasing, and light green, stationary.
(C) Critical risk factors linked to AMR genomic features associated with increasing or stationary indicators.

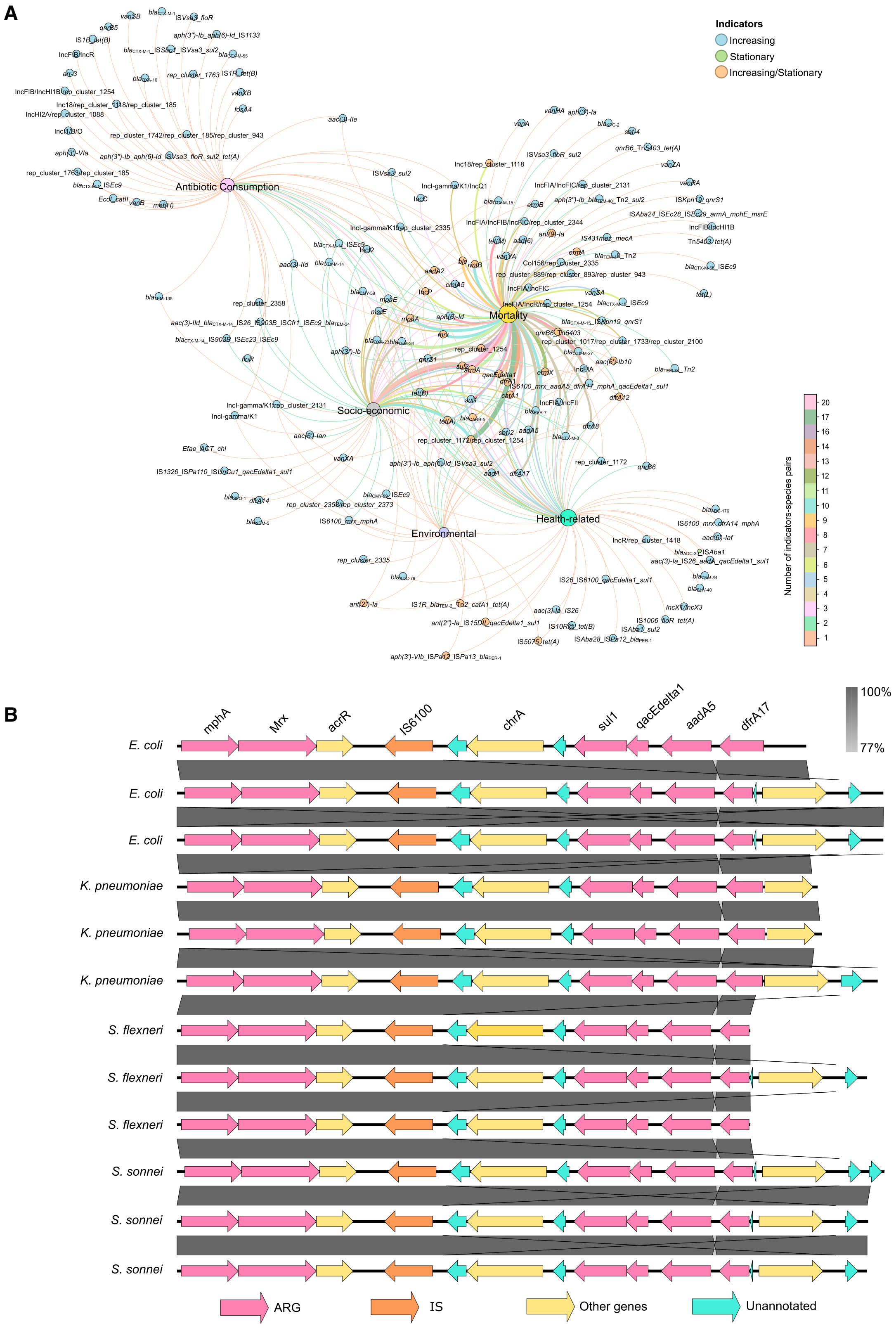

(legend on next page)

were also observed in non-food animals (camels, cats, dogs, horses, insects, mollusks, penguins, and rats).

Among the 32 critical-threat features, 11 (*aac(3)-IIe*, *aph(3″)-Ib*, bla_{TEM-34}, *aadA*, *mphA*, *mrx*, *qacEdelta1*, *sul1*, *sul2*, *tet(B)*, and *tet(A)*) were the most extensively shared across hosts, each being present in 10 or more different hosts (visible as a cluster in Figure 7A). The remaining 22 features were predominantly shared between humans and food animals.

The selected rank I/II features also demonstrated widespread geographic distribution. 79% were found in multiple (2–7) World Bank regions, and 21 (14%) were detected in all seven World Bank regions (Figure 7B). Among the 32 critical-threat features, nineteen (*aac(6′)-Ib10*, *aadA2*, *aph(3″)-Ib*, *aph(6)-Id*, *sul2*, *aadA5*, *aph(3′)-Ia*, $bla_{CTX-M-15}$, *catA1*, *dfrA12*, *dfrA17*, *mphA*, *mrx*, *qacEdelta1*, *qnrS1*, *sul1*, *tet(A)*, IncFIA, and IncHI2A/rep_cluster_1088) were present in all seven World Bank regions. Three features (*aadA*, bla_{TEM-34}, and *tet(B)*) were found across six regions, being absent only from the Middle East and North Africa. Seven features (IncI-gamma/K1, *aac(3)-IIe*, bla_{CMY-59}, $bla_{CTX-M-15}$_IS*Ec9*, *dfrA14*, IncFIB/IncHI1B, and Tn*5403_tet(A)*) were detected in five World Bank regions, while three features (IncX1/IncX3, IS*6100_mrx_dfrA14_mphA*, and IS*6100_mrx_mphA*) were found in four regions.

Forecasting trends of genomic AMR evolution: Species-specific predictors

Most forecastable indicators were mortality-related (54.0%), followed by socioeconomic (20.1%), health-related (13.4%), antibiotic consumption (8.1%), and environmental factors (3.5%). Mortality and health-related indicators were widely associated with *A. baumannii*, *S. sonnei*, *E. coli*, *K. pneumoniae*, and *C. diphtheriae*, with mortality but not health-related indicators also associated with *E. faecium*. Antibiotic consumption indicators were mostly associated with *S. sonnei* and *S. enterica*. Environmental indicators were primarily associated with *S. sonnei* features, though a small number were also associated with *K. pneumoniae* and *A. baumannii* features, while socioeconomic indicators were associated with *A. baumannii*, *E. coli*, and *S. sonnei* features. The most frequently forecast socioeconomic indicator was population density, associated with an increased prevalence of *A. baumannii*, *E. coli*, and *S. sonnei* features. Health expenditure was associated with increased prevalence of genomic features in *A. baumannii*, *E. coli*, *E. faecium*, *S. sonnei*, and *S. aureus*.

Seventy-six indicators were associated with 21 geographically spread features (*aac(6′)-Ib10*, *aadA2*, *aadA5*, *aph(3′)-Ia*, *aph(3″)-Ib*, *aph(6)-Id*, *dfrA12*, IncFIB/IncFII, *mphA*, *mrx*, *qacEdelta1*, *qnrB6*, rep_cluster_2335, *sul1*, *sul2*, and *tet(A)*); these included livestock production index, GDP, current health expenditure, and population density. Other indicators were more regionally specific, found in no more than three World Bank regions, including group A and B *Streptococcus* death and DALYs and poverty headcount.

Association with mortality indicators also varied by pathogen. Those related to *Enterobacter* spp. deaths and DALYs were identified in between four and seven regions (median = 5), whereas indicators associated with group A *Streptococcus* death and DALYs were found in no more than five regions (median = 4).

Eight bacterial species (*A. baumannii*, *E. faecium*, *C. diphtheriae*, *E. coli*, *K. pneumoniae*, *N. gonorrhoeae*, *S. sonnei*, and *S. aureus*) had species-specific indicators (discussed below), with *A. baumannii*, *K. pneumoniae*, and *S. sonnei* linked to the highest number (n = 53, 31, and 25, respectively; Tables S14, S15, and S16).

Some forecast AMR features were found to co-occur frequently, in particular *sul1*, *sul2*, *mphA*, *ermB*, and *qacEdelta1*, most likely due to co-localization on plasmids. Significant co-occurrence was observed only for *sul2* with a subset of other genes in *K. pneumoniae*, *S. sonnei*, and *S. flexneri* (adjusted $p \leq 0.05$); no other gene pairs showed significant associations.

We then evaluated whether these co-occurrences biased the indicator associations. In *S. flexneri*, no forecast indicators were selected. In *K. pneumoniae*, no overlap was observed between the indicators associated with rank I/II and *sul2* and *mphA*, *ermB*, *sul1*, or *qacEdelta1*. However, in *S. sonnei*, seven of the 26 indicators were shared between *sul2* and at least one of *mphA*, *ermB*, *sul1*, or *qacEdelta1*. These seven indicators were population, population density, livestock production index, and all-cause deaths (all ages) attributable to *E. coli* resistance to β-lactam/β-lactamase inhibitor combinations, *E. faecium* resistance to ≥1 antibiotic, *E. faecium* resistance to fluoroquinolones, and vancomycin-resistant infections across all pathogens. This suggests that, for these seven indicators in *S. sonnei*, the forecast signals may not be fully independent and could be partly driven by gene co-occurrence.

K. pneumoniae

In *K. pneumoniae*, 27 rank I/II features were associated with increasing and stationary indicators, all but three of which were selected by at least three ML drug class models (Figure S27). We identified 31 increasing or stationary indicators, spanning mortality, antibiotic consumption, health-related, and environmental factors. Consumption of fluoroquinolones was related to increased prevalence of eight genomic features, including 3 ARG-carrying plasmids and *qnrS1*. Additionally, the number of deaths related to fluoroquinolone-resistant *K. pneumoniae* infections was a strong predictor of increasing prevalence of the fluoroquinolone resistance feature *qnrB6*_Tn*5403*. Furthermore, indicators of diabetes, kidney disease, urinary tract infections (UTIs), and intestinal nephritis predicted the increasing prevalence of *qnrB6* and *qnrB6*_Tn*5403*. Meanwhile, six features, including *aac(6′)-Ib10*, *dfrA12*, and IncFIB/IncHI1B, were linked to upper respiratory infections (e.g., measles and *Haemophilus influenzae*), suggesting a broader correlation between AMR genomic features and specific health conditions.

Figure 5. Undirected network of rank I/II genomic features and associated indicator groups

(A) Feature (nodes colored by indicator trend: blue, increasing; green, stationary; and orange, both) connected to indicator-group-type nodes: mortality, health-related, antibiotic consumption, socioeconomic, and environmental; node size is proportional to the number of indicators.

(B) Synteny of IS*6100_mrx_aadA5_dfr17_mphA_qacEdelta1_sul1* feature across three isolates each of *E. coli*, *K. pneumoniae*, *S. flexneri*, and *S. sonnei*. Genes are colored by function: ARG, pink; IS, orange; other functions, yellow; and unannotated, cyan).

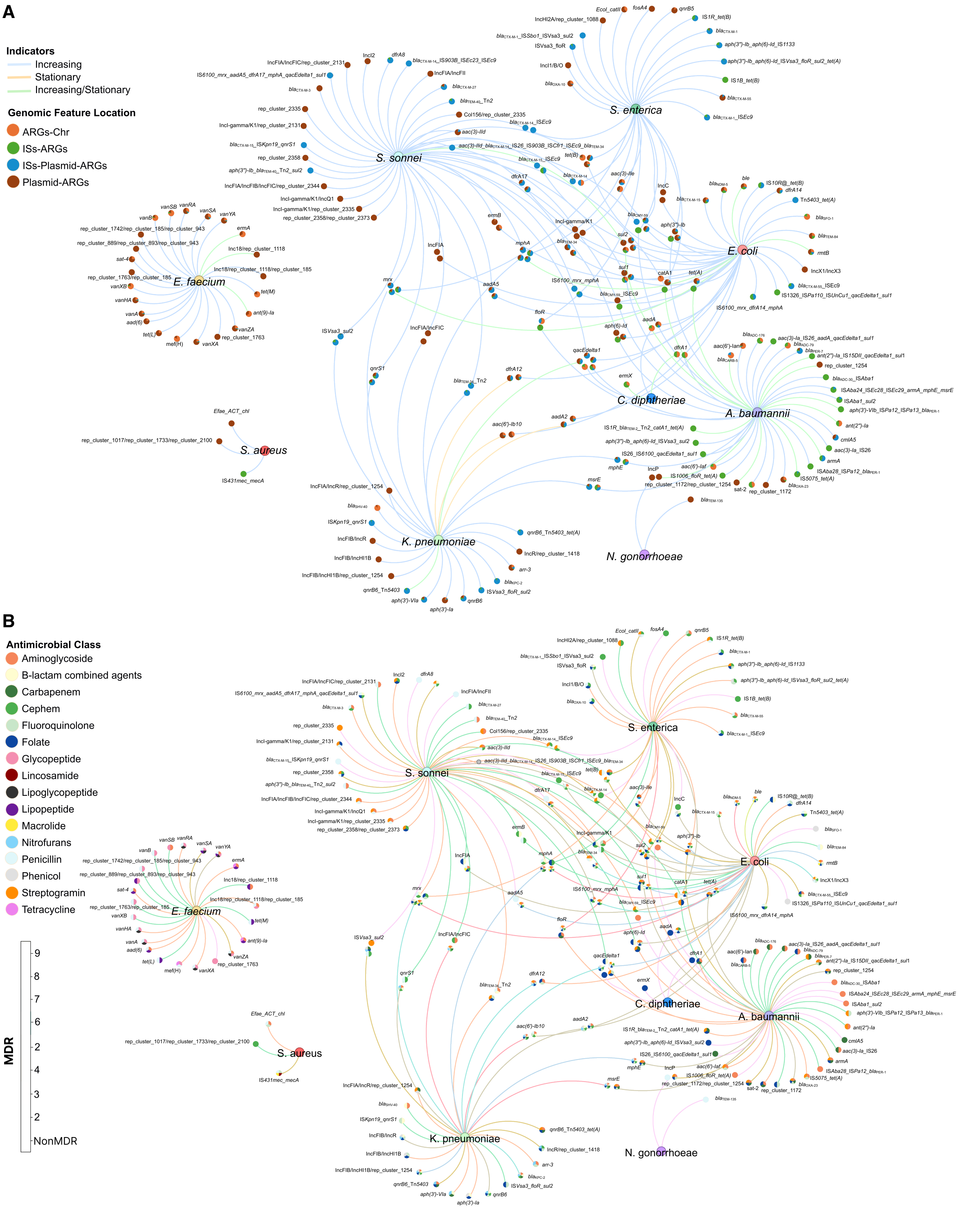

(legend on next page)

A. baumannii

In *A. baumannii*, 41 rank I/II features were associated with 50 increasing and five stationary indicators (Figure S28). These indicators spanned six antibiotic drug classes: carbapenems, cephems, folate, tetracyclines, and fluoroquinolones for increasing and stationary indicators and aminoglycosides for stationary indicators only. The prevalence of plasmids rep_cluster_1172/rep_cluster_1254 and rep_cluster_1254, which harbor multiple resistance genes and ISs, was forecast to increase based on 32 overlapping indicators, including mortality related to pneumoconiosis, aminopenicillin-resistant *Proteus* spp. infection, extensively drug-resistant *M. tuberculosis*, and death-related extensively drug-resistant tuberculosis (XDR-TB). These plasmid features were projected to follow an increasing trend across three drug classes identified from ML: carbapenems, tetracyclines, and folate. Furthermore, mortality indicators related to XDR-TB were predicted to rise through 2050 and were positively correlated with 19 other resistance features: *aph(3″)-Ib*, *aph(6)-Id*, *sul2*, *armA*, bla_{OXA-23}, bla_{CARB-5}, *msrE*, *mphE*, IncP, and *tet(A)*.

S. sonnei

S. sonnei carried 45 selected rank I/II features selected in ML by five antibiotic drug classes: cephems, folates, penicillins, tetracyclines, and aminoglycosides, which were predicted to increase in prevalence alongside two stationary and 25 rising indicators (Figure S29). Among these, folates, penicillins, and tetracyclines were the most frequently associated with the selected features.

The correlated indicators included multiple mortality indicators, as well as health-related factors (such as tetanus, typhoid fever, obesity, and protein-energy malnutrition), socioeconomic indicators (such as GDP and people using safely managed sanitation) and environmental (e.g., livestock production index) and antibiotic consumption trends (carbapenems and macrolides).

E. coli

E. coli carried a total of 37 rank I/II features, which were linked to 22 increasing/stationary indicators associated with nine antibiotic drug classes by the ML models: penicillin, macrolide, phenicol, cephems, β-lactam-combined agents, fluoroquinolones, folate, tetracyclines, and aminoglycosides. Aminoglycosides and fluoroquinolones were the most frequently associated with these features (Figure S30).

Fourteen of the associated indicators were mortality-related, five socioeconomic, and two health-related, and one was an antibiotic consumption indicator, with access to basic handwashing facilities followed by population density being the most common predictors, forecasting an increase in the prevalence of 21 and 10 features, respectively.

E. faecium

In *E. faecium*, 23 rank I/II features selected by two different antibiotic classes (glycopeptides [15 features] and lipopeptides [10 features]) were predicted to increase in prevalence and were associated with 18 rising or stationary indicators. Fifteen of these indicators were mortality indicators, one was socioeconomic (current health expenditure), and one was antibiotic consumption (phosphonic acid).

Notably, eight rank I/II features (*vanB*, *mef(H)*, *vanXB*, *vanSB*, Inc18/rep_cluster_1118/rep_cluster_185, rep_cluster_1742/rep_cluster_185/rep_cluster_943, rep_cluster_1763, and rep_cluster_1763/rep_cluster_185) associated with glycopeptide resistance by the ML models were linked to phosphonic acid antibiotic consumption (Figure S31). Three of these features (*vanB*, *vanXB*, and *vanSB*) are well-known for their association with vancomycin resistance, and all four plasmid types also carried *van* genes.

S. enterica

In *S. enterica*, 27 rank I/II features were identified as correlated with resistance to cephem (26 features) and tetracycline (1 feature). The 26 cephem-associated features were linked to an increase in phosphonic acid antibiotic consumption, whereas the tetracycline-associated feature correlated with extensively drug-resistant tuberculosis (XDR-TB) (Figure S32).

S. aureus

Three rank I/II features were analyzed in relation to *S. aureus*, all of which were associated with rising mortality, health-related, and socioeconomic indicators (Figure S33). Notably, the plasmid rep_cluster_1017/rep_cluster_1733/rep_cluster_2100, which frequently carried the cephem resistance gene bla_Z, was selected on cephem ML models and associated with rising mortality indicators.

C. diphtheriae

C. diphtheriae carried four rank I/II features associated with the folate antibiotic drug class in the structure-adjusted ML models linked to 23 increasing/stationary indicators (Figure S34). Indicators included mortality, health, antibiotic consumption, and socioeconomic. The ARG *ermX* showed the highest connectivity to indicators ($n = 21$). Notably, skin diseases and refugee populations were both associated with increased prevalence of the resistance gene *ermX* in *C. diphtheriae*, consistent with recent studies showing a surge of cutaneous *C. diphtheriae* cases among refugee populations in Europe.[26]

DISCUSSION

In this work, we demonstrated that current and future AMR trends are correlated not only with social, economic, and environmental indicators that act as determinants of health but also with an intricate interplay involving AMR and MDR traits, MGEs, bacterial species, and hosts. Of the 32 unique features that emerged as critical threats based on their MDR profiles

Figure 6. Undirected network of gene location and resistance profiles for rank I/II features with increasing or stationary indicators

(A) Feature (pie charts indicating location distribution within isolates of each species) connected to species nodes; edges are colored by indicator trend (blue, increasing; green, stationary; and orange, both).

(B) Features (pie charts of associated resistance classes identified by the ML) connected to species; edge weight reflects the number of drug-resistance classes, and pink edges denote non-MDR features.

CellPress
OPEN ACCESS

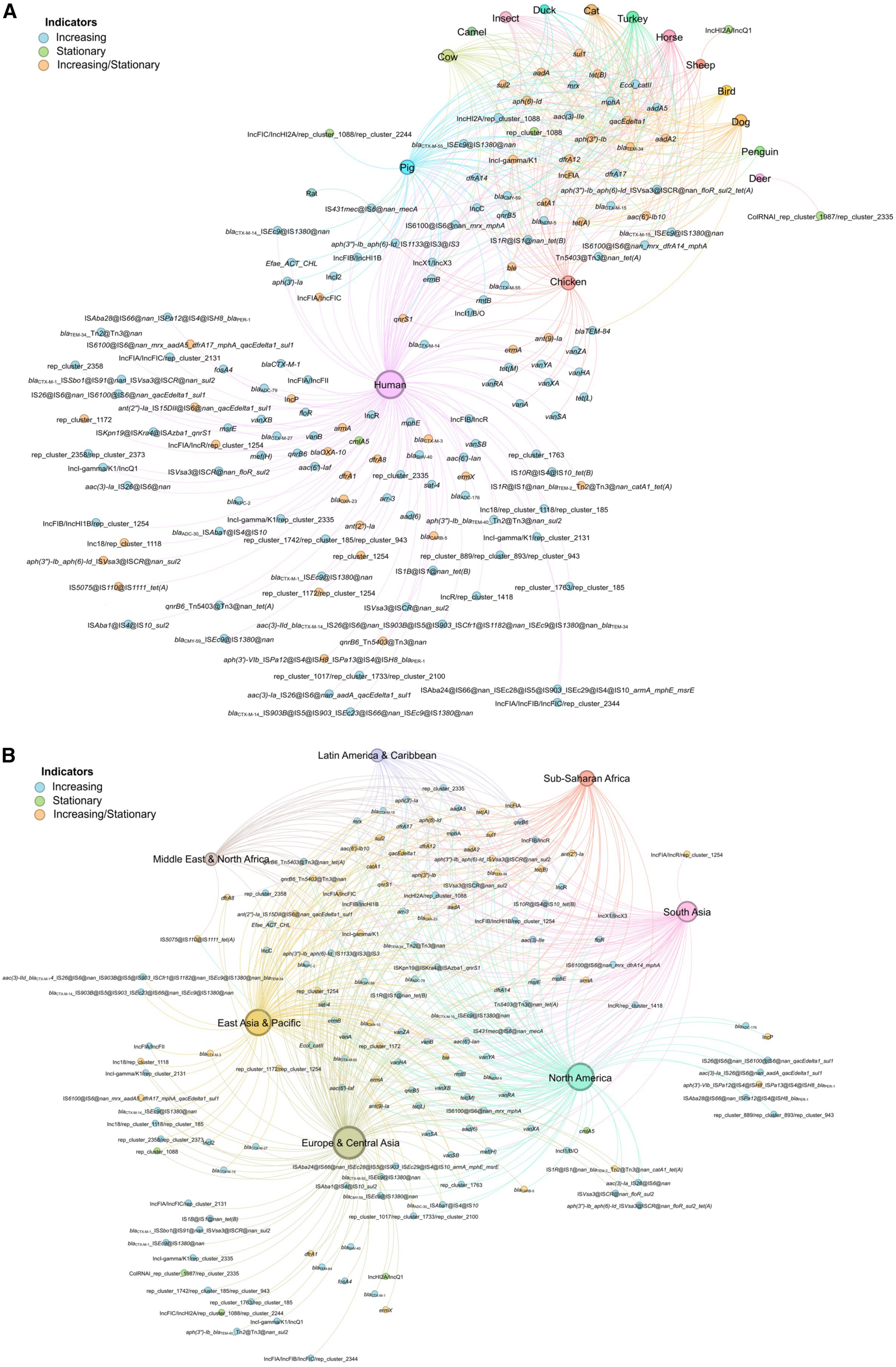

Figure 7. Undirected network of host and geographic distribution of rank I/II features with increasing or stationary indicators

(A) Feature (colored by indicator trend: blue, increasing; green, stationary; and orange, both) connected to host nodes; edges are colored by host.

(B) Feature connected to World Bank geographic region nodes; edges are colored by region. Region/host node size is proportional to the number of features.

and broad host and geographic range, their future spread was closely linked to mortality, socioeconomic, health, antibiotic consumption, and environmental indicators. Mortality-related indicators were the most common, particularly for *S. sonnei*, *K. pneumoniae*, and carbapenem-resistant *A. baumannii*, the latter two of which are known for their role in severe hospital-acquired infections (HAIs).[27,28]

Among the 32 critical threats, *S. sonnei* was linked to the largest number of indicators across all five indicator groups, highlighting the emerging threat this pathogen poses.[29]

The genes *mphA* and *ermB* in *S. sonnei* were correlated with increasing mortality, protein-energy malnutrition, population density, and livestock production. These plasmid-mediated genes confer resistance to azithromycin, used to treat severe *S. sonnei*[30] infections, and increasing azithromycin resistance is being reported in clinical settings.[31] Their projected rise, particularly in low-income regions where diarrheal disease and malnutrition are major problems, is concerning. Frequent co-location on plasmids[32] and evidence of conjugative plasmid transfer of *mphA* may explain its spread and co-selection: both genes are often found on MGEs carrying multiple resistance (β--lactams and fluoroquinolones), so treatment with β-lactams or aminoglycosides can co-select *mphA* and *ermB*.

Socioeconomic factors (e.g., GDP, population density, urban population, people using safely managed sanitation services, and poverty) were most frequently associated with *A. baumannii*, 3rd-generation cephalosporin-resistant *E. coli*, and *S. sonnei*. Furthermore, 25 of the 32 critical-threat features in *A. baumannii*, *E. coli*, and *S. sonnei* were associated with socioeconomic indicators, suggesting that economic conditions are connected to antibiotic use and resistance development. Previous studies have shown that population density is positively correlated with AMR rates in *E. coli*.[8,33] In addition, a recent report indicated that *S. sonnei* is increasingly the main etiological agent in shigellosis cases in low-middle-income countries (LMICs)[29] as economic conditions in these countries improve. The genes *sul1*, *sul2*, and *qacEdelta1* in *E. coli*, *K. pneumoniae*, *S. enterica*, and *S. sonnei* were associated with high population density, livestock production, mortality and health-related indicators, and socioeconomic disparities. This suggests that urbanization and healthcare practices contribute to their selection and spread.

Antibiotic consumption and population density (through urbanization, which increases interpersonal contact) facilitate the dissemination of ARGs and resistant bacteria.[33,34] Sulfonamides, commonly administered in human and animal medicine, are often found in wastewater treatment plants, rivers, and sewers close to highly populated areas,[34,35] most likely contributing to the prevalence of *sul1* and *sul2*. Analogously, *qacEdetla1*, a biocide resistance gene prevalent in wastewater from high-density regions,[36] reflects the use of biocides in hospitals,[37] suggesting links between ARGs, biocides, population density, and hospitals. Out of the 32 features considered critical threats, 16 were linked to antibiotic consumption, highlighting the role of antibiotic overuse in driving resistance selection. Phosphonics and macrolides consumption were the primary classes associated with these features, which were spread throughout Asia, Europe and the Pacific, North America, and Africa. Alarmingly, phosphonics have recently been upgraded to highest priority critically important antimicrobial (HPCIA) status, while macrolides were downgraded from HPCIAs to critically important antimicrobials (CIAs).[11]

While most indicators were linked to human and clinical settings, environmental factors, specifically livestock production, contributed to the persistence of 30 features in *S. sonnei*, six features in *A. baumannii*, and one feature in *K. pneumoniae*. The plasmid-located *qacEdelta1* was linked to livestock production in both *S. sonnei* and *K. pneumoniae* and was found in all seven World Bank regions, highlighting the need for a global One Health approach.[38]

Our analysis also revealed critical links between AMR features associated with MGEs and their host distribution. The same AMR features can have a mobility shift depending on the setting, pathogen, indicators, and host distribution. In carbapenem-resistant *A. baumannii*, *aph(3″)-Ib* and *aph(6)-Id* were primarily IS associated and chromosomally integrated, in line with *A. baumannii's* known acquisition of resistance through IS elements[39] in clinical settings. Conversely, in 3rd-generation cephalosporin- and fluoroquinolone-resistant *E. coli*, *S. sonnei*, and *S. enterica*, these same features were mostly on plasmids and not IS associated. Phosphonic acid is an antibiotic class widely used in livestock production (particularly in Asia and Latin America), and resistance in *S. enterica* is becoming a serious health concern,[40] suggesting livestock as a key factor of plasmid-encoded resistance in these species. The different genomic contexts (IS vs. plasmid associated) suggest divergent selective pressures and highlight the importance of tracking both mobile and chromosomal elements.[38,41] In our data, plasmid-associated AMR genes were globally widespread and concentrated in high-income, high-density regions, whereas IS- and transposon-associated elements followed a region-specific distribution linked to socioeconomic vulnerabilities.

Host distribution patterns revealed most of these features were multi-host, with several in *E. coli*, *E. faecium*, *K. pneumoniae*, *S. aureus*, *S. enterica*, and *S. sonnei* being mainly in humans and food animals, suggesting a strong association with antibiotic use in clinical and agricultural settings. These were found worldwide (median four World Bank regions), with aminoglycoside resistance associated with 68% of these features. Aminoglycosides, classed by WHO as critically important antibiotics[42] and widely used clinically,[43] are also a concern in both Africa[44] and Asia[45]; aminoglycoside-resistant *K. pneumoniae* was considered one of the top 10 pathogen-drug combinations in Africa in global burden estimates.[1]

Our analysis offers a new, actionable layer of insight to guide national AMR strategies and One Health policy. In *S. sonnei*, we identified an increasing prevalence of ARGs and associated MGEs spanning four major antibiotic classes (cephem, folate, macrolide, and penicillin). The increase was not found to be linked to antimicrobial consumption but instead correlated with socioeconomic indicators (population density and malnutrition), suggesting that reducing antibiotic usage alone would be insufficient and that governments should prioritize structural public health interventions (improving nutritional status, reducing overcrowding, and strengthening health equity in high-risk areas).

Our pipeline also provides a framework for mapping forecasted feature prevalence directly to clinical syndromes and treatment failure risks, suggesting a path toward actionable insights. In *K. pneumoniae*, we identified a distinct intersection between forecasted nitrofuran resistance features and indicators for diabetes and renal disease, two major risk factors for UTIs. Because diabetes is a primary driver of UTI incidence[46] and nitrofurans and fluoroquinolones are a global first-line therapy,[47] the co-occurrence of *qnr* genes and nitrofuran resistance features in our model suggests the elevated probability of MDR phenotypes in these patient strata, a risk that may extend to other pathogens, as diabetes has been identified as a risk factor for nitrofuran resistance in *E. coli*.[48] This mapping suggests a transition from universal empiric protocols toward risk-stratified prescribing, in settings where our model forecasts increasing resistance-feature prevalence in *K. pneumoniae*, especially in diabetic populations at elevated UTI risk.

Similarly, the association between *A. baumannii* resistance features and XDR-TB mortality indicators reveals a critical syndromic blind spot. While *A. baumannii* is not routinely screened in tuberculosis (TB) care, our mapping suggests that its presence, most likely as a nosocomial co-infection acquired during prolonged TB hospitalizations, may contribute to outcomes often attributed solely to TB treatment failure. Recent studies in tertiary settings have reported high rates of *A. baumannii* co-infections in patients with TB, with isolates displaying resistance profiles characteristic of hospital-acquired strains.[49] Furthermore, bacterial co-infection in TB significantly increases the risk of treatment failure, leading to mortality.[50] The clinical actionability is 2-fold: identifying healthcare environments where *A. baumannii* surveillance should be integrated into standard TB diagnostics and providing a rationale for escalating empiric secondary-infection coverage in patients with TB deteriorating despite appropriate anti-mycobacterial therapy. By mapping genomic features to mortality indicators, we move beyond simple surveillance to a predictive framework for HAI management in complex co-morbidity contexts.

Limitations of the study

We acknowledge that this study has some limitations that should be addressed with future research. Specifically, both the genomic and indicator data present gaps and imbalances (STAR Methods), with some hosts, years, regions, and countries overrepresented (e.g., UK) and other regions underrepresented (e.g., LMICs). Sub-Saharan Africa is particularly poorly represented yet estimated to carry the largest AMR burden globally.[1] Comparison with WHO GLASS data confirmed that the volume of available genomic data reflects regional infrastructure rather than clinical burden: HICs contribute more sequences but experience lower rates of BSI, while LMICs bear a disproportionate burden yet contribute fewer sequences. Although our model framework, which incorporates random effects for geography and years, accounts for non-independence of variables, it cannot control for extreme underrepresentation; predictions for LMICs may therefore be limited and should be interpreted with caution. Despite this, mapping genomic features to syndromic indicators allows actionable signals to be extracted from the models that transcend sampling density (see below).

Indicators were sourced from highly curated global data repositories or peer-reviewed publications (supplemental methods); heterogeneity in indicator definitions and reporting frameworks across countries remains an inherent limitation, but because indicators were modeled independently and interpreted based on temporal directionality rather than cross-domain magnitude comparisons, the impact of cross-source definitional differences on statistical inference is limited.

The forecasting method may not select ARGs with a very low representativeness; thus, recently emerging AMR genomic features may not be identified. For example, bla_{KPC-2}, bla_{KPC-4}, and bla_{KPC-8} were identified in *K. pneumoniae* by the ML framework, but only bla_{KPC-2} was suitable for forecasting analysis. The colistin gene *mcr-9.1* was present in a small number of isolates of *E. coli*, *S. enterica*, and *K. pneumoniae* but was not selected due to either low correlation with phenotypes (*E. coli* and *S. enterica*) or insufficient information for forecasting (*K. pneumoniae*). As a next step, forecastability could be extended by repeating data collection and analysis at regular intervals in order to identify ARGs that are gaining prevalence and spreading. Our analysis also did not include mutations or regulatory genes since, with such large-scale data, it would not have been possible to individually validate mutations or expression; instead, we focused on well-known ARGs with previously proven AMR functionality. Although the 16 species include both Gram-negative and Gram-positive species, the analysis has not been generalized to the entire bacterial kingdom, and expanding to a broader and more diverse range of bacterial species would be desirable in the future.

Although population structure was modeled through lineage random effects (PopPUNK clusters) and structure-aware cross-validation, a high AUC may still partially reflect residual phylogenetic signal rather than purely causal feature effects. Lineage-specific markers frequently co-occur with resistance determinants, and expansion of successful resistant clones may contribute to model accuracy. While such lineage-linked prediction remains epidemiologically meaningful for forecasting resistant population growth, it does not fully disentangle mechanistic feature-level causality from clonal background effects.

Genomic features were encoded as binary presence/absence variables, which do not capture variation in gene copy number, promoter strength, or transcriptional regulation, an important consideration given recent evidence that ARG copy number can be a critical determinant of phenotypic resistance levels.[51] Integrating transcriptomic and copy-number data at the global scale remains computationally and logistically challenging but represents a necessary next step toward refined phenotypic prediction.

Our framework provides a robust predictive model of AMR feature prevalence at the global scale but does not explicitly simulate individual horizonal gene transfer (HGT) events. Dynamic modeling frameworks, such as Markov-state transition models, offer an important complementary perspective and have demonstrated significant utility in simulating gene dynamics in data-rich, finer-scale environments, such as hospital outbreaks, where they can parameterize transition probabilities across clinical and environmental compartments.[52] Integrating our global feature-indicator mappings into such dynamic models represents a compelling direction for regional-level validation. Finally, additional MGE types (e.g., integrons) could be included, although extra care

would be needed to ensure that the overlap of genetic features does not lead to bias, for example, ARG-carrying integrons are typically plasmid borne.[53]

RESOURCE AVAILABILITY

Lead contact

Further information and requests may be directed to the corresponding author, Tania Dottorini (tania.dottorini@kcl.ac.uk).

Materials availability

This study did not generate new unique reagents.

Data and code availability

All genome assemblies and associated resistance profiles were downloaded from BV-BRC (accessed 01-09-2024). Accessions and genome IDs are given in Table S1. The code and input files (AST phenotypes and encoded AMR features) used in this study are available in the following GitHub repository: https://github.com/tan0101/AMRGlobal under https://doi.org/10.5281/zenodo.19918867.

ACKNOWLEDGMENTS

This study was supported by UKRI grants (BB/X017370/1, BB/W020424/1, MR/X009246/1, and MR/Y034422/1) and the JPIAMR Development of Innovative Strategies, Tools, Technologies, and Methods for Diagnostics and Surveillance of Antimicrobial Resistance grant (EU-JPIAMR2023-DISTOMOS-144).

AUTHOR CONTRIBUTIONS

Conceptualization, T.D.; methodology, A.M.-G., M.B., and T.D.; investigation, R.W., C.L., E.G.-A., K.P., Y.X., G.W., P.A.O., A.M.-G., and M.B.; visualization, R.W., C.L., Y.X., G.W., A.M.-G., and M.B.; supervision, T.D.; writing – original draft, A.M.-G., M.B., R.W., N.S., and T.D.; writing – review & editing, C.L., G.W., P.A.O., A.M.-G., M.B., R.W., N.S., and T.D.; funding acquisition, W.M. and T.D.

DECLARATION OF INTERESTS

The authors declare no competing interests.

STAR★METHODS

Detailed methods are provided in the online version of this paper and include the following:

- KEY RESOURCES TABLE
- METHOD DETAILS
  - ○ Genome selection
  - ○ Pathogen prevalence correlation
  - ○ Annotation and reconstruction of AMR traits
  - ○ Encoding of AMR traits
  - ○ Population structure analysis
  - ○ Machine learning analysis
  - ○ Forecasting analysis
  - ○ Prevalence calculation analysis
  - ○ Ranking criteria
  - ○ Gene structure analysis
  - ○ Visualization
  - ○ Data imbalance analysis
- QUANTIFICATION AND STATISTICAL ANALYSIS

SUPPLEMENTAL INFORMATION

Supplemental information can be found online at https://doi.org/10.1016/j.xgen.2026.101273.

Received: September 1, 2025
Revised: March 9, 2026
Accepted: May 11, 2026
Published: June 3, 2026

REFERENCES

1. Naghavi, M., Vollset, S.E., Ikuta, K.S., Swetschinski, L.R., Gray, A.P., Wool, E.E., Robles Aguilar, G., Mestrovic, T., Smith, G., Han, C., et al. (2024). Global burden of bacterial antimicrobial resistance 1990–2021: a systematic analysis with forecasts to 2050. Lancet *404*, 1199–1226. https://doi.org/10.1016/S0140-6736(24)01867-1.
2. O'Neill, J. (2016). In Tackling drug-resistant infections globally: final report and recommendations. The Review on Antimicrobial Resistance (HM Government and Wellcome Trust).
3. Frost, I., Kapoor, G., Craig, J., Liu, D., and Laxminarayan, R. (2021). Status, challenges and gaps in antimicrobial resistance surveillance around the world. J. Glob. Antimicrob. Resist. *25*, 222–226. https://doi.org/10.1016/j.jgar.2021.03.016.
4. David, S., Caballero, J.D., Couto, N., Abudahab, K., Alikhan, N.-F., Yeats, C., Underwood, A., Molloy, A., Connor, D., Shane, H.M., et al. (2025). Monitoring antimicrobial resistance trends from global genomics data: amr.watch. PLOS Glob. Publ. Health *5*, e0005256. https://doi.org/10.1371/journal.pgph.0005256.
5. Stockdale, J.E., Liu, P., and Colijn, C. (2022). The potential of genomics for infectious disease forecasting. Nat. Microbiol. *7*, 1736–1743. https://doi.org/10.1038/s41564-022-01233-6.
6. Murray, C.J.L., Ikuta, K.S., Sharara, F., Swetschinski, L., Robles Aguilar, G., Gray, A., Han, C., Bisignano, C., Rao, P., Wool, E., et al. (2022). Global burden of bacterial antimicrobial resistance in 2019: a systematic analysis. Lancet *399*, 629–655. https://doi.org/10.1016/S0140-6736(21)02724-0.
7. Ikuta, K.S., Swetschinski, L.R., Robles Aguilar, G., Sharara, F., Mestrovic, T., Gray, A.P., Davis Weaver, N., Wool, E.E., Han, C., Gershberg Hayoon, A., et al. (2022). Global mortality associated with 33 bacterial pathogens in 2019: a systematic analysis for the Global Burden of Disease Study 2019. Lancet *400*, 2221–2248. https://doi.org/10.1016/S0140-6736(22)02185-7.
8. Allel, K., Day, L., Hamilton, A., Lin, L., Furuya-Kanamori, L., Moore, C.E., Van Boeckel, T., Laxminarayan, R., and Yakob, L. (2023). Global antimicrobial-resistance drivers: an ecological country-level study at the human-animal interface. Lancet Planet. Health *7*, e291–e303. https://doi.org/10.1016/s2542-5196(23)00026-8.
9. Zhang, A.-N., Gaston, J.M., Dai, C.L., Zhao, S., Poyet, M., Groussin, M., Yin, X., Li, L.-G., van Loosdrecht, M.C.M., Topp, E., et al. (2021). An omics-based framework for assessing the health risk of antimicrobial resistance genes. Nat. Commun. *12*, 4765. https://doi.org/10.1038/s41467-021-25096-3.
10. Rice, L.B. (2008). Federal Funding for the Study of Antimicrobial Resistance in Nosocomial Pathogens: No ESKAPE. J. Infect. Dis. *197*, 1079–1081. https://doi.org/10.1086/533452.
11. Antimicrobial Resistance Division, and Impact Initiatives and Research Coordination (2024). WHO Bacterial Priority Pathogens List, 2024: Bacterial Pathogens of Public Health Importance to Guide Research, Development and Strategies to Prevent and Control Antimicrobial Resistance (World Health Organization).
12. BV-BRC (2024). The Bacterial and Viral Bioinformatics Resource Center (BV-BRC). https://www.bv-brc.org/.
13. World Health Organization (2026). Global Antimicrobial Resistance and Use Surveillance System (GLASS) Data Portal Last. https://worldhealthorg.shinyapps.io/glass-dashboard/_w_25ce728bce374f5baa190819fc849f80/#!/home.
14. Natural Earth (2024). Natural Earth Data. https://www.naturalearthdata.com/.
15. Baker, M., Zhang, X., Maciel-Guerra, A., Babaarslan, K., Dong, Y., Wang, W., Hu, Y., Renney, D., Liu, L., Li, H., et al. (2024). Convergence of resistance and evolutionary responses in Escherichia coli and Salmonella

enterica co-inhabiting chicken farms in China. Nat. Commun. *15*, 206. https://doi.org/10.1038/s41467-023-44272-1.

16. Maciel-Guerra, A., Baker, M., Hu, Y., Wang, W., Zhang, X., Rong, J., Zhang, Y., Zhang, J., Kaler, J., Renney, D., et al. (2023). Dissecting microbial communities and resistomes for interconnected humans, soil, and livestock. ISME J. *17*, 21–35. https://doi.org/10.1038/s41396-022-01315-7.
17. Wang, W., Baker, M., Hu, Y., Xu, J., Yang, D., Maciel-Guerra, A., Xue, N., Li, H., Yan, S., Li, M., et al. (2021). Whole-genome sequencing and machine learning analysis of *Staphylococcus aureus* from multiple heterogeneous sources in China reveals common genetic traits of antimicrobial resistance. mSystems *6*, e0118520. https://doi.org/10.1128/mSystems.01185-20.
18. Pearcy, N., Hu, Y., Baker, M., Maciel-Guerra, A., Xue, N., Wang, W., Kaler, J., Peng, Z., Li, F., and Dottorini, T. (2021). Genome-scale metabolic models and machine Learning reveal genetic determinants of antibiotic resistance in Escherichia coli and unravel the underlying metabolic adaptation mechanisms. mSystems *6*, e0091320–e0091920. https://doi.org/10.1128/mSystems.00913-20.
19. Peng, Z., Maciel-Guerra, A., Baker, M., Zhang, X., Hu, Y., Wang, W., Rong, J., Zhang, J., Xue, N., Barrow, P., et al. (2022). Whole-genome sequencing and gene sharing network analysis powered by machine learning identifies antibiotic resistance sharing between animals, humans and environment in livestock farming. PLoS Comput. Biol. *18*, e1010018. https://doi.org/10.1371/journal.pcbi.1010018.
20. Baker, M., Zhang, X., Maciel-Guerra, A., Dong, Y., Wang, W., Hu, Y., Renney, D., Hu, Y., Liu, L., Li, H., et al. (2023). Machine learning and metagenomics reveal shared antimicrobial resistance profiles across multiple chicken farms and abattoirs in China. Nat. Food *4*, 707–720. https://doi.org/10.1038/s43016-023-00814-w.
21. Hosmer, J.,D.W., Lemeshow, S., and Sturdivant, R.X. (2013). Applied Logistic Regression (John Wiley & Sons).
22. Chen, H., Cohen, P., and Chen, S. (2010). How Big is a Big Odds Ratio? Interpreting the Magnitudes of Odds Ratios in Epidemiological Studies. Commun. Stat. Simulat. Comput. *39*, 860–864. https://doi.org/10.1080/03610911003650383.
23. Hennart, M., Panunzi, L.G., Rodrigues, C., Gaday, Q., Baines, S.L., Barros-Pinkelnig, M., Carmi-Leroy, A., Dazas, M., Wehenkel, A.M., Didelot, X., et al. (2020). Population genomics and antimicrobial resistance in Corynebacterium diphtheriae. Genome Med. *12*, 107. https://doi.org/10.1186/s13073-020-00805-7.
24. Alcock, B.P., Raphenya, A.R., Lau, T.T.Y., Tsang, K.K., Bouchard, M., Edalatmand, A., Huynh, W., Nguyen, A.L.V., Cheng, A.A., Liu, S., et al. (2020). CARD 2020: antibiotic resistome surveillance with the comprehensive antibiotic resistance database. Nucleic Acids Res. *48*, D517–D525. https://doi.org/10.1093/nar/gkz935.
25. Levings, R.S., Hall, R.M., Lightfoot, D., and Djordjevic, S.P. (2006). *linG*, a New Integron-Associated Gene Cassette Encoding a Lincosamide Nucleotidyltransferase. Antimicrob. Agents Chemother. *50*, 3514–3515. https://doi.org/10.1128/aac.00817-06.
26. Hoefer, A., Seth-Smith, H., Palma, F., Schindler, S., Freschi, L., Dangel, A., Berger, A., D'Aeth, J., Cordery, R., Delgado-Rodriguez, E., et al. (2025). *Corynebacterium diphtheriae* Outbreak in Migrant Populations in Europe. N. Engl. J. Med. *392*, 2334–2345. https://doi.org/10.1056/NEJMoa2311981.
27. Martin, R.M., and Bachman, M.A. (2018). Colonization, Infection, and the Accessory Genome of Klebsiella pneumoniae. Front. Cell. Infect. Microbiol. *8*, 4. https://doi.org/10.3389/fcimb.2018.00004.
28. Ayobami, O., Willrich, N., Harder, T., Okeke, I.N., Eckmanns, T., and Markwart, R. (2019). The incidence and prevalence of hospital-acquired (carbapenem-resistant) Acinetobacter baumannii in Europe, Eastern Mediterranean and Africa: a systematic review and meta-analysis. Emerg. Microb. Infect. *8*, 1747–1759. https://doi.org/10.1080/22221751.2019.1698273.
29. Scott, T.A., Baker, K.S., Trotter, C., Jenkins, C., Mostowy, S., Hawkey, J., Schmidt, H., Holt, K.E., Thomson, N.R., and Baker, S. (2025). Shigella sonnei: epidemiology, evolution, pathogenesis, resistance and host interactions. Nat. Rev. Microbiol. *23*, 303–317. https://doi.org/10.1038/s41579-024-01126-x.
30. Gharpure, R., Friedman, C.R., Fialkowski, V., Collins, J.P., Strysko, J., Marsh, Z.A., Chen, J.C., Meservey, E.H., Adediran, A.A., Schroeder, M.N., et al. (2022). Azithromycin and Ciprofloxacin Treatment Outcomes During an Outbreak of Multidrug-Resistant Shigella sonnei Infections in a Retirement Community-Vermont, 2018. Clin. Infect. Dis. *74*, 455–460. https://doi.org/10.1093/cid/ciab450.
31. Baumgart, S., Phan, T., and McKew, G. (2025). Epidemiology and antimicrobial resistance rates for Shigella species in a resource-rich setting. Pathology *57*, 94–99. https://doi.org/10.1016/j.pathol.2024.07.004.
32. Asad, A., Jahan, I., Munni, M.A., Begum, R., Mukta, M.A., Saif, K., Faruque, S.N., Hayat, S., and Islam, Z. (2024). Multidrug-resistant conjugative plasmid carrying mphA confers increased antimicrobial resistance in Shigella. Sci. Rep. *14*, 6947. https://doi.org/10.1038/s41598-024-57423-1.
33. Bruinsma, N., Hutchinson, J.M., van den Bogaard, A.E., Giamarellou, H., Degener, J., and Stobberingh, E.E. (2003). Influence of population density on antibiotic resistance. J. Antimicrob. Chemother. *51*, 385–390. https://doi.org/10.1093/jac/dkg072.
34. Bougnom, B.P., McNally, A., Etoa, F.X., and Piddock, L.J. (2019). Antibiotic resistance genes are abundant and diverse in raw sewage used for urban agriculture in Africa and associated with urban population density. Environ. Pollut. *251*, 146–154. https://doi.org/10.1016/j.envpol.2019.04.056.
35. Zhuang, M., Achmon, Y., Cao, Y., Liang, X., Chen, L., Wang, H., Siame, B.A., and Leung, K.Y. (2021). Distribution of antibiotic resistance genes in the environment. Environ. Pollut. *285*, 117402. https://doi.org/10.1016/j.envpol.2021.117402.
36. Amos, G.C.A., Ploumakis, S., Zhang, L., Hawkey, P.M., Gaze, W.H., and Wellington, E.M.H. (2018). The widespread dissemination of integrons throughout bacterial communities in a riverine system. ISME J. *12*, 681–691. https://doi.org/10.1038/s41396-017-0030-8.
37. Ntshonga, P., Gobe, I., Koto, G., Strysko, J., and Paganotti, G.M. (2024). Biocide resistance in Klebsiella pneumoniae: a narrative review. Infect. Prev. Pract. *6*, 100360. https://doi.org/10.1016/j.infpip.2024.100360.
38. Djordjevic, S.P., Jarocki, V.M., Seemann, T., Cummins, M.L., Watt, A.E., Drigo, B., Wyrsch, E.R., Reid, C.J., Donner, E., and Howden, B.P. (2024). Genomic surveillance for antimicrobial resistance — a One Health perspective. Nat. Rev. Genet. *25*, 142–157. https://doi.org/10.1038/s41576-023-00649-y.
39. Jeon, J.H., Jang, K.-M., Lee, J.H., Kang, L.-W., and Lee, S.H. (2023). Transmission of antibiotic resistance genes through mobile genetic elements in Acinetobacter baumannii and gene-transfer prevention. Sci. Total Environ. *857*, 159497. https://doi.org/10.1016/j.scitotenv.2022.159497.
40. Monte, D.F.M., Doi, Y., and Lincopan, N. (2023). High prevalence and global distribution of fosfomycin resistance genes in Salmonella serovars. Lancet Microbe *4*, e968. https://doi.org/10.1016/S2666-5247(23)00261-6.
41. Castañeda-Barba, S., Top, E.M., and Stalder, T. (2024). Plasmids, a molecular cornerstone of antimicrobial resistance in the One Health era. Nat. Rev. Microbiol. *22*, 18–32. https://doi.org/10.1038/s41579-023-00926-x.
42. World Health Organization (2024). WHO List of Medically Important Antimicrobials: A Risk Management Tool for Mitigating Antimicrobial Resistance Due to Non-human Use (World Health Organization).
43. Becker, B., and Cooper, M.A. (2013). Aminoglycoside antibiotics in the 21st century. ACS Chem. Biol. *8*, 105–115. https://doi.org/10.1021/cb3005116.

44. Kimera, Z.I., Mshana, S.E., Rweyemamu, M.M., Mboera, L.E.G., and Matee, M.I.N. (2020). Antimicrobial use and resistance in food-producing animals and the environment: an African perspective. Antimicrob. Resist. Infect. Control *9*, 37. https://doi.org/10.1186/s13756-020-0697-x.

45. Harun, A.B., Khatri, B., and Karim, M.R. (2024). Phenotypic and genotypic patterns of antimicrobial resistance in livestock and poultry in South Asia: A systematic review and meta-analysis. Food Control *164*, 110575. https://doi.org/10.1016/j.foodcont.2024.110575.

46. Meatherall, B.L., Gregson, D., Ross, T., Pitout, J.D.D., and Laupland, K.B. (2009). Incidence, risk factors, and outcomes of Klebsiella pneumoniae bacteremia. Am. J. Med. *122*, 866–873. https://doi.org/10.1016/j.amjmed.2009.03.034.

47. Bono, M.J., and Leslie, S.W. (2025). In Uncomplicated Urinary Tract Infections. Treasure Island [FL], StatPearls (StatPearls Publishing LLC).

48. Abu-Humaidan, A.H., Alajlouni, Y.Y., Alajlouni, A.Y., Hamdan, O.F., Basyouni, B.L., Qasem, O.F., Riyalat, A.A., Hiasat, R.I., Magharbeh, A.H., and Alaridah, N. (2025). Antimicrobial resistance of uropathogens in diabetic patients. BMC Infect. Dis. *25*, 945. https://doi.org/10.1186/s12879-025-11358-8.

49. Bir, R., Ranjan, R., Gunasekaran, J., Chatterjee, K., Karteeka, D., Rai, A., Gupta, S., Karlapudi, P., Joshi, I., and Gupta, R.M. (2024). Prevalence of Co-infection of Culture-Proven Bacterial Pathogens in Microbiologically Confirmed Pulmonary Tuberculosis Patients From a Tertiary Care Center. Cureus *16*, e66482. https://doi.org/10.7759/cureus.66482.

50. Ishikawa, S., Igari, H., Yamagishi, K., Takayanagi, S., and Yamagishi, F. (2019). Microorganisms isolated at admission and treatment outcome in sputum smear-positive pulmonary tuberculosis. J. Infect. Chemother. *25*, 45–49. https://doi.org/10.1016/j.jiac.2018.10.005.

51. Fistarol, B.F., Gervasio, J.D., and Szöllősi, G.J. (2025). Gene copy-number features generalize better than SNPs for antimicrobial resistance prediction in Staphylococcus aureus. NPJ Antimicrob. Resist. *3*, 100. https://doi.org/10.1038/s44259-025-00172-6.

52. van Kessel, S.A.M., Wielders, C.C.H., van de Kassteele, J., Verbon, A., and Schoffelen, A.F.ISIS-AR study group; SO-ZI/AMR study group (2025). The use of a Poisson hidden Markov model for automated detection of hospital outbreaks with vancomycin-resistant enterococci in routine surveillance data. J. Hosp. Infect. *162*, 62–67. https://doi.org/10.1016/j.jhin.2025.04.025.

53. Bhat, B.A., Mir, R.A., Qadri, H., Dhiman, R., Almilaibary, A., Alkhanani, M., and Mir, M.A. (2023). Integrons in the development of antimicrobial resistance: critical review and perspectives. Front. Microbiol. *14*, 1231938–1232023. https://doi.org/10.3389/fmicb.2023.1231938.

54. World Bank Group (2024). World Bank Open Data. https://data.worldbank.org/.

55. OneHealthTrust (2024). ResistanceMap: Antibiotic Use. https://resistancemap.onehealthtrust.org/AntibioticUse.php.

56. Klein, E.Y., Impalli, I., Poleon, S., Denoel, P., Cipriano, M., Van Boeckel, T.P., Pecetta, S., Bloom, D.E., and Nandi, A. (2024). Global trends in antibiotic consumption during 2016-2023 and future projections through 2030. Proc. Natl. Acad. Sci. USA *121*, e2411919121. https://doi.org/10.1073/pnas.2411919121.

57. Our World in Data (2024). Our World in Data. https://ourworldindata.org/.

58. Lamprecht, C.S. (2024). Meteostat Python v1.2.0. 2024. https://github.com/meteostat/meteostat-python.

59. Institute for Health Metrics and Evaluation University of Oxford (2024). Global Burden of Disease Study 2021 (GBD 2021) Bacterial Antimicrobial Resistance Burden Estimates 1990-2021 and Forecasts 2022-2050 (Institute for Health Metrics and Evaluation (IHME)). https://ghdx.healthdata.org/gbd-2021.

60. University of Oxford, and University of Washington (2024). Deaths - Institute for Health Metrics and Evaluation (IHME) MICROBE. https://vizhub.healthdata.org/microbe.

61. Jain, C., Rodriguez-R, L.M., Phillippy, A.M., Konstantinidis, K.T., and Aluru, S. (2018). High throughput ANI analysis of 90K prokaryotic genomes reveals clear species boundaries. Nat. Commun. *9*, 5114. https://doi.org/10.1038/s41467-018-07641-9.

62. Camacho, C., Coulouris, G., Avagyan, V., Ma, N., Papadopoulos, J., Bealer, K., and Madden, T.L. (2009). BLAST+: architecture and applications. BMC Bioinf. *10*, 421. https://doi.org/10.1186/1471-2105-10-421.

63. Altschul, S.F., Gish, W., Miller, W., Myers, E.W., and Lipman, D.J. (1990). Basic local alignment search tool. J. Mol. Biol. *215*, 403–410. https://doi.org/10.1016/s0022-2836(05)80360-2.

64. Schwengers, O., Jelonek, L., Dieckmann, M.A., Beyvers, S., Blom, J., and Goesmann, A. (2021). Bakta: rapid and standardized annotation of bacterial genomes via alignment-free sequence identification. Microb. Genom. *7*, 000685. https://doi.org/10.1099/mgen.0.000685.

65. Robertson, J., and Nash, J.H.E. (2018). MOB-suite: software tools for clustering, reconstruction and typing of plasmids from draft assemblies. Microb. Genom. *4*, e000206. https://doi.org/10.1099/mgen.0.000206.

66. Guerrero-Araya, E., Muñoz, M., Rodríguez, C., and Paredes-Sabja, D. (2021). FastMLST: A Multi-core Tool for Multilocus Sequence Typing of Draft Genome Assemblies. Bioinform. Biol. Insights *15*, 11779322211059238. https://doi.org/10.1177/11779322211059238.

67. Chattaway, M.A., Schaefer, U., Tewolde, R., Dallman, T.J., and Jenkins, C. (2017). Identification of Escherichia coli and Shigella Species from Whole-Genome Sequences. J. Clin. Microbiol. *55*, 616–623. https://doi.org/10.1128/jcm.01790-16.

68. Lees, J.A., Harris, S.R., Tonkin-Hill, G., Gladstone, R.A., Lo, S.W., Weiser, J.N., Corander, J., Bentley, S.D., and Croucher, N.J. (2019). Fast and flexible bacterial genomic epidemiology with PopPUNK. Genome Res. *29*, 304–316. https://doi.org/10.1101/gr.241455.118.

69. Sullivan, M.J., Petty, N.K., and Beatson, S.A. (2011). Easyfig: a genome comparison visualizer. Bioinformatics *27*, 1009–1010. https://doi.org/10.1093/bioinformatics/btr039.

70. Hagberg, A., Swart, P., and S Chult, D. (2008). Exploring Network Structure, Dynamics, and Function Using NetworkX (Los Alamos National Lab.(LANL).

71. Richter, M., and Rosselló-Móra, R. (2009). Shifting the genomic gold standard for the prokaryotic species definition. Proc. Natl. Acad. Sci. USA *106*, 19126–19131. https://doi.org/10.1073/pnas.0906412106.

72. Jolley, K.A., and Maiden, M.C.J. (2010). BIGSdb: Scalable analysis of bacterial genome variation at the population level. BMC Bioinf. *11*, 595. https://doi.org/10.1186/1471-2105-11-595.

73. Jolley, K.A., Bliss, C.M., Bennett, J.S., Bratcher, H.B., Brehony, C., Colles, F.M., Wimalarathna, H., Harrison, O.B., Sheppard, S.K., Cody, A.J., and Maiden, M.C.J. (2012). Ribosomal multilocus sequence typing: universal characterization of bacteria from domain to strain. Microbiology (Read.) *158*, 1005–1015. https://doi.org/10.1099/mic.0.055459-0.

74. World Bank Group (2025). World Bank Country and Lending Groups FY26. https://datahelpdesk.worldbank.org/knowledgebase/articles/906519-world-bank-country-and-lending-groups.

75. Siguier, P., Perochon, J., Lestrade, L., Mahillon, J., and Chandler, M. (2006). ISfinder: the reference centre for bacterial insertion sequences. Nucleic Acids Res. *34*, D32–D36. https://doi.org/10.1093/nar/gkj014.

76. Robertson, J., Schonfeld, J., Bessonov, K., Bastedo, P., and Nash, J.H.E. (2023). A global survey of Salmonella plasmids and their associations with antimicrobial resistance. Microb. Genom. *9*, mgen001002. https://doi.org/10.1099/mgen.0.001002.

77. Yu, Y., Wheeler, N.E., and Barquist, L. (2025). Biased sampling driven by bacterial population structure confounds machine learning prediction of antimicrobial resistance. PLoS Biol. *23*, e3003539. https://doi.org/10.1371/journal.pbio.3003539.

78. MacKinnon, J.G. (2010). Critical values for cointegration tests. Queen's Economics Department Working Paper.

79. Kwiatkowski, D., Phillips, P.C.B., Schmidt, P., and Shin, Y. (1992). Testing the null hypothesis of stationarity against the alternative of a unit root: How sure are we that economic time series have a unit root? J. Econom. *54*, 159–178. https://doi.org/10.1016/0304-4076(92)90104-Y.

80. Plotly Technologies Inc (2015). Collaborative data science Plotlyv6.0.0. https://plot.ly.

81. Hyun, J.C., Monk, J.M., Szubin, R., Hefner, Y., and Palsson, B.O. (2023). Global pathogenomic analysis identifies known and candidate genetic antimicrobial resistance determinants in twelve species. Nat. Commun. *14*, 7690. https://doi.org/10.1038/s41467-023-43549-9.

STAR★METHODS

KEY RESOURCES TABLE

REAGENT or RESOURCE	SOURCE	IDENTIFIER
Deposited data		
Whole genome assemblies (FASTA format)	BV-BRC[12]	Table S1
AST phenotypes (resistance-susceptibility profiles, SIR format)	BV-BRC[12]	Table S1
Social, economic and environmental indicators	World Bank Data,[54] ResistanceMap,[55] Klein et al.,[56] Our World in Data,[57] Meteostat,[58] and Global Health Data Exchange[59,60]	Table S2
CARD Database v3.3.0	Alcock et al.[24]	https://card.mcmaster.ca/download
ISFinder Database		http://isfinder.biotoul.fr/
Reference genomes	NCBI	Table S20
Software and algorithms		
ML and forecasting algorithms	This study	https://github.com/tan0101/AMRGlobal; DOI: https://doi.org/10.5281/zenodo.19918867
FastANI v1.3	Jain et al.[61]	https://github.com/ParBLiSS/FastANI
BLAST + v2.16.0	Camacho et al.[62] and Altschul et al.[63]	https://blast.ncbi.nlm.nih.gov/Blast.cgi
Bakta v1.9.2	Schwengers et al.[64]	https://github.com/oschwengers/bakta
MOB-suite package v3.1.8	Robertson and Nash[65]	https://github.com/phac-nml/mob-suite
fastMLST v0.0.16	Guerrero-Araya et al.[66]	https://github.com/EnzoAndree/FastMLST
KmerID v0.1	Chattaway et al.[67]	https://github.com/ukhsa-collaboration/kmerid
PopPUNK v2.7.6	Lees et al.[68]	https://github.com/bacpop/PopPUNK
python v3.9.15		https://www.python.org/
scikit-learn v1.2.1		https://scikit-learn.org/stable/
statsmodels v0.14.5		https://imbalanced-learn.org/stable/
BioPython v1.81		https://biopython.org/
Easyfig v2.2.5	Sullivan et al.[69]	https://mjsull.github.io/Easyfig/
scipy v1.15.3		https://scipy.org/
networkx v2.8.4	Hagberg et al.[70]	https://networkx.org/
Plotly v6.0.0		https://plotly.com/

METHOD DETAILS

Genome selection

To construct a robust dataset for analysis, we retrieved an initial set of genomes from the BV-BRC database (accessed on 01-09-2024).[12] To ensure data quality and consistency, genomes were filtered according to the following criteria.

(1) only genomes with an assembly status of either “Whole Genome Shotgun” (WGS) or “Complete” were included, as these are more likely to provide comprehensive sequence data.
(2) only genomes identified in BV-BRC as ‘Good quality’ were included.
(3) only genomes whose total length fell within three standard deviations of the pathogen-specific mean were included, thereby removing outliers and potential errors in genome assembly or annotation.
(4) as an additional quality control measure, bacterial species identity for each genome was validated through Average Nucleotide Identity (ANI) comparisons using FastANI v1.34.[61] Query genomes were compared against species-specific reference assemblies taken from NCBI (accession numbers provided in Table S20). Following established benchmarks for prokaryotic species boundaries, genomes displaying <95% ANI to their respective reference were excluded from the analysis to ensure high species-level accuracy.[61,71] Identify was further corroborated using Fastmlst v0.0.16,[66] for *A. baumannii*, *C. difficile*, *C. diphtheriae*, *E. faecium*, *E. coli*, *K. pneumoniae*, *M. tuberculosis*, *N. gonorrhoeae*, *P. aeruginosa*, *S. enterica*, *S. aureus*, *S. agalactiae*, *S. pneumoniae* and *S. suis*, and against PubMLST schemes (databases downloaded 01-09-2024).[72] For *E. coli* the

Achtman scheme was used, while for *A. baumannii* the Oxford scheme was used, and for all other species only one scheme was available from PubMLST. For any isolates with MLST alleles that could not be matched (100% identity and coverage) with known alleles, most likely representative on novel allelic variants, further confirmation was sought using rMLST schema.[73] In all instances, where MLST was applicable, we observed 100% concordance between ANI-based assignment and MLST profiles. For *S. flexneri* and *S. sonnei*, additional validation steps were implemented due to their close genetic similarity to each other and to other members of the *Shigella/E. coli* species complex. Where raw genome sequences were available, species identification was performed following the procedures described by Chattaway et al.[67] Briefly, isolates were assigned by similarity matching of k-mer profiles derived from each genome to reference genomes representing each species (Table S20). These assignments were further corroborated using the *E. coli* Achtman MLST scheme, recognising that *S. sonnei* is strongly associated with CC152, *S. flexneri* with CC245 and CC243, and *S. boydii* also with CC243. Only isolates for which ANI, k-mer identification, and MLST assignments were concordant were retained for downstream analysis. For 19 *S. sonnei* isolates for which raw sequence data were unavailable, species designation was based on concordant ANI and rMLST results.

(5) genomes were only included if key metadata fields -year, host name, country and country code (for countries and overseas territories) -did not contain missing or empty values (NaN), thereby ensuring a high-quality dataset with complete contextual information for downstream analysis. This filtering step was crucial to maintain dataset integrity and enable robust temporal and geographical assessments.
(6) genome-resistance profile combinations were included only if more than 100 isolates of that pathogen with linked resistance profile were available.

After applying all the filters, we had a dataset of 45,616 and 298,178 associated resistance profiles, which were used in the analysis. The 298,178 resistance profiles corresponded to 84 antibiotics across 27 classes comprised: aminoglycoside, aminosalicylates, beta-lactam combined agents, carbapenem, cephem, diazines, fluoroquinolone, folate, fosfomycin, glycopeptide, isoxazolines, lincosamide, lipoglycopeptide, lipopeptide, macrolide, nitrofurans, nitroimidazole, organonitrogen compounds, oxazolidinone, penicillin, phenicol, pseudonomic acid, pyridines and derivatives, rifamycin, steroidal, streptogramin, tetracycline. Each isolate was associated to an average of 6.53 resistance profiles.

Pathogen prevalence correlation

Pathogen prevalence rates stratified by country were collated from GLASS data dashboard[13] for 2016–2023 (accessed 02–2026). We analyzed nine pathogen-syndrome combinations: *E. coli* blood stream infection (BSI), *E. coli* urinary tract infection (UTI), *A. baumannii* BSI, *K. pneumoniae* BSI, *K. pneumoniae* UTI, *S. enterica* BSI, *S. enterica* gastrointestinal tract infection (GI), *S. aureus* BSI, and *N. gonorrhoeae* urogenital tract infection (UG). Both sequence counts and prevalence rates were stratified by country and year. Spearman rank correlation coefficients were calculated to assess the relationship between these variables. Countries were assigned to income groups (high, upper-middle, lower-middle income or low) based on 2025 World Bank classifications.[74] Prevalence rates were averaged across income groups, and Spearman rank correlations were calculated to determine the extent to which sequencing effort serves as a proxy for disease burden. This analysis was performed to evaluate the impact of infrastructure-driven sampling bias on model inputs and to confirm the mode's ability to extract biological signals independent of sampling volume.

Annotation and reconstruction of AMR traits

To identify the ARGs within the genome dataset, we employed the Comprehensive Antibiotic Resistance Database (CARD) v3.3.0[24] and performed nucleotide-level searches against the "nucleotide_fasta_protein_homolog_model" of the CARD database using BLAST+ v2.16.0.[62] We excluded other CARD models (i.e., the protein knockout model, protein overexpression model or protein variant model). Rather than relying on ARGs already identified on BV-BRC, all genomic sequences were re-analysed to ensure consistent annotation against the same CARD version and to apply uniform, stringent criteria. Specifically, the search criteria were set at a stringent threshold of $\geq$95% identity and $\geq$95% coverage, providing higher confidence in ARGs detection while reducing false positives from sequence divergence or incomplete matches.[20] This approach enabled the detection of both well-characterized and potential variant ARGs across the dataset. In addition, we performed an analysis to check whether including the full genome rather led to false positives caused by incomplete ORFs, and did not find the presence of false positives in the selected rank I/II features.

ISs, which can mediate the mobility of ARGs, were annotated using the ISFinder database[75] with BLASTn,[63] applying the same identity and coverage thresholds (95% each) for consistency. To infer the mobility of ARGs, as done by us previously[15,16,20] considered ARG-IS pairs that were located within 5kbp of each other, as proximity within this range suggests a potential functional association.[15,20] Any genomic region containing at least one ARG-IS pair within 5 kbp was defined as an ARG-carrying IS, with those on chromosome being designated as ISs-ARGs and those on plasmids designated as ISs-Plasmid-ARGs. Where multiple ARGs and insertion sequences were located within 5kbp of each other these were kept, annotated with all the identified features and were clustered together as a single ARG-carrying IS.

Putative plasmids were reconstructed using the MOB-recon algorithm in the MOB-suite package v 3.1.8,[65] using default settings. To remove false positives, putative plasmids were filtered to remove plasmids less than 1.5kbp in length and plasmids missing both a relaxase and replicon sequence.[76] This subset of filtered plasmids was considered as confirmed plasmids in our analysis and all other

sequences (including false positive putative plasmids) were designated as chromosome located. Plasmids were only used in the analysis pipeline if they contained an ARG. All ARGs and ARG-carrying MGEs and their locations were categorised as: ARGs on the bacterial chromosome itself (herein named as ARGs-Chr); ARGs that are integrated into the chromosome nearby insertion sequences (herein named as ISs-ARGs); ARGs present nearby insertion sequences on plasmids (herein named as ISs-Plasmid-ARGs); or ARGs on plasmids but not within an insertion sequence (herein named as Plasmid-ARGs).

Encoding of AMR traits

To capture the co-occurrence of multiple mechanisms (mobility, vertical gene transfer and HGT) as well as their additive effect on resistance we adopted the following encoding pipeline. Since the same ARGs often appeared in different isolates of the same pathogen, within diverse ISs and plasmid configurations (Table S6), we aimed to prevent their correlation with the resistance phenotypes from being diluted by these variations. To do this we set an encoding approach to comprehensively consider the contribution of each ARG present in the genome accounting for possible different genomic configurations (Figure S19). Three types of features were selected as ML inputs: ARG presence, ARG-carrying IS, and ARG-carrying plasmid. For each ARG, all possible configurations, when present, were considered and fed simultaneously to the learners to capture additive and synergistic effects.

Firstly, if an ARG was found in the genome, it was included as an input feature, regardless of its location, and used as binary presence/absence input for ML, with genome location considered separately in a *post hoc* analysis.

Secondly, if an ARG was present in the genome, we accounted for mobility-associated features linked to it, by uniquely identifying ARG-carrying ISs in each isolate and pathogen based on their specific IS and ARG combinations, as described above. For example, for the gene *sul1*, the feature "IS*6100_sul1*" contained the insertion sequence IS*6100* and the ARG *sul1* within 5kbp, while the feature "IS*6100_cmx_sul1_tet(W)*" contained IS*6100* and the ARG *sul1* as well as two further ARGs *cmx* and *tet(W)* all within 5kbp of each other, both the two features were considered as separate input features. This approach resulted in between 0 and 769 unique ARG carrying IS combinations per species, with an average of 8.16 isolates per combination (range: 1 to 1006), Table S21.

Third, to account for plasmid-associated AMR phenotypes, we also included ARG-containing plasmids as input features into the ML. Given the large number of ARGs and the high diversity of ARG-plasmids combinations, encoding each ARG-plasmid combination separately would have resulted in many plasmid-associated features per pathogen. This, as commented above, would have diluted the correlation of each ARG with the resistance phenotypes, as it would create a dimensionality issue, possibly leading to ML model overfitting. To prevent this, we assessed whether each ARG-containing isolate also carried an associated plasmid with the same ARG. If so, the plasmid was added as an additional feature, with the number of ARGs serving as the feature value. These plasmid features were designated as Plasmid-ARGs.

To better illustrate the method of forming the sequence of input features to be sent to each ML model, we provide an example with *K. pneumoniae*. Among all the isolates, 530 *K. pneumoniae* isolates carried the plasmid IncFIB, each containing between 1 and 15 ARGs per isolate (with a total of 70 different ARGs across all 530 IncFIB plasmids). Across these IncFIB plasmids, 161 different ARG combinations were found, each present in between 1 and 72 isolates, with 88% occurring in fewer than 5 isolates. One example is the $bla_{\text{TEM-183}}$ present on IncFIB in *K. pneumoniae*. In 22 cases, $bla_{\text{TEM-183}}$ appeared in nine different ARG combinations within IncFIB plasmids, alongside 2 to 14 other ARGs, each combination present in 1–72 isolates. Analogous scenarios were found in many plasmids and bacterial species within our data.

For each bacterial species, after annotation of ARGs and MGEs carrying ARGs, genetic features were numerically encoded for use in statistical analysis and machine learning, as follows (Figure S20): the list of m unique ARGs was encoded as a sequence of 0/1 values (absence/presence of each ARG) per isolate; the list of n unique ARG-carrying ISs was encoded as a sequence of 0/1 values (absence/presence of each ARG-carrying IS) per isolate; the list of k unique ARG-carrying plasmids was encoded as a sequence of integers (each representing the number of ARGs in the plasmid) per isolate. The ARG-carrying plasmid features were further normalized by rescaling to the 0–1 interval, using min-max normalization. Next, the three types of features were concatenated into a $n + m + k$ list of scalars (list of encoded genome features, one per isolate). Invariant features, i.e., features exhibiting the same value in all isolates, whether associated with either resistant or susceptible phenotypes, were removed for not possessing any discriminating power (resulting in a shorter list of genome features).

Population structure analysis

Population structure was inferred using PopPUNKv2.7.6.[68] Species-specific databases were built from sequence assemblies using default parameters. A PopPUNK model was fitted using Hierarchical Density-Based Spatial Clustering of Applications with Noise (DBSCAN) (–fit-model dbscan) and refined (–fit-model refine), and isolates were assigned to PopPUNK clusters (referred to a ppcluster) based on the refined model. Cluster membership was incorporated as a random intercept in all mixed-effects models to explicitly control for lineage structure.

Machine learning analysis

For each species independently, we trained supervised Bayesian mixed-effects logistic regression models (GLMMs) to predict binary AMR phenotypes from all genomic features (ARGs-Chr, Plasmid-ARGs, ISs-Plasmid-ARGs, ISs-ARGs) to capture co-occurrence, mobility, vertical and HGT, and their additive effect on resistance. Each model was trained separately for each bacterial species

and for each antimicrobial for which sufficient data were available with ML pipeline (Figure S21) applied identically across all species and antimicrobials.

AMR phenotypes were derived from AST data using the standard SIR classification (susceptible, intermediate and resistant) and used as class labels.

Intermediate phenotypes were excluded to ensure robust binary classification, and only resistant and susceptible isolates were retained. Including a third class (intermediate) would have weakened the training process due to the ambiguity of intermediate phenotype interpretation and could have propagated uncertainty through downstream analyses.

Isolates were grouped into two classes based on binary AST phenotype (Figure S21). If classes were highly unbalanced (less than 10% of samples in one class), models were not fitted. The model was fitted using BinomialBayesMixedGLM from the statsmodels Python package, with genomic features modeled as fixed effects and country, host, year and PopPUNK cluster modeled as random effects (encoded as categorical variables), to control for population structure, sampling bias, and non-homogeneity of variance.[77] Interclass correlation coefficients (ICCs) were calculated for each model to quantify the proportions of model variance attributable to these effects (and reported in Table S7). ICCs for each random effect k were calculated as

$$ICC_k = \frac{\sigma_k^2}{\sum_j \sigma_j^2 + \frac{\pi^2}{3}}$$

where σ^2 represents the variance, j is the set of all random effects, and the fixed logistic residual variance is calculated as $\frac{\pi^2}{3}$.

To manage the high-dimensionality of the feature space while maintaining biological traceability, we avoided explicit dimensionality reduction or feature selection and instead, relied on Bayesian regularisation: a Gaussian prior on fixed effects (equivalent to an L2/Ridge penalty of 2.0) and a log-normal prior on variance components (penalty of 0.5), limiting large coefficients and compensating for collinearity. This shrinkage approach was specifically chosen over L1/LASSO regularisation to ensure stability in the presence of high collinearity; unlike LASSO, which may arbitrarily remove one of two highly correlated genomic features, whereas a Bayesian Ridge framework shrinks coefficients proportionately, preserving biological context.

No further arbitrary feature selection was applied, ensuring that all retained predictors remain directly traceable to the original genomic data.

Model performance was evaluated using stratified group k-fold cross-validation (k = 5), with folds grouped by PopPUNK cluster to prevent population structure leakage. Where fewer than five clusters were present, the number of folds was reduced accordingly. The cross-validation (CV) procedure was repeated across 50 trials to obtain robust estimates of performance and feature importance. Within each fold, models were trained on the training split and evaluated on the held-out test split. Folds lacking both resistant and susceptible cases in either set were excluded.

Performance was assessed using ROC-AUC, precision-recall AUC, accuracy ((true positive + true negative)/(positive + negative)), sensitivity (true positive rate: true positive/positives) specificity (true negative rate: true negatives/negatives) and precision, and reported as mean and standard deviation across all folds and trials.

To obtain final parameters for inference of the genomic features importance, a final model was fitted using all data and genomic feature importance was quantified using odds ratios, $OR = e^{\beta}$, using a standard transformation of the fixed-effects coefficients. Confidence intervals were calculated using the cross-validation output, and final per feature estimates (confidence intervals) were obtained by averaging across folds and trials. Genomic features were retained for further analysis if they had an OR $\geq$ 2 with a lower 95% CI > 1, indicating statistical significance, and if the feature was positively associated with resistance in more than 80% of cross-validation trials, indicating a stable odds ratio.

Forecasting analysis

We developed an approach for identifying health, environmental and socio-economic indicators with a strong correlation with prevalence of the genomic features potentially conferring resistance identified by ML. Subsequently, we developed a method that first forecasts future changes of such indicators over a period of 25 years (up to 2050) and then uses such changes to predict future changes in prevalence of said genomic features. Our approach can be divided into eight steps (Figure S22).

(1) We obtained indicators from multiple sources (World Bank Data,[54] ResistanceMap,[55] Klein et al.,[56] Meteostat data,[58] Our World in Data,[57] and Global Health Data Exchange[59,60]). Indicators include antibiotic consumption, socioeconomic information, mortality, health-related and environmental risk factors collected from the same countries as the genomics data obtained from BV-BRC. The indicators were categorised into six groups: 63 health-related, 41 socioeconomic, 19 antibiotic consumption, 11 environmental, 6 governance and 972 mortality (including estimated deaths and DALYs), see Table S2 for indicator definitions and sources. While the death and DALYs data was complete, with estimates for each country and year for which genomes were obtained, the other 143 data indicators had a mean completeness per country of 93% (range 75%–97%). However, there were no statistical differences in coverage by region or income bracket.

(2) For each pathogen, we grouped the individual ASTs into antibiotic classes and aggregated the genome features belonging to individual AST predictors into a single set. The prevalence for each genomic feature selected by ML was calculated as the number of resistant isolates carrying the AMR genomic feature relative to the total population of resistant and susceptible

isolates regardless of if the AMR genomic feature is carried or not. Notably, prevalence was calculated considering each antibiotic class rather than each individual antibiotic. To avoid problems related to small sample sizes, we excluded country-year combinations with less than 6 isolates from the prevalence calculation.

In parallel, all indicator values retrieved from the databases were normalized using a min-max normalization approach for each individual country, considering all the years for each indicator.

(3) For each pathogen, data pertaining to all the available countries and all the available years were aggregated, and an initial investigation was performed by computing Pearson correlation coefficients between the prevalence of each genomic feature and any potentially pairable indicator. To avoid problems related to small sample sizes, correlations were only calculated where there were at least five years of indicator values along with genome feature prevalence data for the same years. Pairings covered a variable range between a minimum acceptable of 5 years, and a maximum of 21 years. Only those prevalence-indicator pairings showing strong correlation (Pearson coefficient $\rho > |0.5|$, and a p-value <0.05) were retained for further analysis.
(4) A linear regression analysis was run on pairs selected using the Pearson coefficient, to obtain for each pair a linear model describing the correlation between genomic feature prevalence and the paired indicator. Through its slope and intercept parameters, each model would allow to predict feature prevalence from the indicator value.
(5) To control false positives arising from large-scale testing of genomic features across multiple indicators, we applied a permutation-based significance testing procedure combined with Benjamini–Hochberg FDR control. For each indicator–feature regression, the response variable was randomly permuted 1000 times while keeping predictors fixed. This generated an empirical null distribution of test statistics from which permutation-based p-values were calculated. These permutation-derived p-values were then adjusted using the Benjamini–Hochberg procedure implemented in the statsmodels Python package, with an FDR threshold of 10% (adjusted p-values ≤ 0.1). Only feature–indicator pairs remaining significant after FDR correction were retained for the next stage of forecasting. FDR correction was applied within each antibiotic model rather than globally across all tests, as tests within each model share a common biological context while tests across different species and antibiotics are largely independent.

(6) Another regression analysis (linear and non-linear) was performed, this time to model the trend of variation over time of each selected indicator. Using the available indicator yearly data, curve fitting was performed, considering either a linear function or a non-linear one, the final choice of function being driven by the highest R^2 result (and as long as >0.8). The best fit model for each indicator, defined by its parameter's values and associated confidence intervals, was then used as a starting point to run a Monte Carlo simulation (10,000 iterations) to predict the value of the indicator until 2050. The forecast itself would be returned as the mean of 10,000 simulations and 5th and 95th percentiles.
(7) Once forecasts for the indicators were obtained, the linear models developed in step (4) were used to calculate, again by Monte Carlo simulation, the forecast for the genomic prevalence associated to the indicator over the same years (up to 2050).
(8) For each pair comprising genome prevalence and indicator processed through steps 3–6, both datasets expressed as time series were also tested for stationarity, using the ADF (Augmented Dickey-Fuller) and KPSS (Kwiatkowski-Phillips-Schmidt-Shin) test statistics.[78,79] The ADF tests rejects the hypothesis that the series is nonstationary while the KPSS test rejects the hypothesis that the series is stationary. In this work, a p-value of 0.05 was used in both tests. The test for stationary behavior (or rather, lack thereof) was useful to further assess the reliability of forecasts which would see a genome prevalence increase or decrease by 2050 (i.e., the forecast would be strengthened by statistic evidence of lack of stationarity).

Prevalence calculation analysis

To evaluate robustness to denominator choice, the full forecasting pipeline was repeated using alternative prevalence formulations while holding all other modeling components constant.

The primary prevalence metric was defined as the joint burden measure:

$$P(F \cap R) = \frac{F_R}{N},$$

Where F_R denotes the number of resistant isolates carrying the feature and N denotes the total number of isolates (resistant plus susceptible).

The marginal prevalence was defined as

$$P(F) = \frac{F_{R+S}}{N},$$

Where F_{R+S} denotes the total number of isolates carrying the feature irrespective of phenotype.

The conditional prevalence among resistant isolates was defined as

$$P(F|R) = \frac{F_R}{N_R},$$

Where N_R denotes the total number of resistant isolates.

These definitions isolate joint population burden, overall genomic carriage, and within-resistance enrichment, respectively. Comparing results across these formulations ensured that identified trends were not artifacts of a single denominator choice.

Ranking criteria

Genomic features were ranked based on a risk framework similar to the one proposed by Zhang et al.[9] The framework consists of a decision tree based on two criteria: (1) gene mobility (genomic feature present in an MGE and/or plasmid in over 10% of the isolates); (2) presence/absence in ESKAPE pathogens or WHO critical/high pathogens lists (host pathogenicity). This framework yields three risk categories. Genomic features that do not meet the first criterion (mobility) are assigned to rank III (lowest rank); the ones that meet the first but not the second criterion (host pathogenicity) are assigned to rank II; while those that meet the first and the second, rank I (highest rank). These criteria are computed based on the genomics data used in this work. Critical threats were classified by satisfying all the following: (1) were identified as MDR; (2) persistence in at least 30% of time periods; (3) present in at least two host types; (4) selected by the structure-adjusted mixed-effects models as associated with resistance in at least two bacterial species; and (5) were geographically widespread (i.e., found in at least four World Bank regions).

Gene structure analysis

To annotate the gene structure across bacterial species, for isolates containing the AMR feature IS*6100_mrx_aadA5_dfrA17_mphA_qacEdelta1_sul1*, the DNA sequences of the contigs containing the feature were annotated with Bakta v1.9.2,[64] using settings '–force –skip-crispr'. Exemplar contigs containing the feature (three for each pathogen) were aligned and visualised using Easyfig v2.2.5.[69]

Visualization

Maps were created using public domain shape files from Natural Earth.[14] Undirected graphs were created using NetworkXv2.8.4[70] and Gephi v0.10.1 to visualize the connection between the AMR features and their pathogens, regions, hosts and indicator groups. Sankey diagrams were created using Plotlyv6.0.0.[80]

Data imbalance analysis

The BV-BRC dataset has underlying imbalance across resistance profiles, geographies, bacterial species, hosts and years, so data rebalancing analysis was an essential task for robust analysis. Wherever possible we removed, bypassed or reduced imbalance as outlined below.

Bioinformatics

In the data gathering and preparation part of the pipeline (Figure 2, Part 1; Figure S20), the identification of ARGs and MGEs carrying ARGs was performed within individual genomes of each bacterial species, making this step inherently unaffected by sample imbalance across species.

Machine learning

In the ML (Figure 2, Part 2; Figure S21), the GLM model parameter estimates can become biased where extreme class imbalance is present. To control for this, models where one class contained <10% of the samples were excluded from analysis. Furthermore, in our work, genotype-phenotype analyses were conducted separately for each bacterial species, rather than aggregating all species to avoid potential imbalances due to underrepresented or overrepresented species. Finally, when addressing the identification of correlations between genotypes and phenotypes, we aggregated datasets across geographical locations and hosts (while controlling for these covariates as random effects). Our approach is similar to Hyun et al.[81] who also used machine learning methods to investigate AMR determinants with imbalanced data across bacterial species and resistance phenotypes as we do, and like us ran ML independently for each species, aggregated across hosts and geography).

Forecasting

In the forecasting analysis (Figure 2, Part 3; Figure S23), there were imbalances across geographic regions and years as a result of data gaps in both the genomic data and the social, economic and environmental indicator data. For example, only 11 *E. coli* genomes were available from Rwanda, all collected in 2014, while China had many years of *S. pneumoniae* genome data collected between 1995 and 2016 but was missing several indicator data for that period of time (e.g., ''People with basic handwashing facilities including soap and water (% of population)''; ''Income share held by lowest 20%''; ''Population living in slums (% of urban population)''; ''Diabetes prevalence (% of population ages 20 to 79)''). These data imbalance issues are similar to those experienced by Murray et al.[6] and Naghavi et al.[1] which both estimated deaths and DALYs attributable and associated with AMR, and also had large imbalances across geographical regions with data gaps particularly in LMIC regions. The aforementioned studies addressed this problem with a discussion of the limitations and in the case of Murray et al.[6] by excluding source-locations-year-age combinations with fewer than 5 cases and zero deaths. In our analysis, we followed a similar approach, by excluding country-year combinations that featured ≤5 isolates usable for prevalence calculation (Figure S22). In addition, when these prevalences were then used as inputs into the

forecasting analysis, countries were further excluded if they did not have at least 5 country-year data points (i.e., prevalences calculated for at least 5 years) (Figure S22).

Statistical testing

In all other statistical tests, we acknowledged imbalance where present and performed normalisation where possible. Specifically,

- We acknowledged data gaps in the indicator data and presented both the mean and range to acknowledge the asymmetric spread (This refers to the descriptive statistics of the social determinants of health data, results section).
- As the number of genomes per country was nonnormal distribution, we presented both the mean and range to acknowledge the asymmetric spread. (This refers to the descriptive statistics of the distributions of genomes across different countries, results section)
- The number of ML selected features was compared across Gram-negative and Gram-positive groupings using a Mann Whitney test, suitable for unequal sample sizes (This refers to the Mann-Whitney test used to compare the number of genomic features in Gram-negative and Gram-positive species, results section).
- In the linear regression steps of the forecasting analysis, Monte Carlo simulations were used to address uncertainty in the regression parameters due to imbalanced data and data gaps (This refers to the Monte Carlo simulations used for the forecasting analysis, results section).
- The genomic locations of AMR features were compared using the Kruskal-Wallis test, which is suitable for data with unequal sample size and variance. This test was conducted independently in each species to avoid bias due to unequal bacterial species distributions (This refers to the Kruskal-Wallis test with Dunns adjustment used to compare the genomic locations of AMR features across species, results section).
- To address imbalances between numbers of isolates featuring resistance to different classes of antibiotics within each bacterial species, we normalised the data by considering the "representativeness" of feature-pathogen pairs, as we acknowledged that the number of resistance classes per feature was influenced by the imbalance in the number of isolates for each species. "Representativeness" was defined for each pathogen and for the selected rank I/II features, as the number of resistance classes of the aggregated ML models (with an AUC >0.7) in which the feature was selected, relative to the number in which it was present in the input. Representativeness values were then compared using the Kruskal-Wallis test, which is suitable for data with unequal sample size and variance (This refers to the Kruskal-Wallis with Dunns adjustment used to compare the representativeness across species, results section).

QUANTIFICATION AND STATISTICAL ANALYSIS

ML pipeline statistics were implemented as described in detail in the method details section above. Spearman rank correlations reported in Table S4 were calculated in Microsoft Excel (Microsoft 365). Permutation testing and FDR correction for multiple testing were conducted in Python using the *statsmodels* package v0.14.5, with statistical significance set at FDR $\leq$0.1. Other statistical comparisons (specifically Mann Whitney, Kruskal-Wallis, proportion z-tests and Fisher's exact tests), all reported in the results section, were two-tailed, with significance set at $p \leq 0.05$ (adjusted for multiple testing where appropriate). All analyses were performed using the SciPy (v1.16.3) package and *statsmodels* (v0.14.5).

Cell Genomics, Volume *6*

Supplemental information

Genomic and socioeconomic drivers of antimicrobial resistance forecast to 2050

Michelle Baker, Alexandre Maciel-Guerra, Ruoqi Wang, Chengchang Luo, Yan Xu, Enzo Guerrero-Araya, Weihua Meng, Ge Wu, Komkiew Pinpimai, Peter Anthony Oyom, Nicola Senin, and Tania Dottorini

Supplemental Information for Genomic and socioeconomic drivers of antimicrobial resistance forecast to 2050

Baker *et al.*

Corresponding author email: tania.dottorini@kcl.ac.uk

This PDF file includes:

Figures S1 to S34
Tables S4, S20 and S21
Supplemental Data 1
Supplemental Methods 1

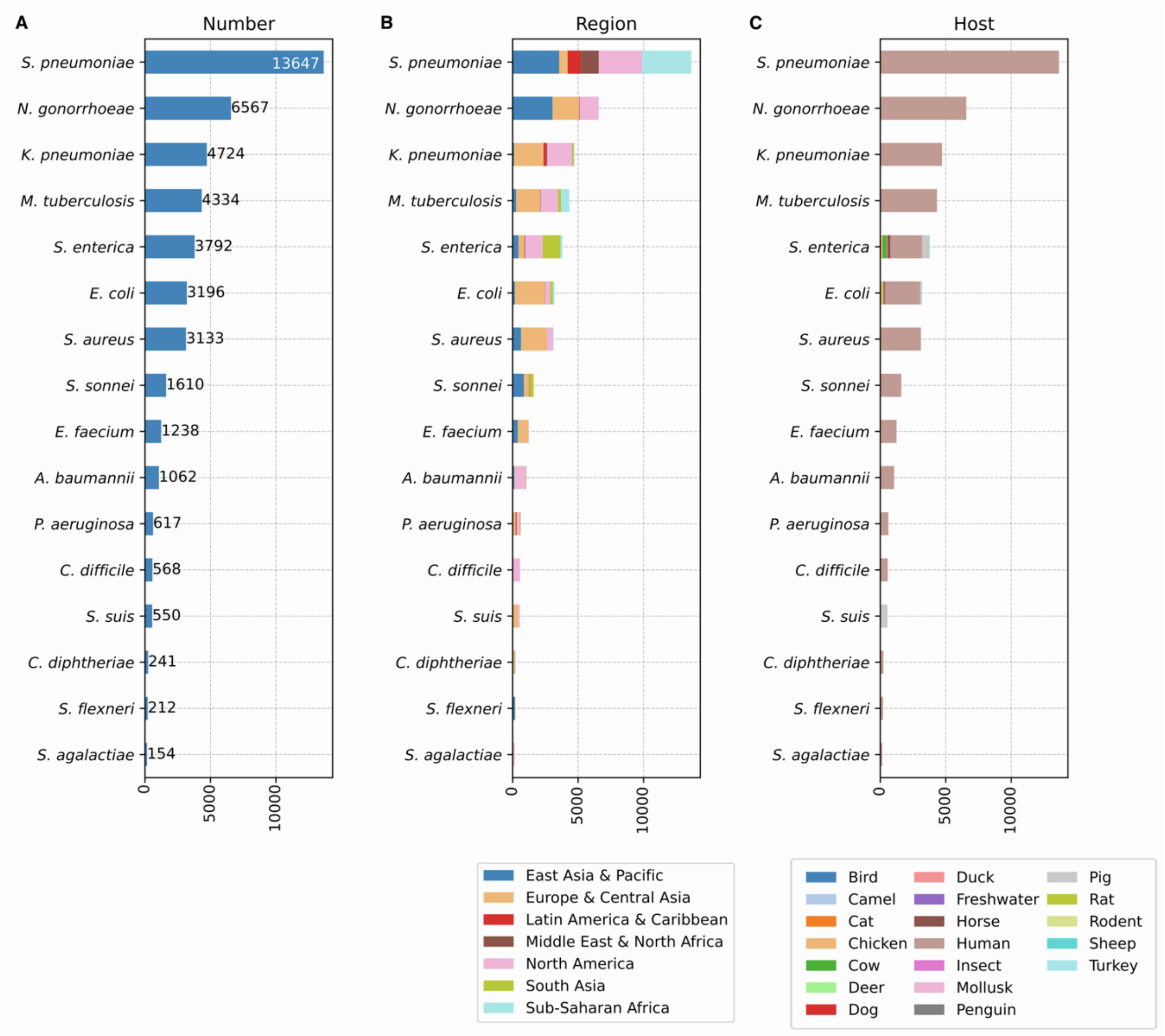

Figure S1. Bar plots showing distribution of isolates selected for analysis from BV-BRC database, related to Figure 1. Bacterial species were included if there were more than 100 high quality DNA sequences with accompanying resistance/susceptibility profiles in the database (see **STAR Methods**). (A) Number of isolates per bacterial species. (B) Region of origin for included isolates, bars coloured by World Bank regions. (C) Host of isolates, bars coloured by host.

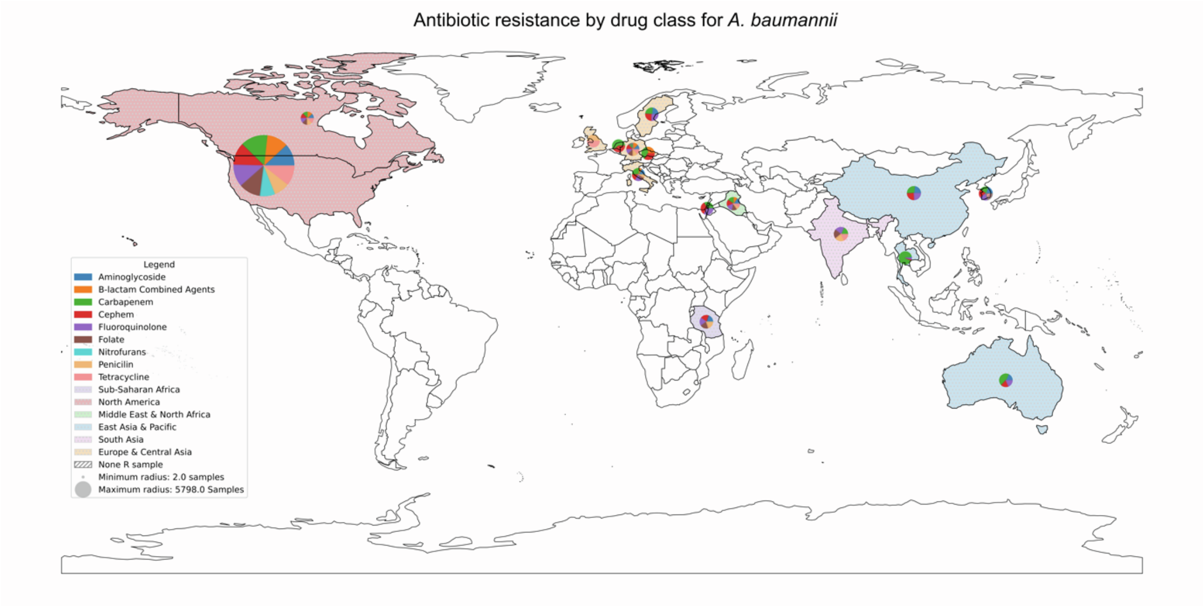

Figure S2. **Geographic distributions of 1,062 *A. baumannii* isolates with 12,074 associated resistance susceptibility phenotypic profiles obtained from the BV-BRC database[1] related to Figure 1.** The pie chart on each country shows the proportion of resistance profiles by class for each country, with the size of the chart indicating the overall number of genomes. The world map[2] has been divided according to the seven World Bank regions (East Asia and Pacific; Europe and Central Asia; Latin America and Caribbean; Middle East and North Africa; North America; South Asia; Sub-Saharan Africa).

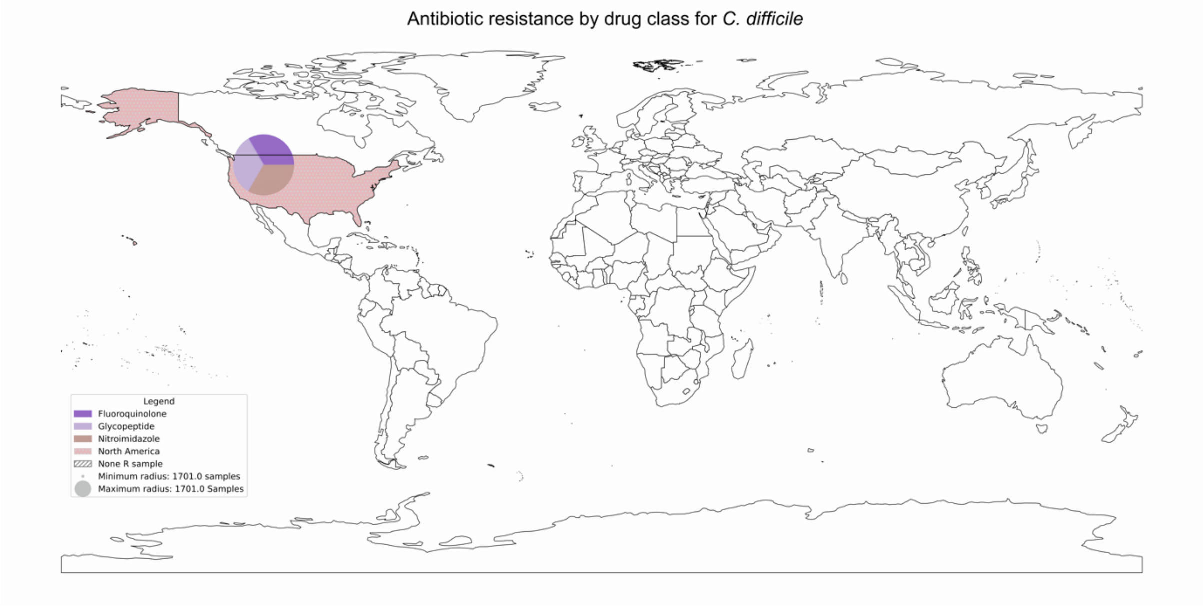

Figure S3. **Geographic distributions of 568 *C. difficile* isolates with 1,701 associated resistance susceptibility phenotypic profiles obtained from the BV-BRC database[1], related to Figure 1**. The pie chart on each country shows the proportion of resistance profiles by class for each country, with the size of the chart indicating the overall number of genomes. The world map[2] has been divided according to the seven World Bank regions (East Asia and Pacific; Europe and Central Asia; Latin America and Caribbean; Middle East and North Africa; North America; South Asia; Sub-Saharan Africa).

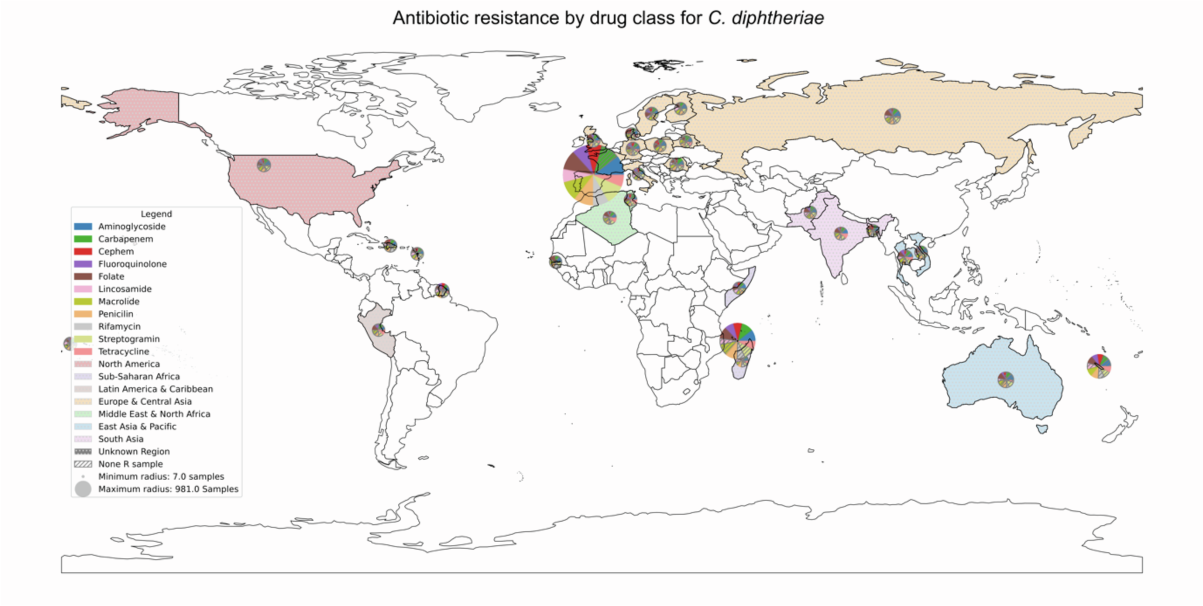

Figure S4. Geographic distributions of 241 *C. diphtheriae* isolates with 3,899 associated resistance susceptibility phenotypic profiles obtained from the BV-BRC database[1], related to Figure 1. The pie chart on each country shows the proportion of resistance profiles by class for each country, with the size of the chart indicating the overall number of genomes. The world map[2] has been divided according to the seven World Bank regions (East Asia and Pacific; Europe and Central Asia; Latin America and Caribbean; Middle East and North Africa; North America; South Asia; Sub-Saharan Africa).

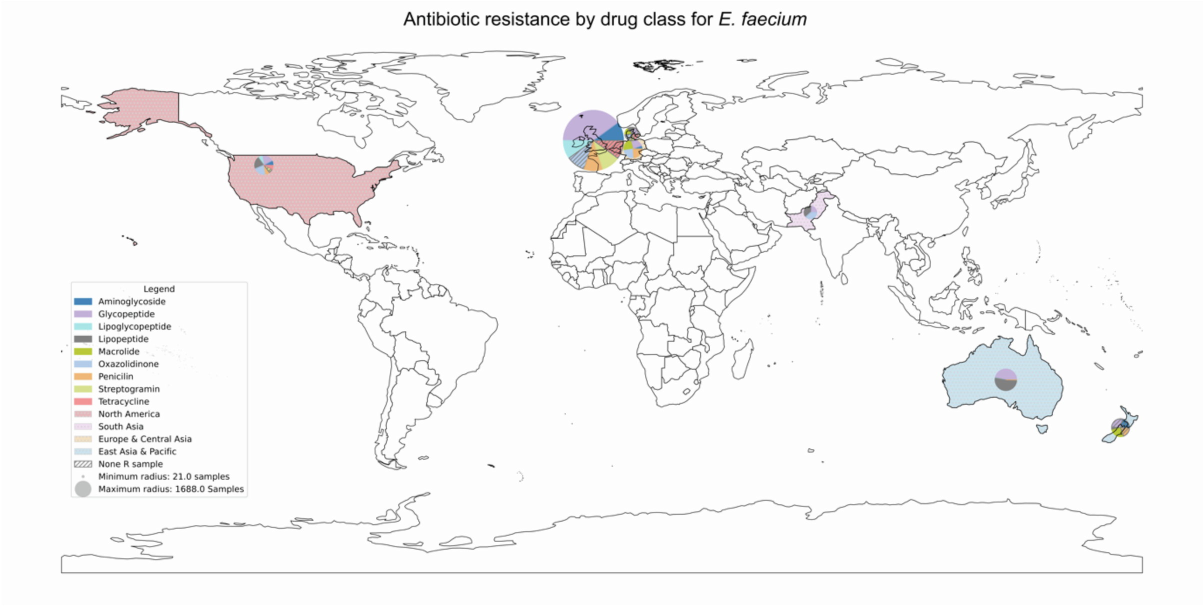

Figure S5. Geographic distributions of 1,238 *E. faecium* isolates with 2,960 associated resistance susceptibility phenotypic profiles obtained from the BV-BRC database[1], related to Figure 1. The pie chart on each country shows the proportion of resistance profiles by class for each country, with the size of the chart indicating the overall number of genomes. The world map[2] has been divided according to the seven World Bank regions (East Asia and Pacific; Europe and Central Asia; Latin America and Caribbean; Middle East and North Africa; North America; South Asia; Sub-Saharan Africa).

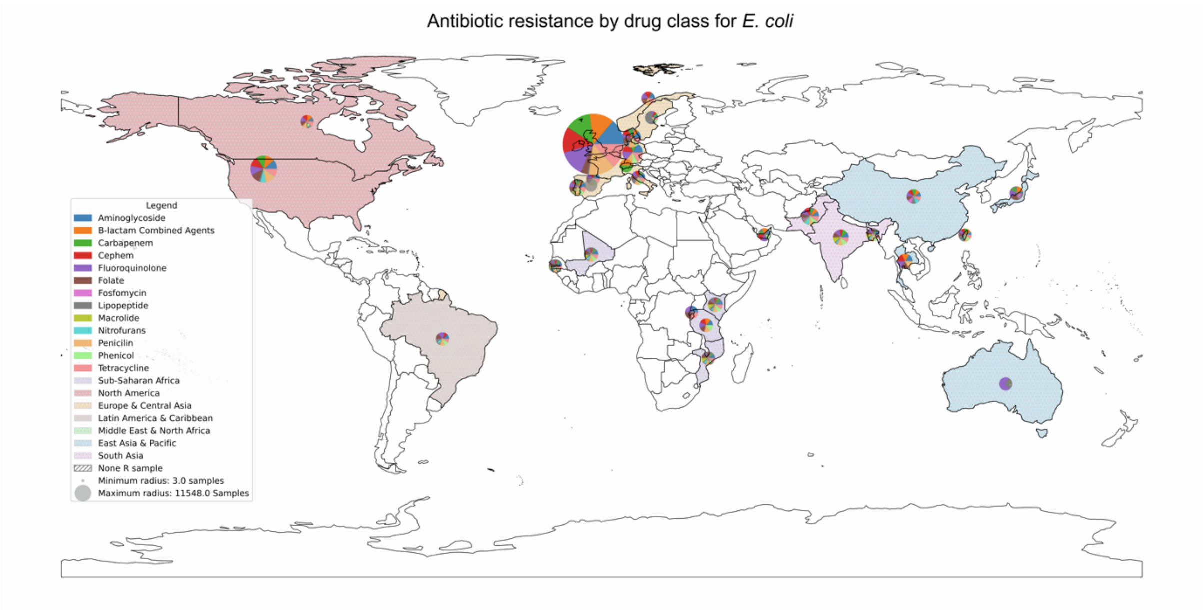

Figure S6. Geographic distributions of 3,196 *E. coli* isolates with 39,855 associated resistance susceptibility phenotypic profiles obtained from the BV-BRC database[1], related to Figure 1. The pie chart on each country shows the proportion of resistance profiles by class for each country, with the size of the chart indicating the overall number of genomes. The world map[2] has been divided according to the seven World Bank regions (East Asia and Pacific; Europe and Central Asia; Latin America and Caribbean; Middle East and North Africa; North America; South Asia; Sub-Saharan Africa).

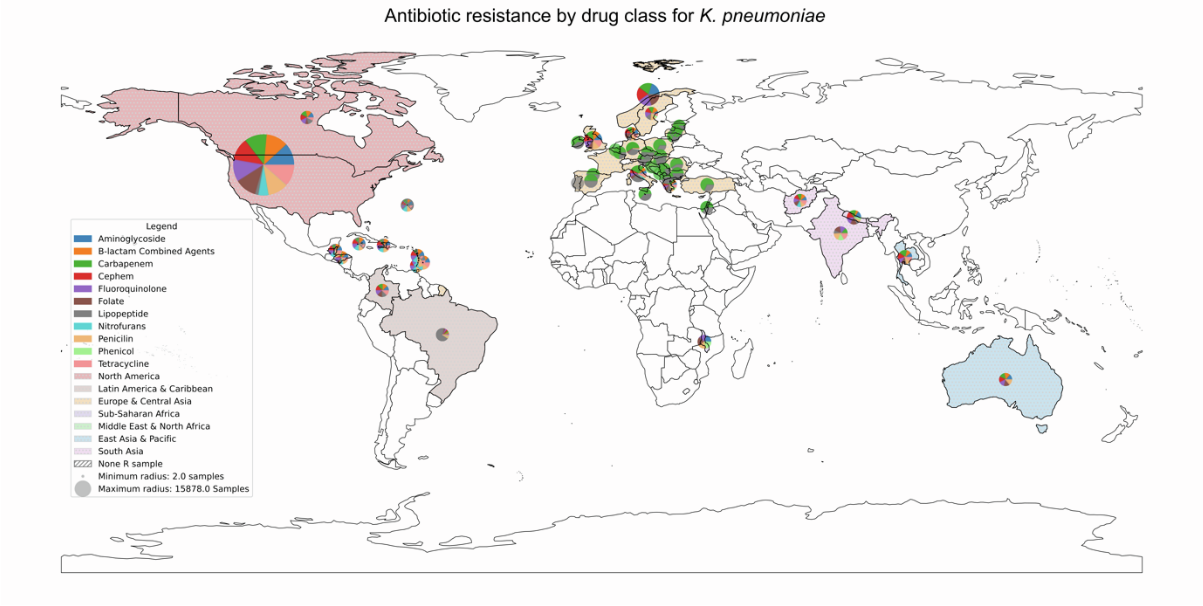

Figure S7. Geographic distributions of 4,724 *K. pneumoniae* isolates with 51,896 associated resistance susceptibility phenotypic profiles obtained from the BV-BRC database[1] related to Figure 1. The pie chart on each country shows the proportion of resistance profiles by class for each country, with the size of the chart indicating the overall number of genomes. The world map[2] has been divided according to the seven World Bank regions (East Asia and Pacific; Europe and Central Asia; Latin America and Caribbean; Middle East and North Africa; North America; South Asia; Sub-Saharan Africa).

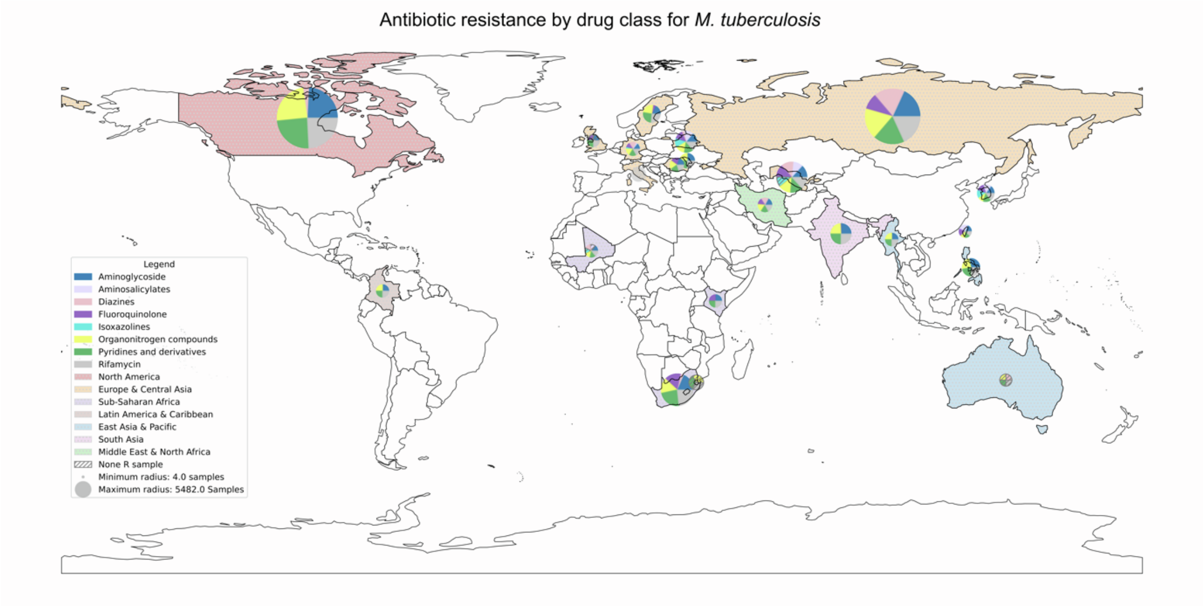

Figure S8. Geographic distributions of 4,334 *M. tuberculosis* isolates with 24,710 associated resistance susceptibility phenotypic profiles obtained from the BV-BRC database[1], related to Figure 1. The pie chart on each country shows the proportion of resistance profiles by class for each country, with the size of the chart indicating the overall number of genomes. The world map[2] has been divided according to the seven World Bank regions (East Asia and Pacific; Europe and Central Asia; Latin America and Caribbean; Middle East and North Africa; North America; South Asia; Sub-Saharan Africa).

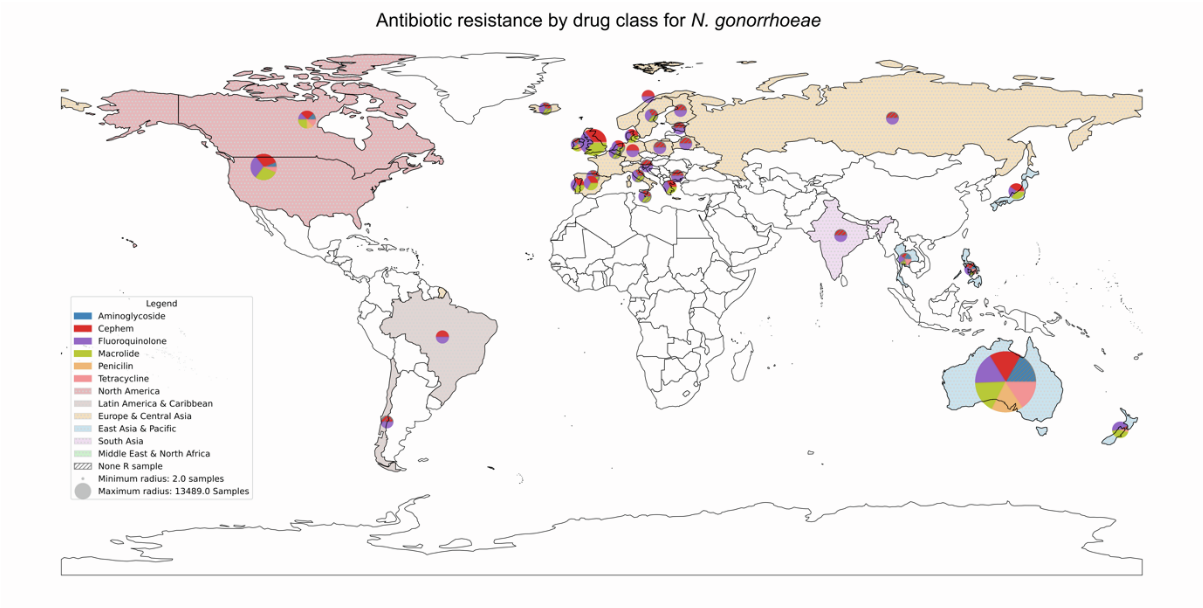

Figure S9. Geographic distributions of 6,567 *N. gonorrhoeae* isolates with 30,741 associated resistance susceptibility phenotypic profiles obtained from the BV-BRC database[1], related to Figure 1. The pie chart on each country shows the proportion of resistance profiles by class for each country, with the size of the chart indicating the overall number of genomes. The world map[2] has been divided according to the seven World Bank regions (East Asia and Pacific; Europe and Central Asia; Latin America and Caribbean; Middle East and North Africa; North America; South Asia; Sub-Saharan Africa).

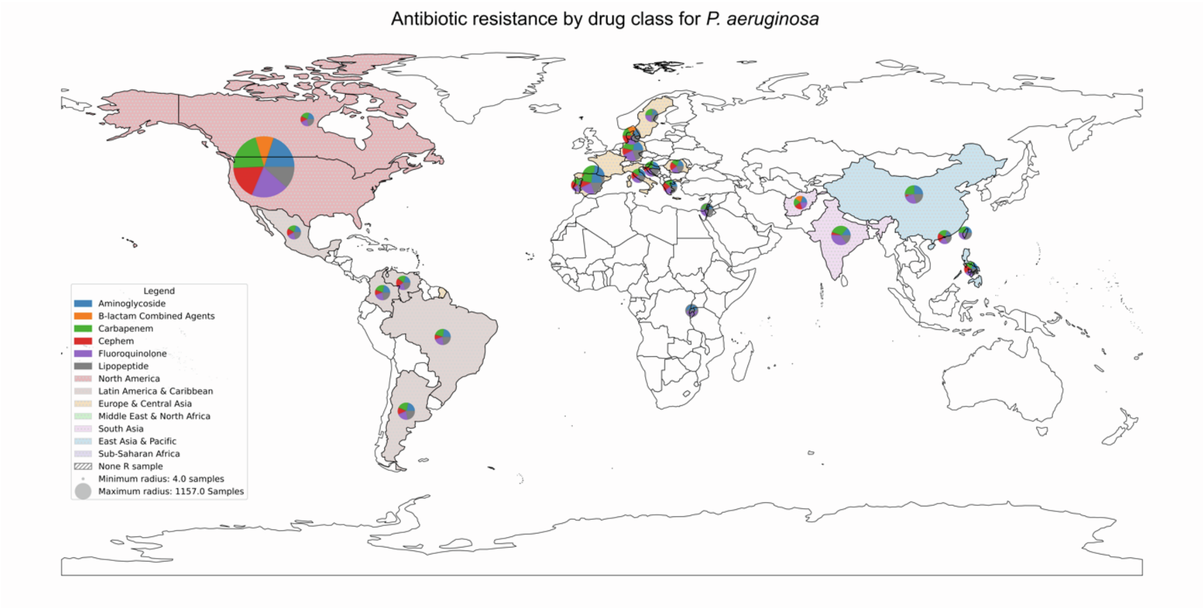

Figure S10. **Geographic distributions of 617 *P. aeruginosa* isolates with 3,531 associated resistance susceptibility phenotypic profiles obtained from the BV-BRC database[1], related to Figure 1**. The pie chart on each country shows the proportion of resistance profiles by class for each country, with the size of the chart indicating the overall number of genomes. The world map[2] has been divided according to the seven World Bank regions (East Asia and Pacific; Europe and Central Asia; Latin America and Caribbean; Middle East and North Africa; North America; South Asia; Sub-Saharan Africa).

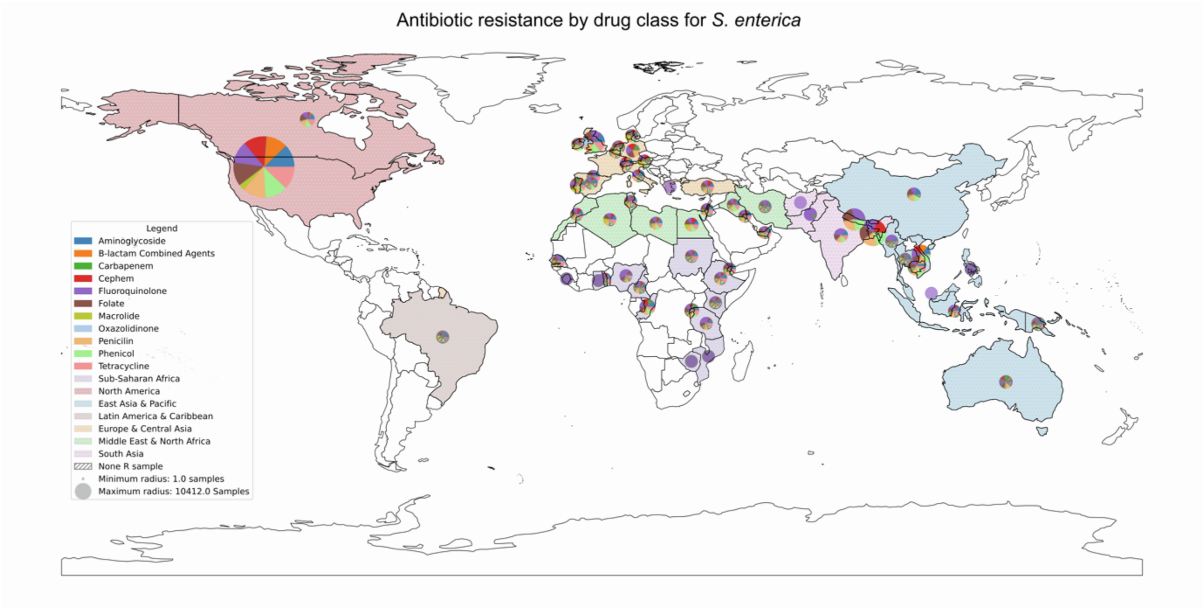

Figure S11. Geographic distributions of 3792 *S. enterica* isolates with 33,562 associated resistance susceptibility phenotypic profiles obtained from the BV-BRC database[1], related to Figure 1. The pie chart on each country shows the proportion of resistance profiles by class for each country, with the size of the chart indicating the overall number of genomes. The world map[2] has been divided according to the seven World Bank regions (East Asia and Pacific; Europe and Central Asia; Latin America and Caribbean; Middle East and North Africa; North America; South Asia; Sub-Saharan Africa).

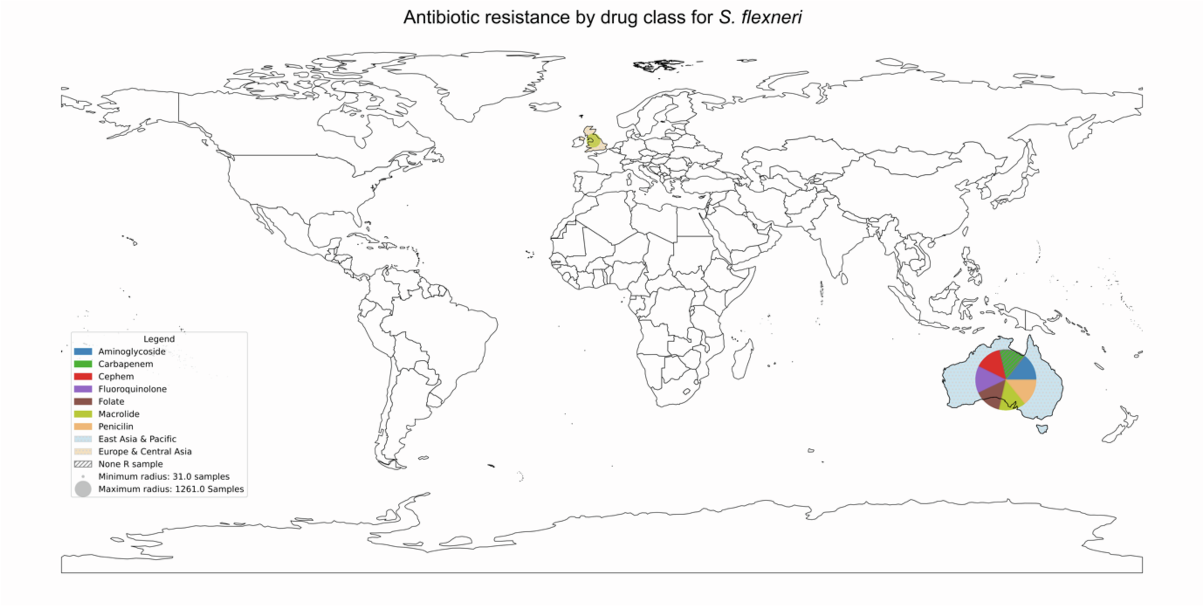

Figure S12. Geographic distributions of 202 *S. flexneri* isolates with 1,399 associated resistance susceptibility phenotypic profiles obtained from the BV-BRC database[1], related to Figure 1. The pie chart on each country shows the proportion of resistance profiles by class for each country, with the size of the chart indicating the overall number of genomes. The world map[2] has been divided according to the seven World Bank regions (East Asia and Pacific; Europe and Central Asia; Latin America and Caribbean; Middle East and North Africa; North America; South Asia; Sub-Saharan Africa).

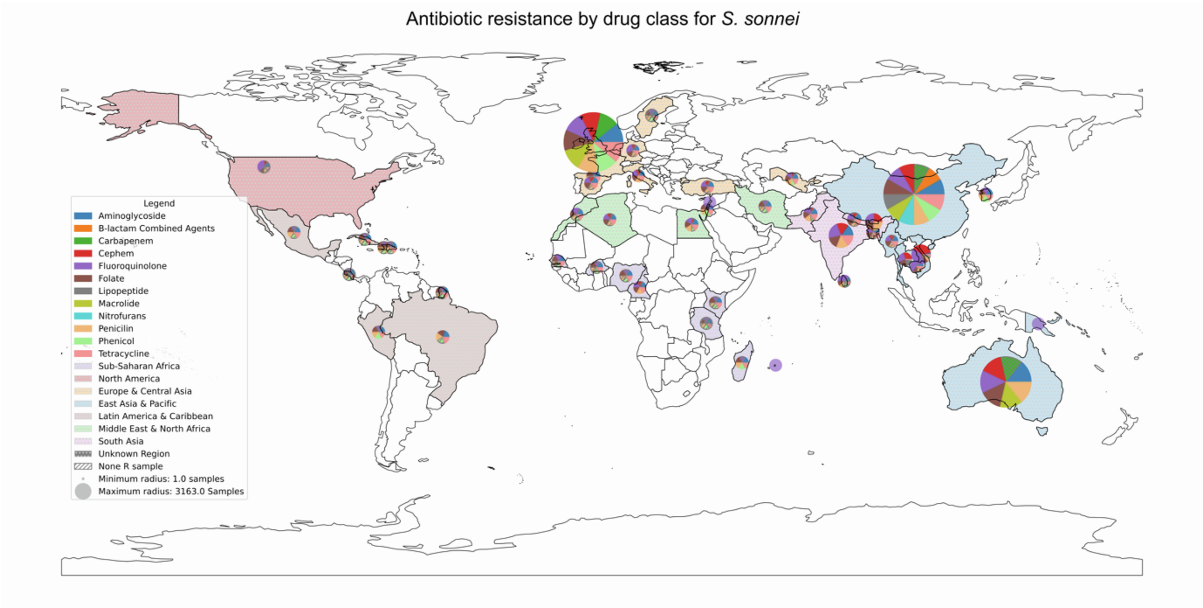

Figure S13. Geographic distributions of 1591 *S. sonnei* isolates with 17,617 associated resistance susceptibility phenotypic profiles obtained from the BV-BRC database[1], related to Figure 1. The pie chart on each country shows the proportion of resistance profiles by class for each country, with the size of the chart indicating the overall number of genomes. The world map[2] has been divided according to the seven World Bank regions (East Asia and Pacific; Europe and Central Asia; Latin America and Caribbean; Middle East and North Africa; North America; South Asia; Sub-Saharan Africa).

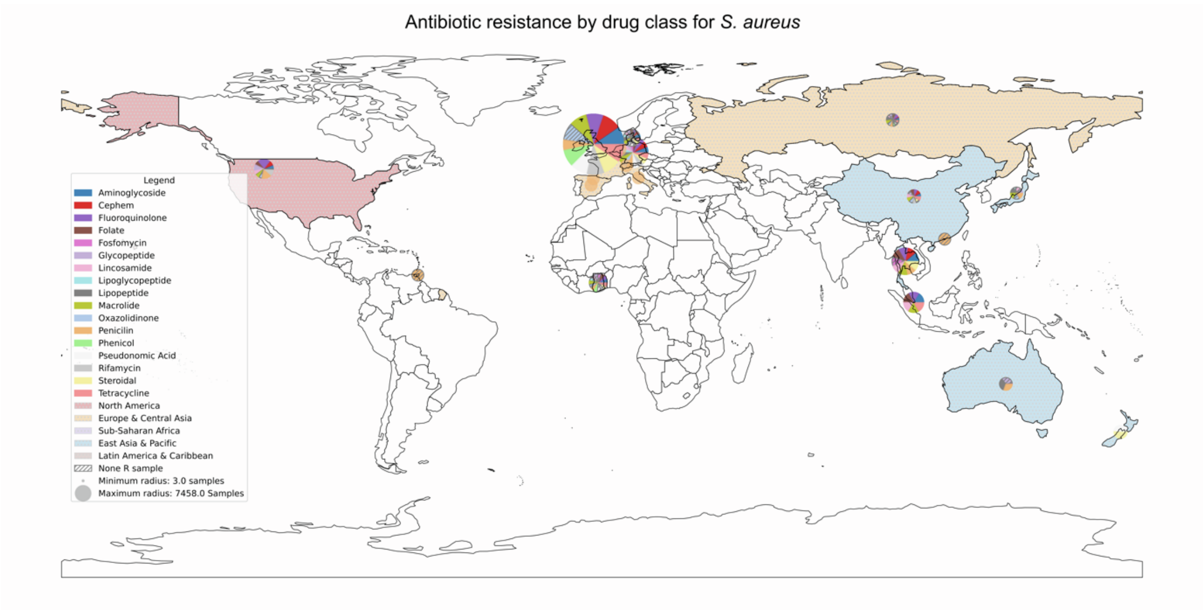

Figure S14. Geographic distributions of 3,133 *S. aureus* isolates with 18,839 associated resistance susceptibility phenotypic profiles obtained from the BV-BRC database[1], related to Figure 1. The pie chart on each country shows the proportion of resistance profiles by class for each country, with the size of the chart indicating the overall number of genomes. The world map[2] has been divided according to the seven World Bank regions (East Asia and Pacific; Europe and Central Asia; Latin America and Caribbean; Middle East and North Africa; North America; South Asia; Sub-Saharan Africa).

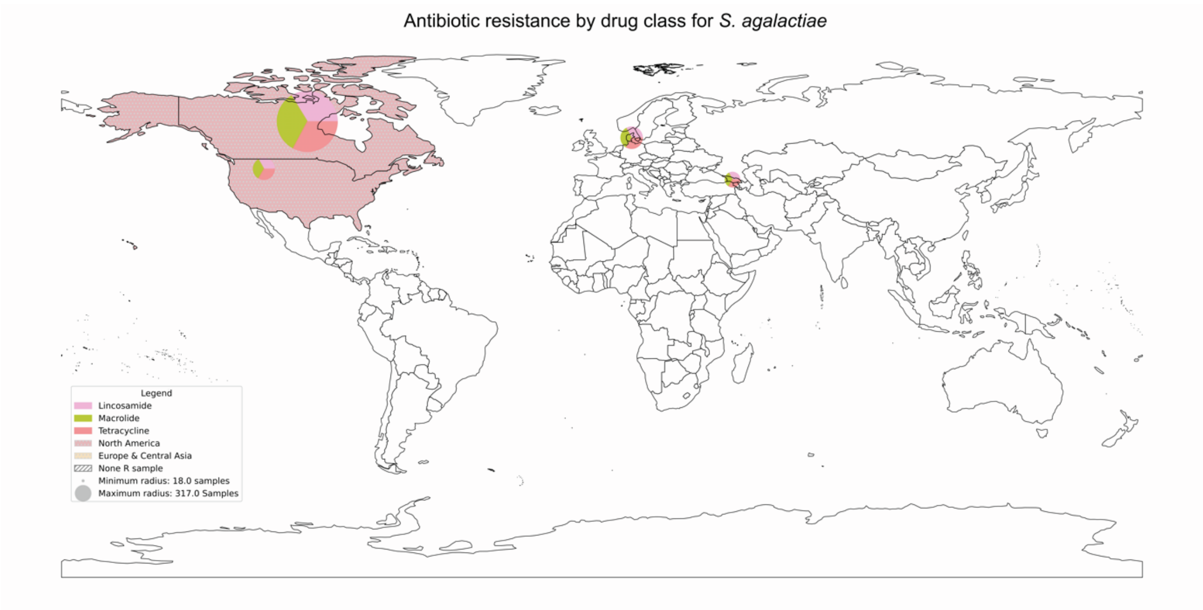

Figure S15. Geographic distributions of 154 *S. agalactiae* isolates with 458 associated resistance susceptibility phenotypic profiles obtained from the BV-BRC database[1], related to Figure 1. The pie chart on each country shows the proportion of resistance profiles by class for each country, with the size of the chart indicating the overall number of genomes. The world map[2] has been divided according to the seven World Bank regions (East Asia and Pacific; Europe and Central Asia; Latin America and Caribbean; Middle East and North Africa; North America; South Asia; Sub-Saharan Africa).

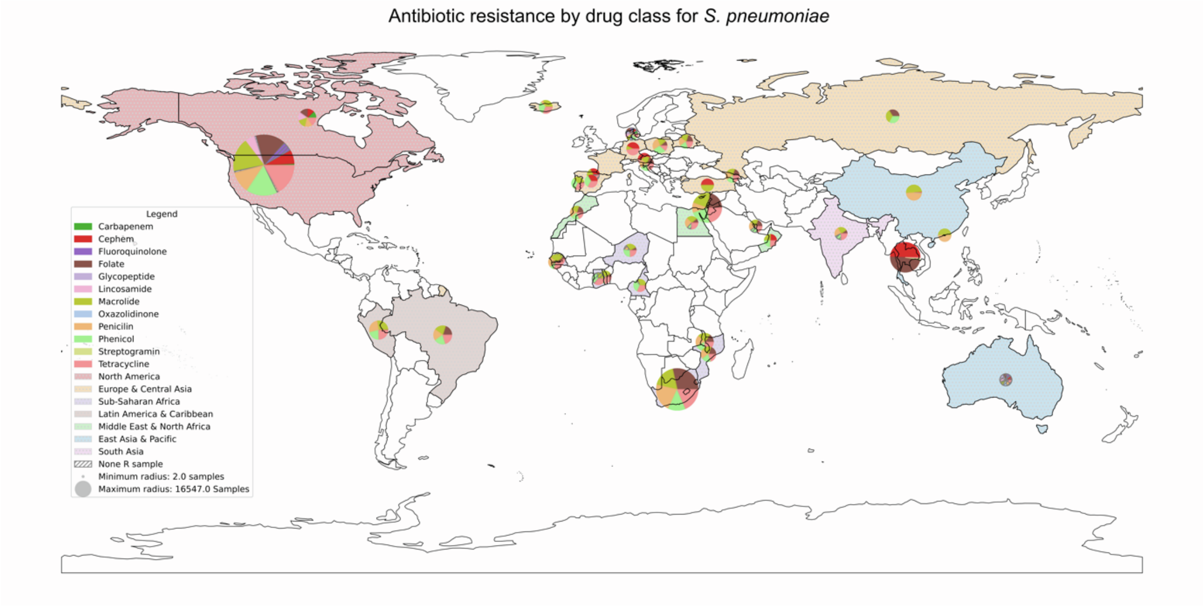

Figure S16. Geographic distributions of 13,647 *S. pneumoniae* isolates with 52,756 associated resistance susceptibility phenotypic profiles obtained from the BV-BRC database[1], related to Figure 1. The pie chart on each country shows the proportion of resistance profiles by class for each country, with the size of the chart indicating the overall number of genomes. The world map[2] has been divided according to the seven World Bank regions (East Asia and Pacific; Europe and Central Asia; Latin America and Caribbean; Middle East and North Africa; North America; South Asia; Sub-Saharan Africa).

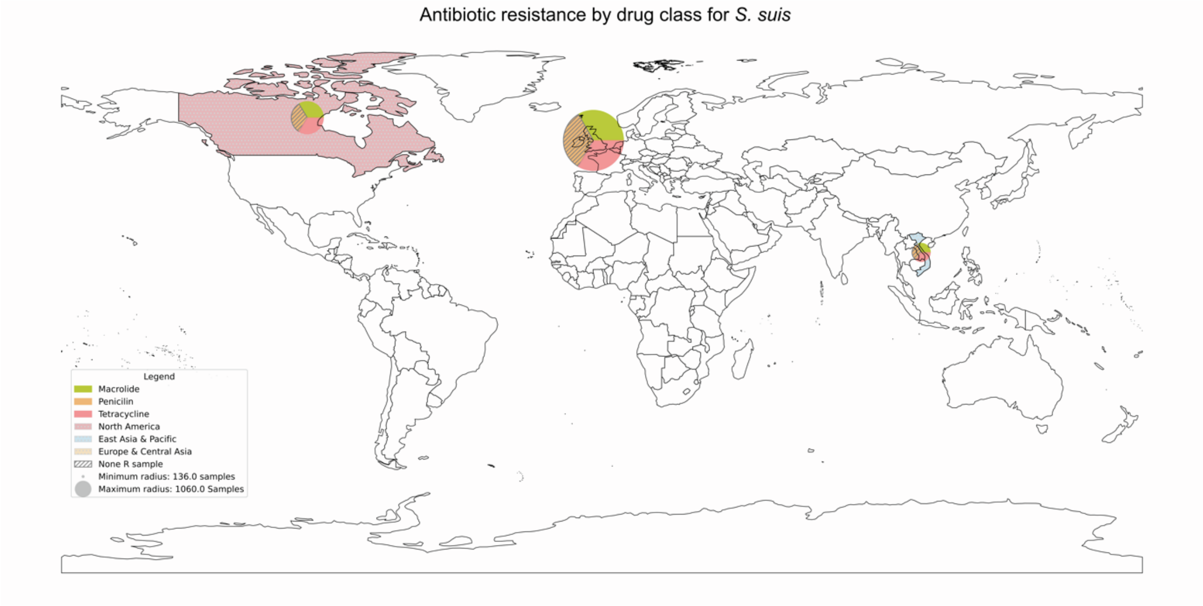

Figure S17. Geographic distributions of 550 *S. suis* isolates with 2,180 associated resistance susceptibility phenotypic profiles obtained from the BV-BRC database[1], related to Figure 1. The pie chart on each country shows the proportion of resistance profiles by class for each country, with the size of the chart indicating the overall number of genomes. The world map[2] has been divided according to the seven World Bank regions (East Asia and Pacific; Europe and Central Asia; Latin America and Caribbean; Middle East and North Africa; North America; South Asia; Sub-Saharan Africa).

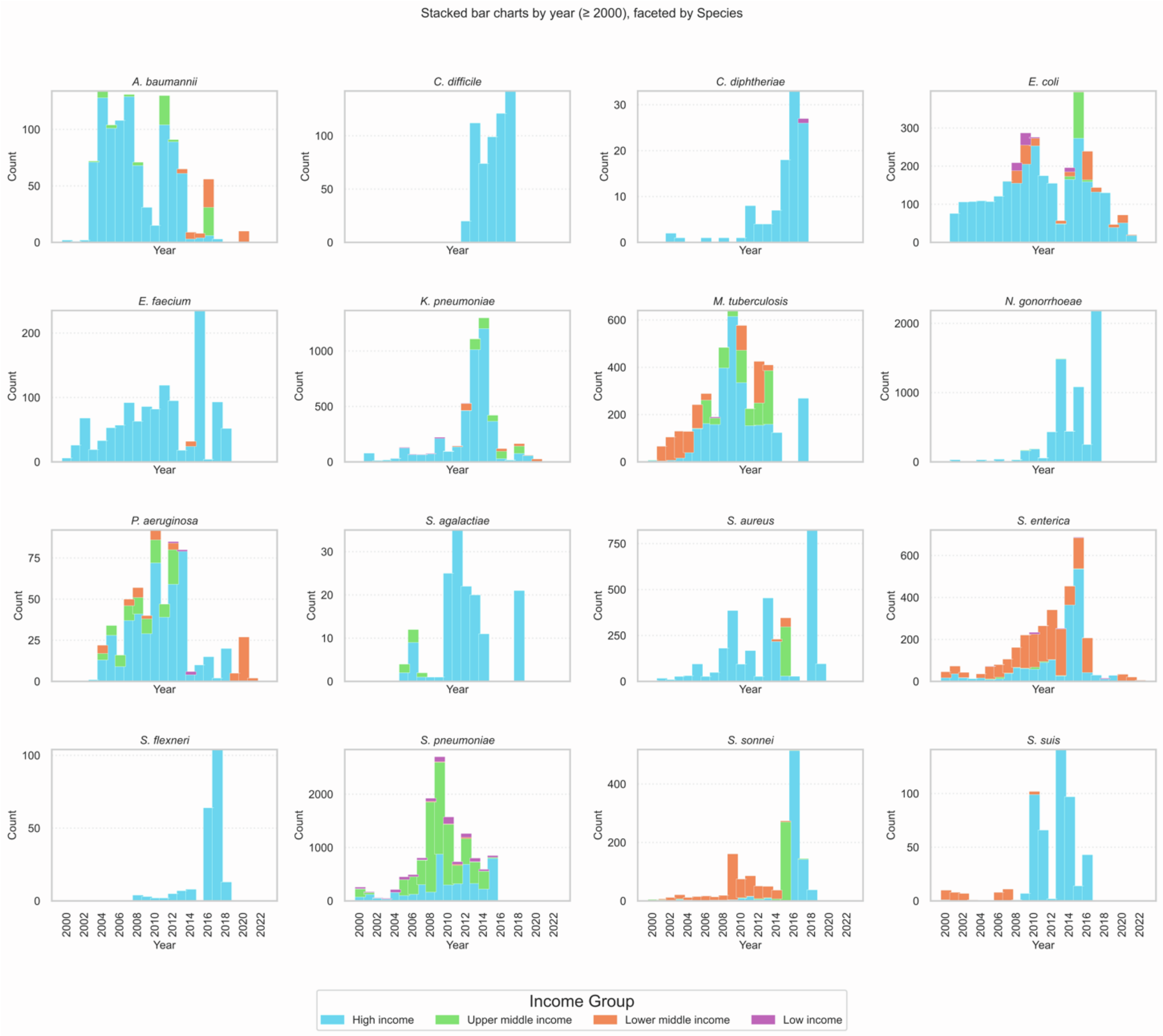

Figure S18. Stacked bar charts showing the distribution of sequence data across years by income group, related to STAR Methods. Data are truncated to only show data from the year 2000 onwards for visual clarity, one subpanel is shown for each bacterial species, stratified by World Bank income group, based on the isolates used, **Table S1**. Counts are stacked by income group: high income (blue), upper middle income (green), lower middle income (orange), and low income (red).

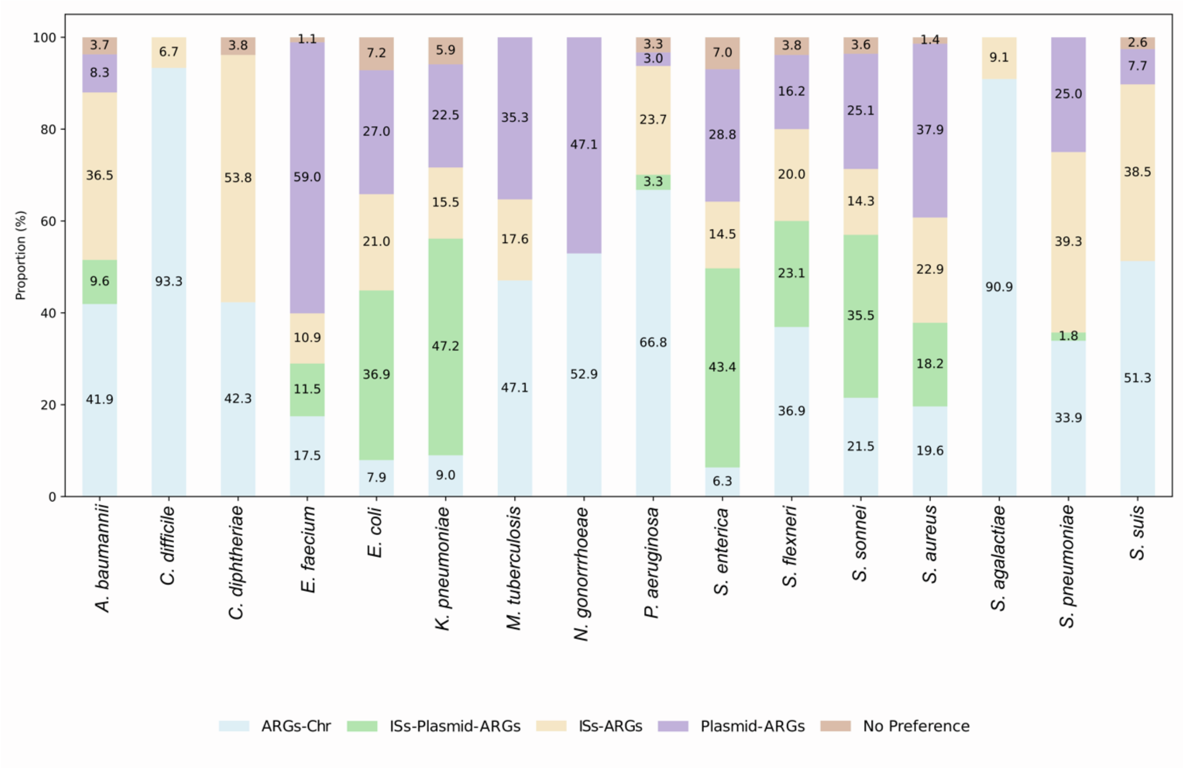

Figure S19. **Stacked bar plot showing the proportion of AMR genomic traits and their predominant location across 16 bacterial species, related to Figure 1.** Each feature can be present in four locations (ARG located in the chromosome = ARGs-Chr, ARG located in the chromosome and integrated within an IS = ISs-ARGs, ARG located in the plasmid and integrated within an IS = ISs-Plasmid-ARGs and ARGs present on plasmids but not within an insertion sequence = Plasmid-ARGs). The proportion of isolates carrying the AMR genomic trait at each location is given in **Table S5**. If more than 50% of the isolates contain the AMR genomic trait in a specific location, this location is considered predominant, otherwise it is labelled as no preference.

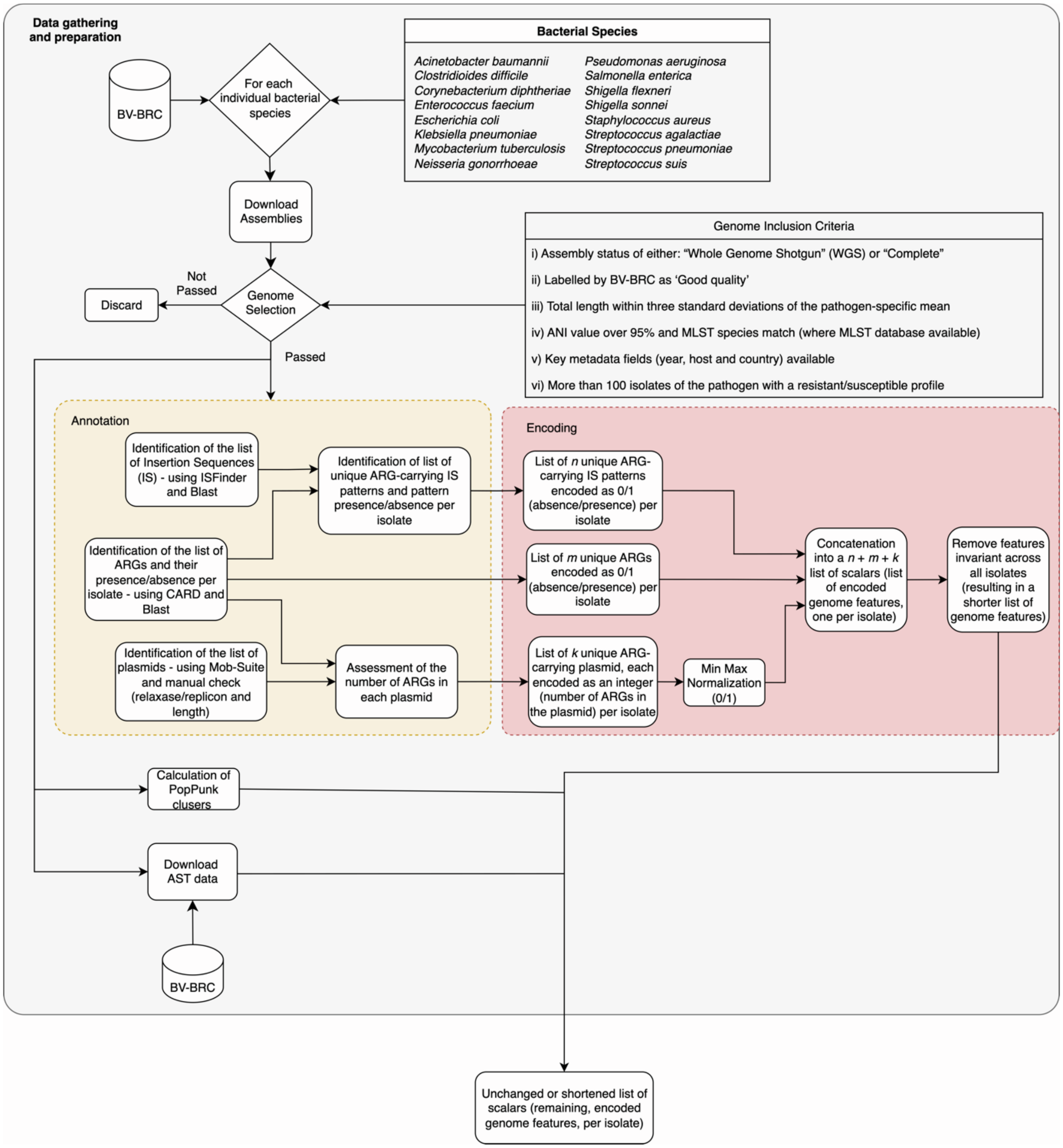

Figure S20. Data analysis and processing part 1: Data gathering and preparation, related to Figure 2. Activities are grouped into modules, represented as panels of different colours; the arrows represent the data flow.

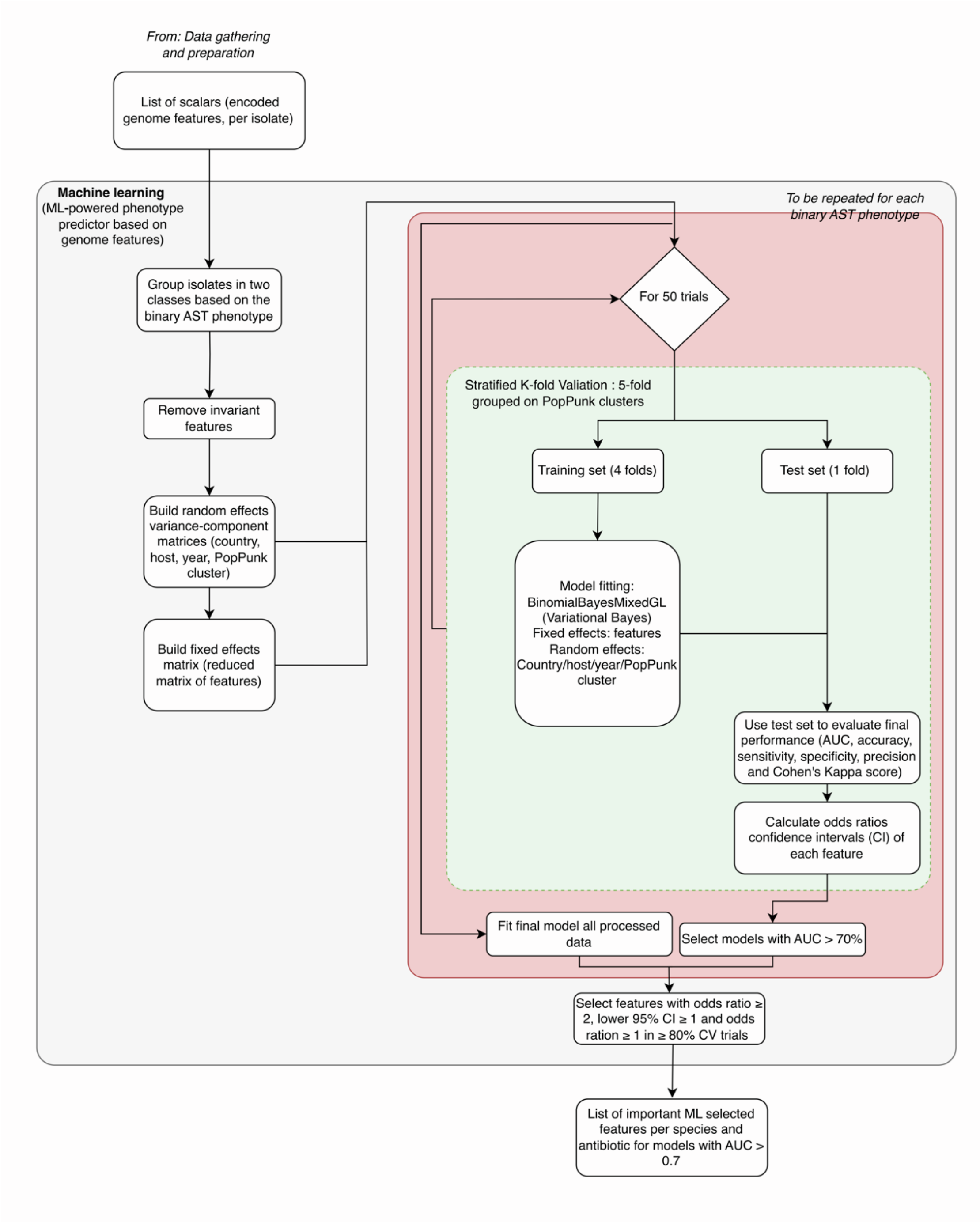

Figure S21. Data analysis and processing part 2: Machine learning, related to Figure 2. Activities are grouped into modules, represented as panels of different colours; the arrows represent the data flow.

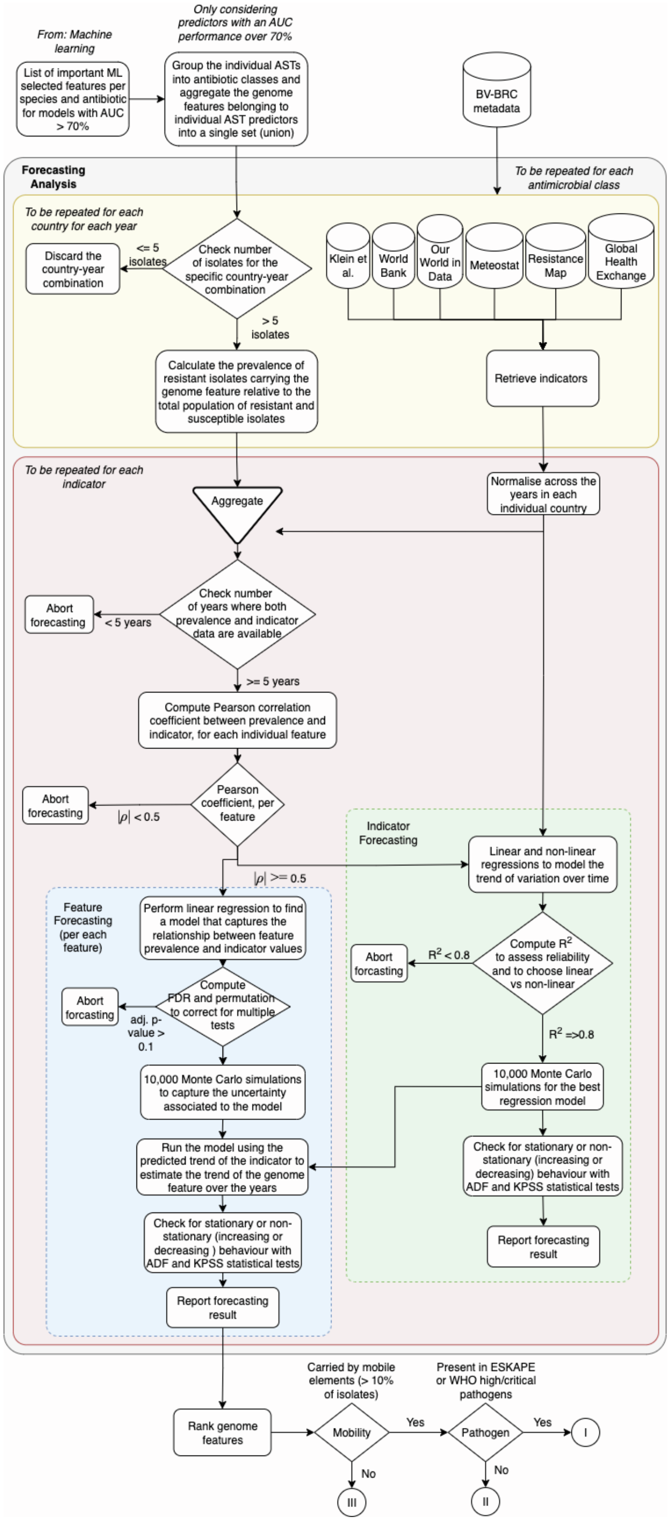

Figure S22. Data analysis and processing part 3: Forecasting analysis, related to Figure 2. Activities are grouped into modules, represented as panels of different colours; the arrows represent the data flow.

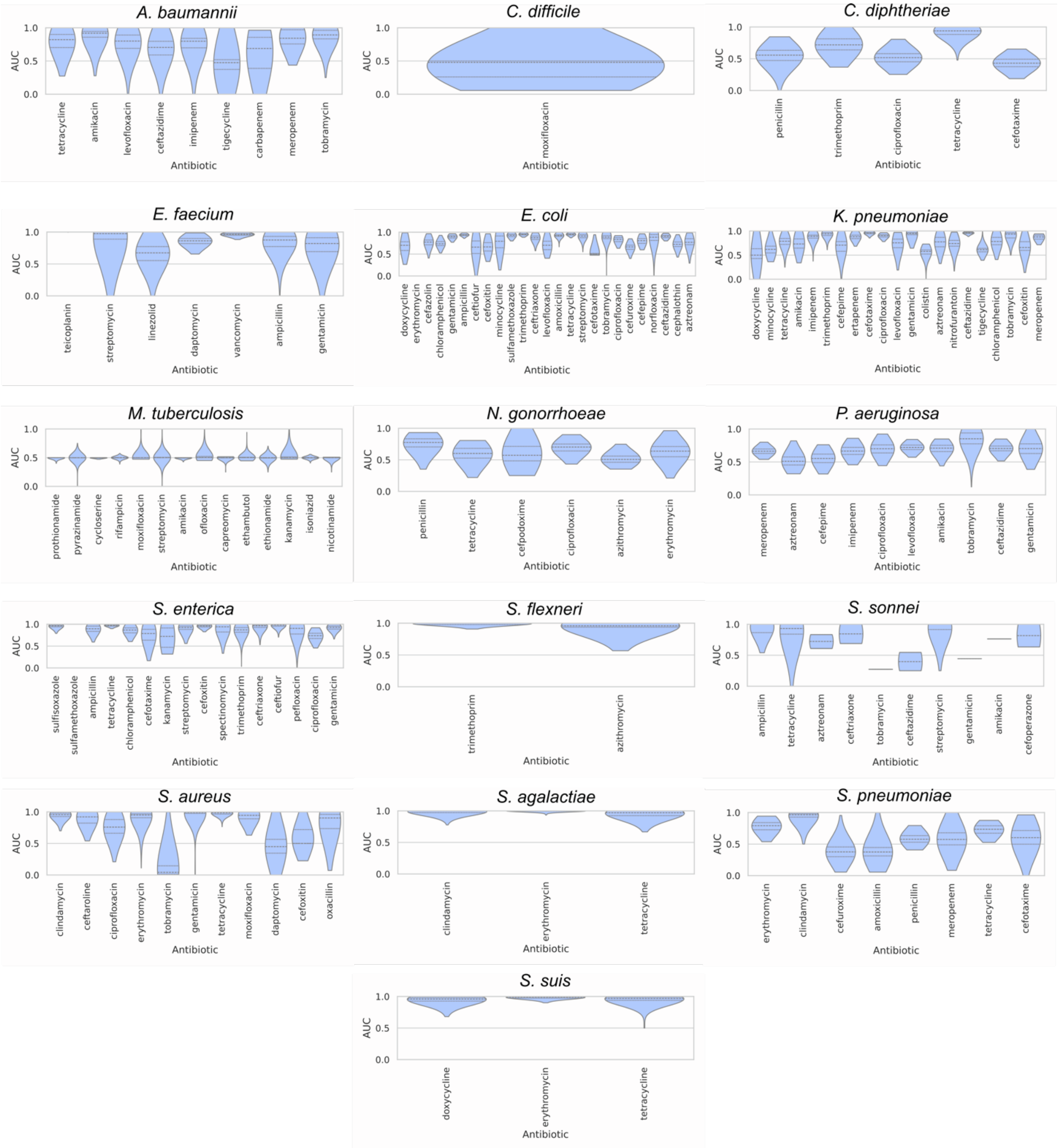

Figure S23. **Machine learning performance AUC by species and antibiotic model, related to STAR Methods.** The supervised ML pipeline predicts the resistance/susceptibility profiles of all 16 bacterial species. ML performance results are given for the area under the curve AUC, calculated from 50 training runs for each antimicrobial model.

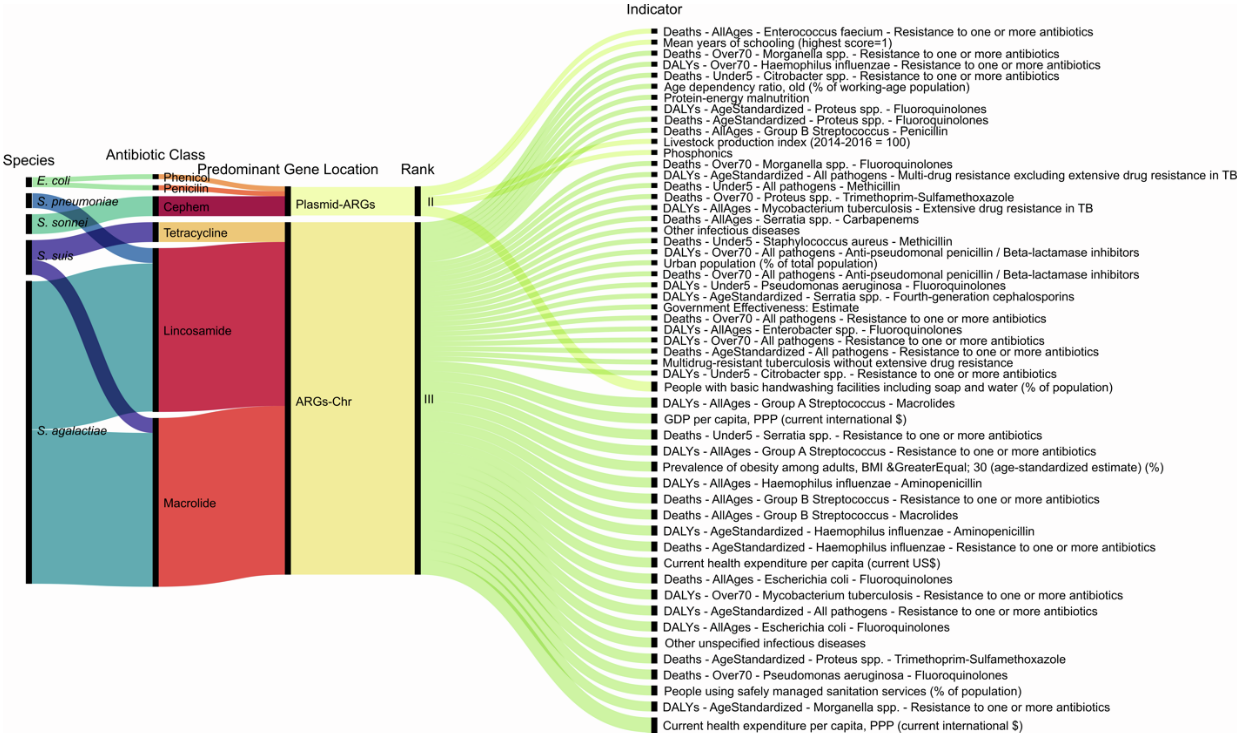

Figure S24. Sankey flow diagram illustrating forecast associations involving the ARG *ermB,* related to STAR Methods. The diagram depicts relationships between *ermB*, the bacterial species in which it was selected, the associated antibiotic classes identified by the machine learning models, the predominant gene location, feature rank (I/II), and the corresponding indicators. Only associations where *ermB* was selected by the ML models and linked to increasing or stationary indicators are shown. Line thickness reflects the number of distinct indicators connected to each node.

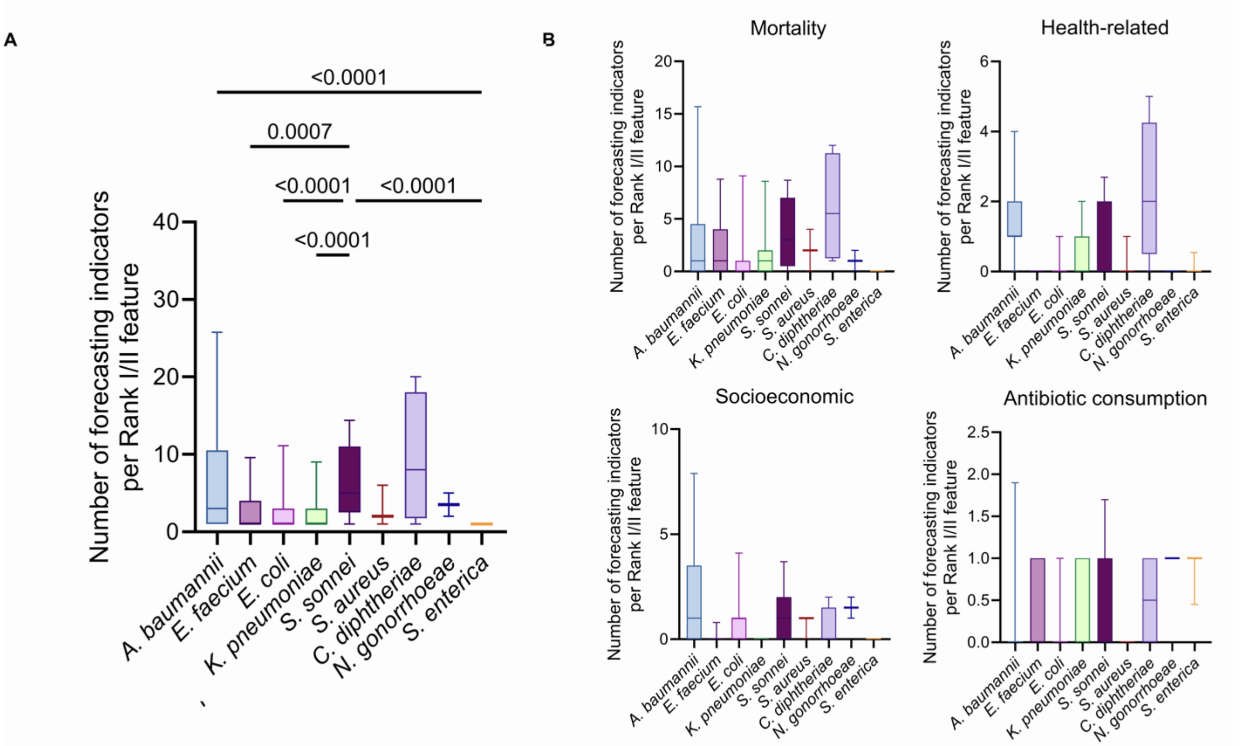

Figure S25. **Number of increasing or stationary forecasting indicators per rank I/II genomic feature, related to Figure 5.** (A) overall number of forecasting indicators per feature including antibiotic consumption, health-related, socioeconomic, environmental and mortality indicators. Significant statistical differences shown as line with adjusted *p*-value above plot (Kruskal-Wallis, two-tailed). (B) number of forecasting indicators per feature shown separately for antibiotic consumption, health-related, socioeconomic and mortality indicators. In each plot the box shows the interquartile range of the data with the median denoted by a horizontal line, and the whiskers indicate the range.

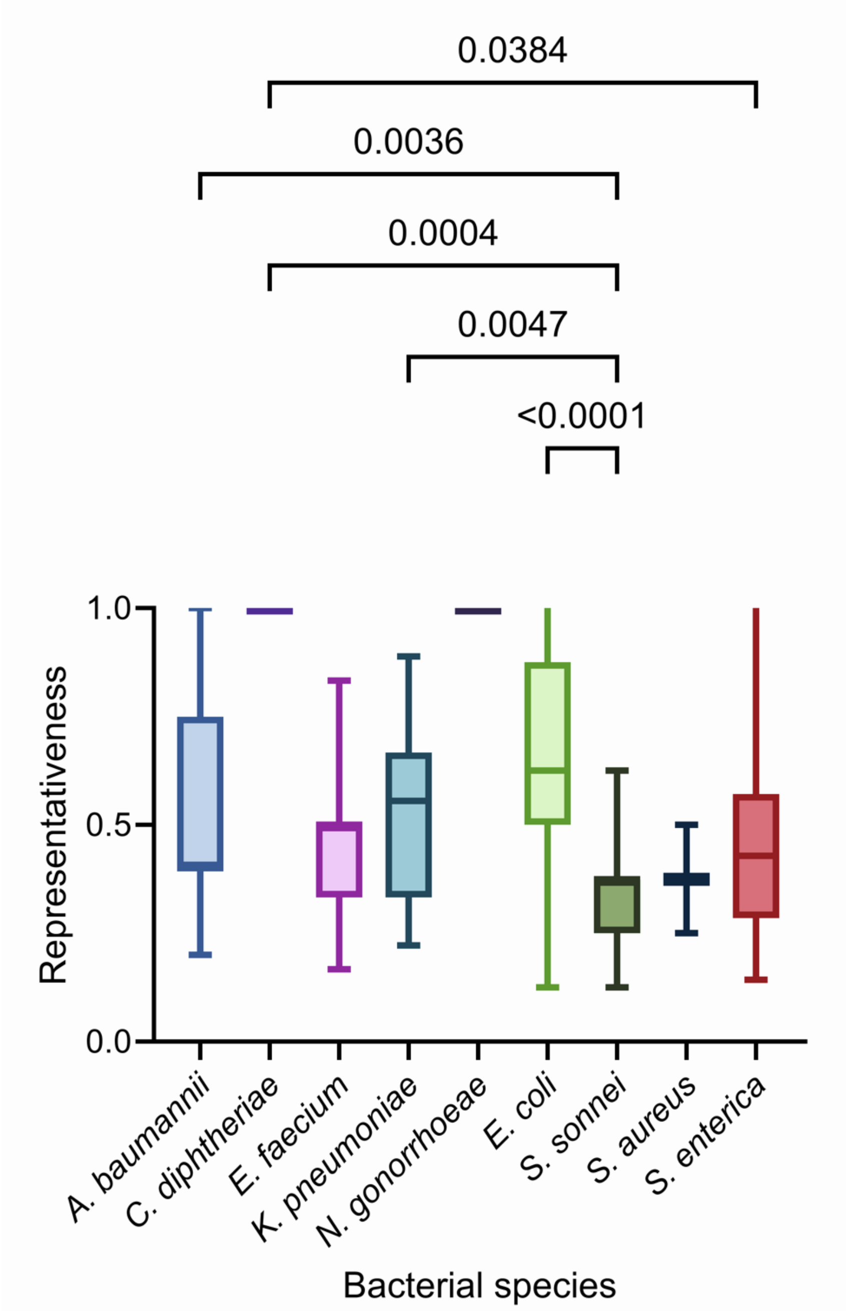

Figure S26. **Boxplot of the representativeness of the selected rank I/II features across each pathogen relative to the ML models, related to Figure 3.** For each pathogen we calculated a representativeness coefficient for the selected rank I/II features, defined as the number of resistance classes (in aggregated ML models with an AUC > 0.7) in which the feature was selected, relative to the number in which it was present in the input. For each pathogen, the box shows the interquartile range of the data with the median denoted by a horizonal line, and the whiskers indicate the range. Significantly different distributions are indicated on the plot with horizontal brackets and the adjusted *p*-value given (Kruskal-Wallis, two-tailed, Dunn's adj. *p*-values).

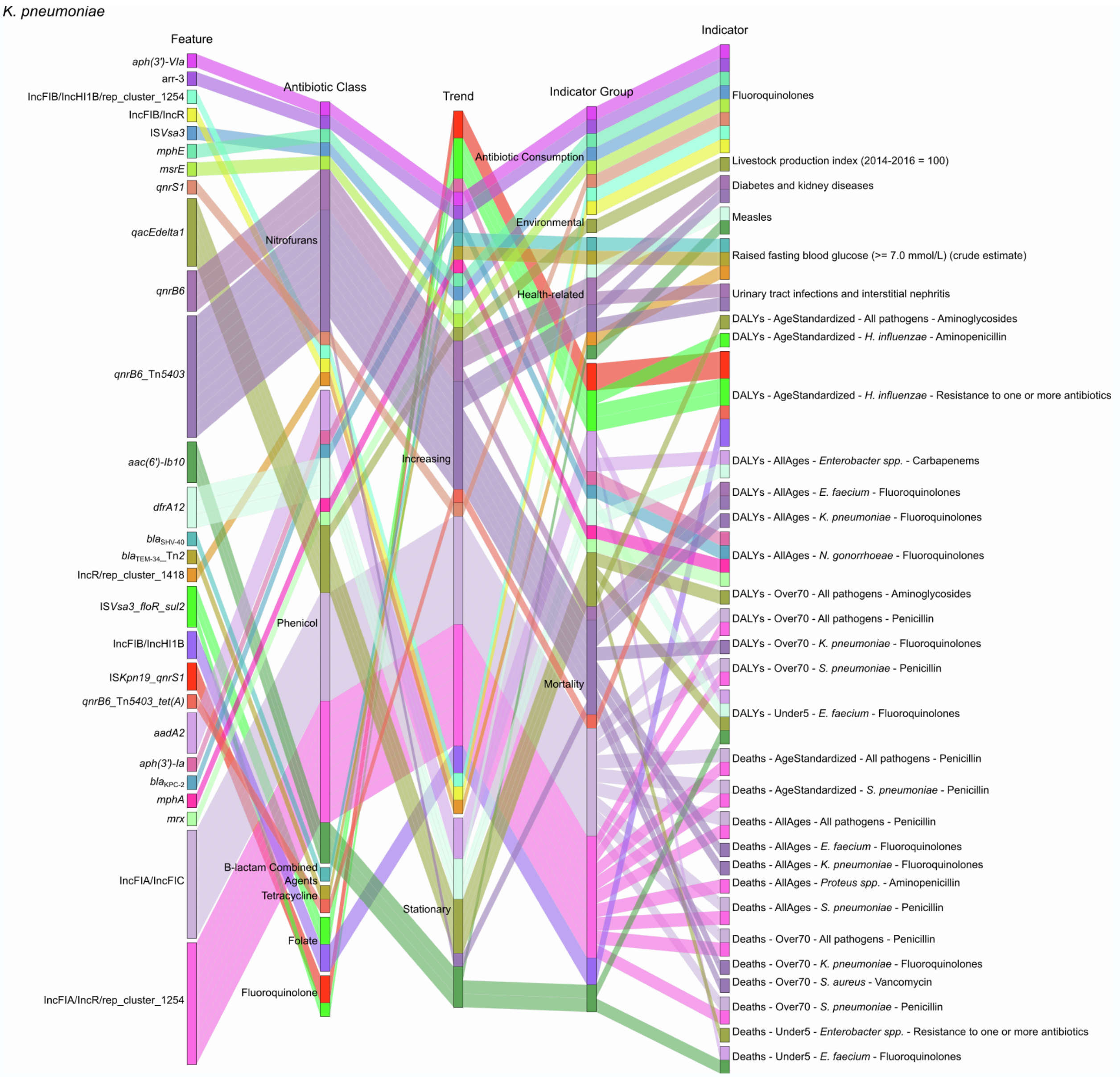

Figure S27. *K. pneumoniae* Sankey flow diagram, related to Figure 5. This Sankey diagram illustrates the relationships between AMR genomic features (Feature), their associated antibiotic class identified via ML (Antibiotic Class), the projected trend of each indicator (whether the indicator was forecast to increase or remain stationary, Trend), indicator groups (Indicator Group), and specific indicators (Indicator) within each group. Only rank I/II features associated with increasing or stationary indicators are displayed. The thickness of the links represents the number of different indicators, while the colours correspond to the associated genomic feature.

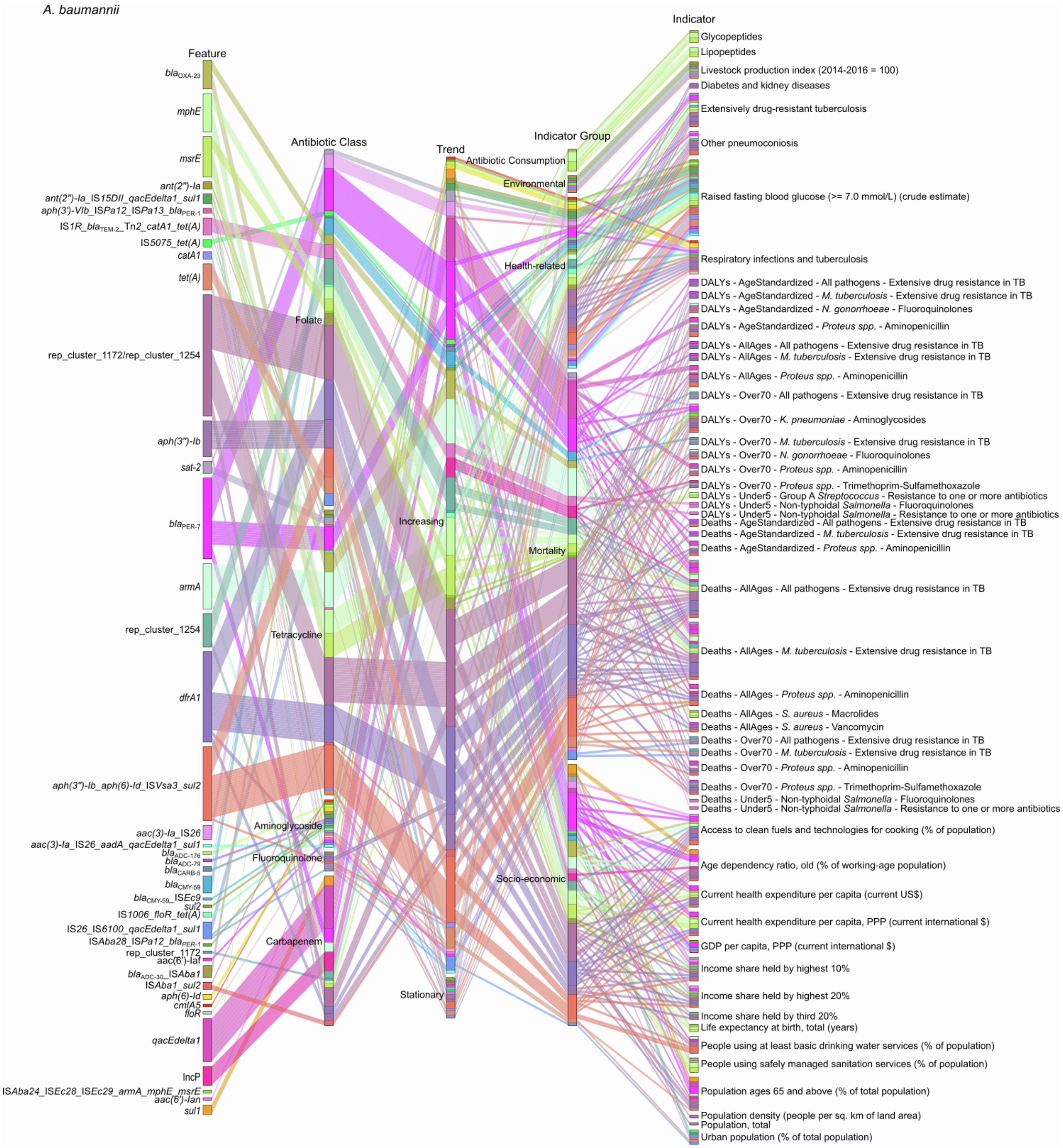

Figure S28. ***A. baumannii Sankey*** **flow diagram, related to Figure 5.** This Sankey diagram illustrates the relationships between AMR genomic features (Feature), their associated antibiotic class identified via ML to each feature (Antibiotic Class), the projected trend of each indicator (whether the indicator was forecast to increase or remain stationary, Trend), indicator groups (Indicator Group), and specific indicators (Indicator) within each group. Only rank I/II features associated with increasing or stationary indicators are displayed. The thickness of the links represents the number of different indicators, while the colours correspond to the associated genomic feature.

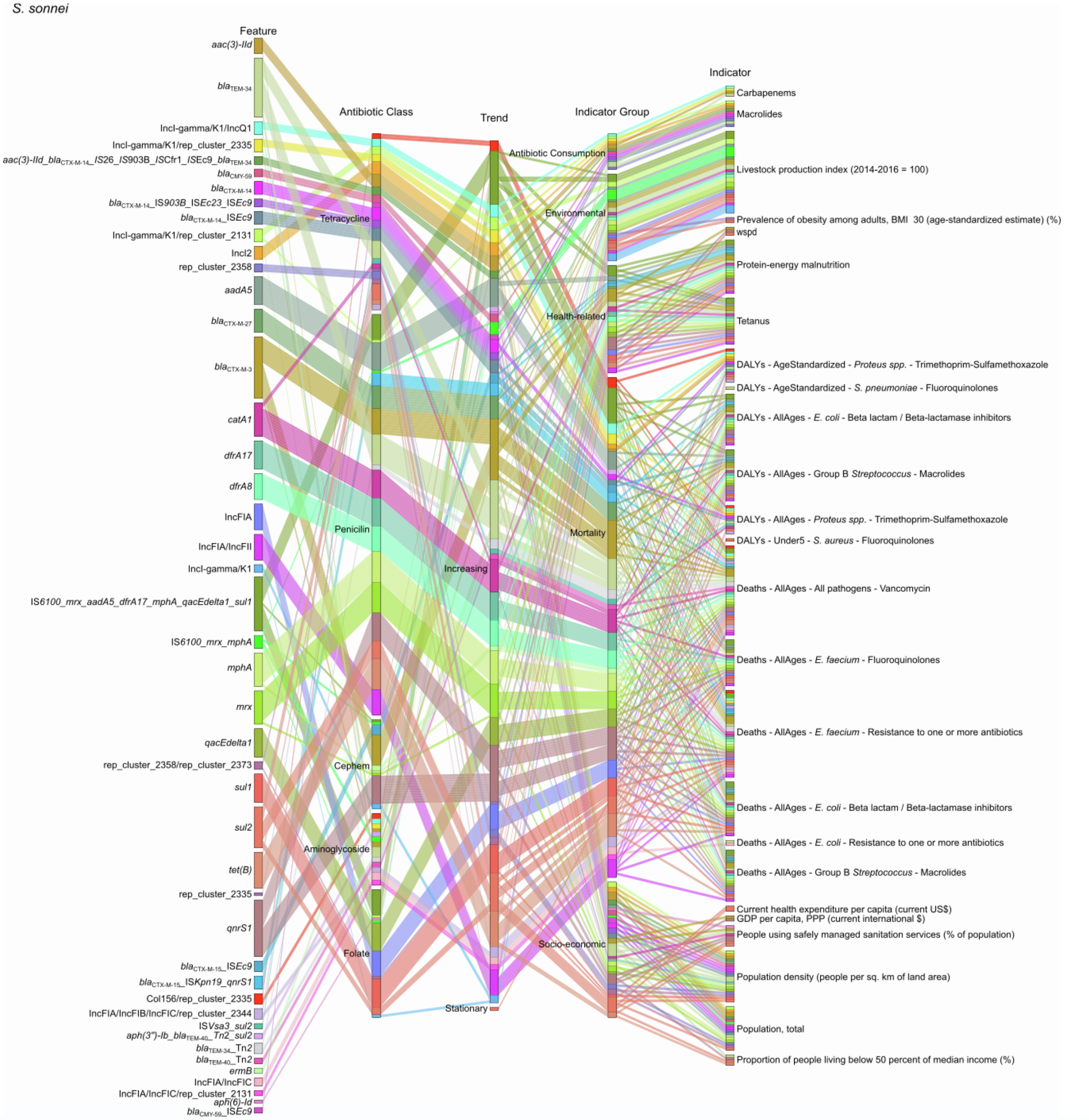

Figure S29. *S. sonnei* Sankey flow diagram related to Figure 5. This Sankey diagram illustrates the relationships between AMR genomic features (Feature), their associated antibiotic class identified via ML to each feature (Antibiotic Class), the projected trend of each indicator (whether the indicator was forecast to increase or remain stationary, Trend), indicator groups (Indicator Group), and specific indicators (Indicator) within each group. Only rank I/II features associated with increasing or stationary indicators are displayed. The thickness of the links represents the number of different indicators, while the colours correspond to the associated genomic feature.

E. coli

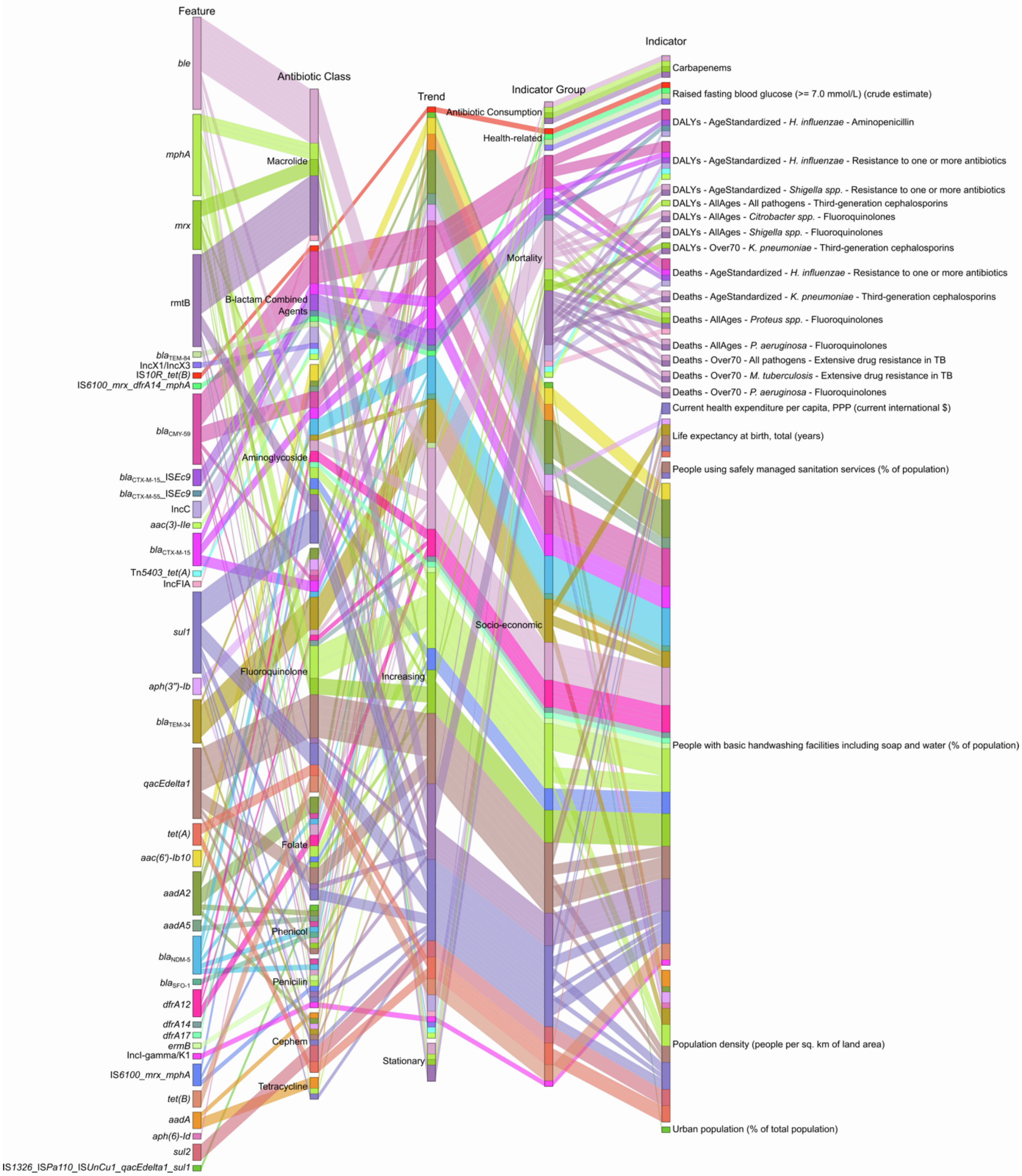

Figure S30. ***E. coli* Sankey flow diagram, related to Figure 5.** This Sankey diagram illustrates the relationships between AMR genomic features (Feature), their associated antibiotic class identified via ML to each feature (Antibiotic Class), the projected trend of each indicator (whether the indicator was forecast to increase or remain stationary, Trend), indicator groups (Indicator Group), and specific indicators (Indicator) within each group. Only rank I/II features associated with increasing or stationary indicators are displayed. The thickness of the links represents the number of different indicators, while the colours correspond to the associated genomic feature.

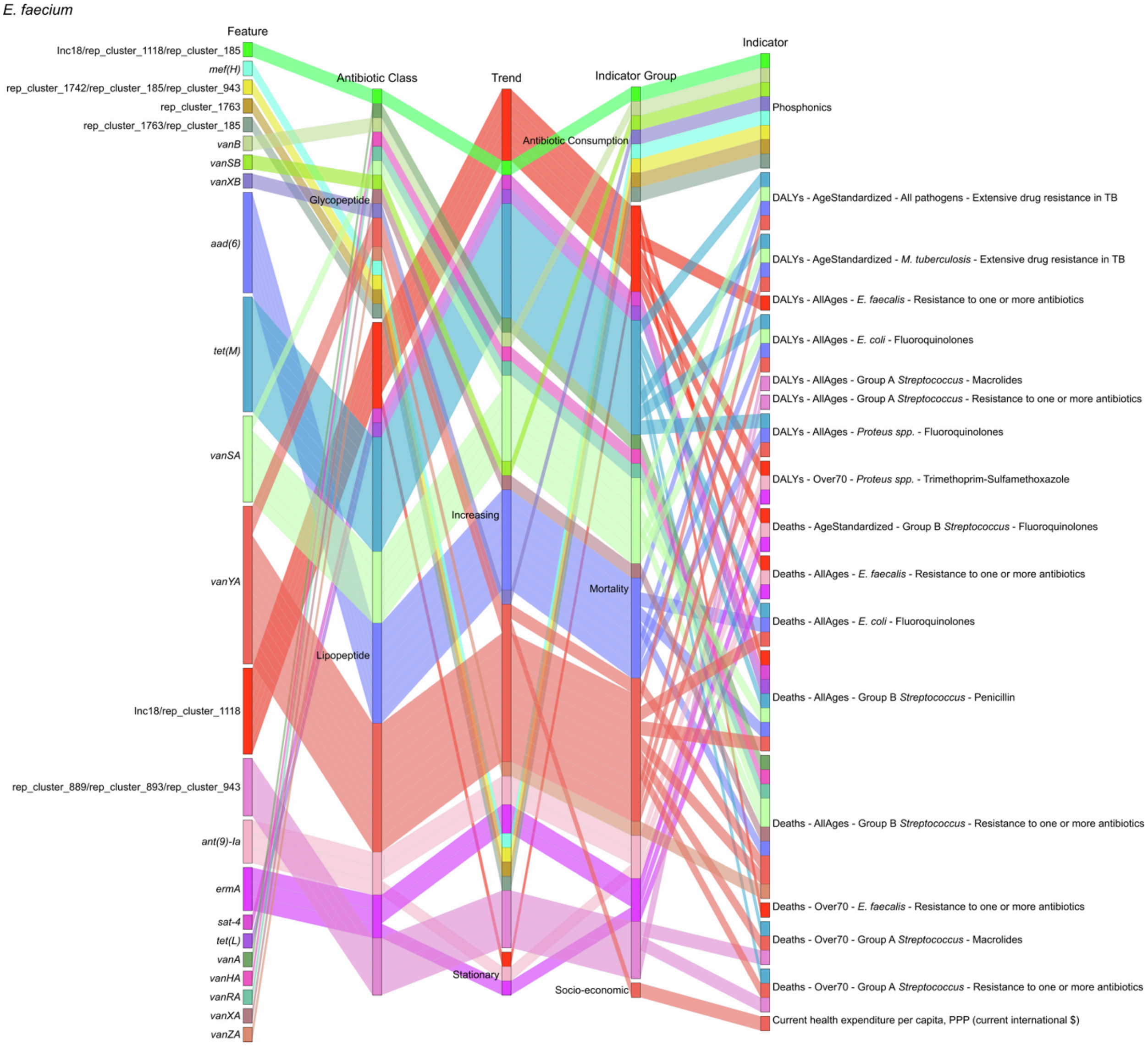

Figure S31. *E. faecium* Sankey flow diagram, related to Figure 5. This Sankey diagram illustrates the relationships between AMR genomic features (Feature), their associated antibiotic class identified via ML to each feature (Antibiotic Class), the projected trend of each indicator (whether the indicator was forecast to increase or remain stationary, Trend), indicator groups (Indicator Group), and specific indicators (Indicator) within each group. Only rank I/II features associated with increasing or stationary indicators are displayed. The thickness of the links represents the number of different indicators, while the colours correspond to the associated genomic feature.

S. enterica

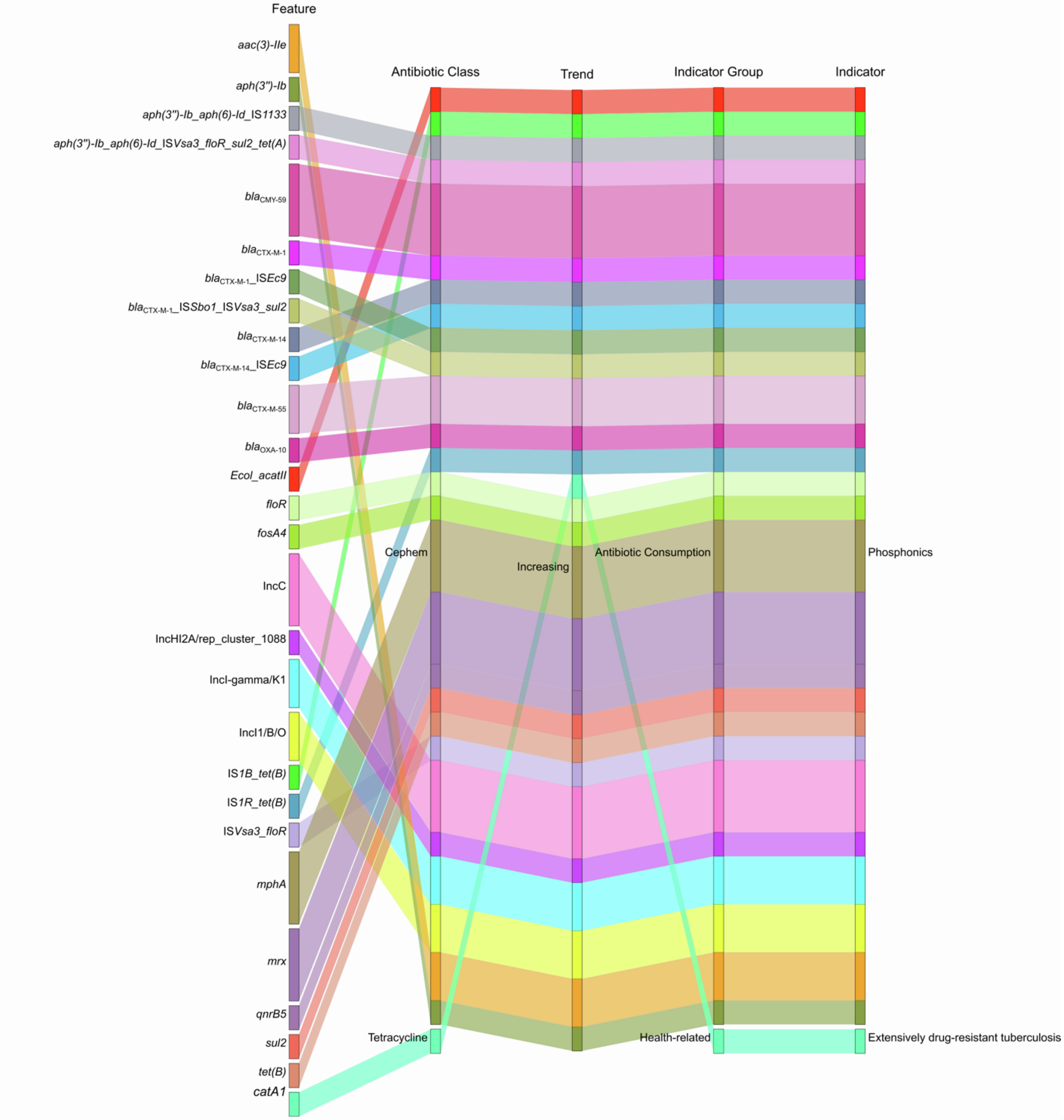

Figure S32. *S. enterica* Sankey flow diagram, related to Figure 5. This Sankey diagram illustrates the relationships between AMR genomic features (Feature), their associated antibiotic class identified via ML to each feature (Antibiotic Class), the projected trend of each indicator (whether the indicator was forecast to increase or remain stationary, Trend), indicator groups (Indicator Group), and specific indicators (Indicator) within each group. Only rank I/II features associated with increasing or stationary indicators are displayed. The thickness of the links represents the number of different indicators, while the colours correspond to the associated genomic feature.

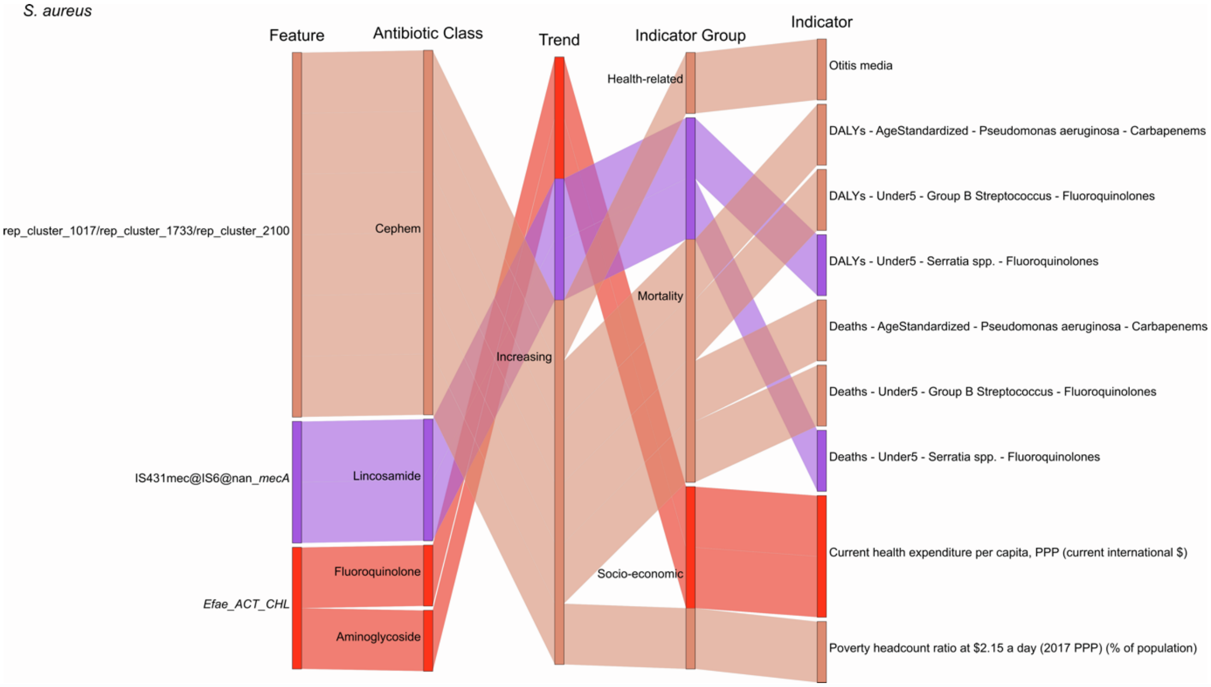

Figure S33. *S. aureus* Sankey flow diagram, related to Figure 5. This Sankey diagram illustrates the relationships between AMR genomic features (Feature), their associated antibiotic class identified via ML to each feature (Antibiotic Class), the projected trend of each indicator (whether the indicator was forecast to increase or remain stationary, Trend), indicator groups (Indicator Group), and specific indicators (Indicator) within each group. Only rank I/II features associated with increasing or stationary indicators are displayed. The thickness of the links represents the number of different indicators, while the colours correspond to the associated genomic feature.

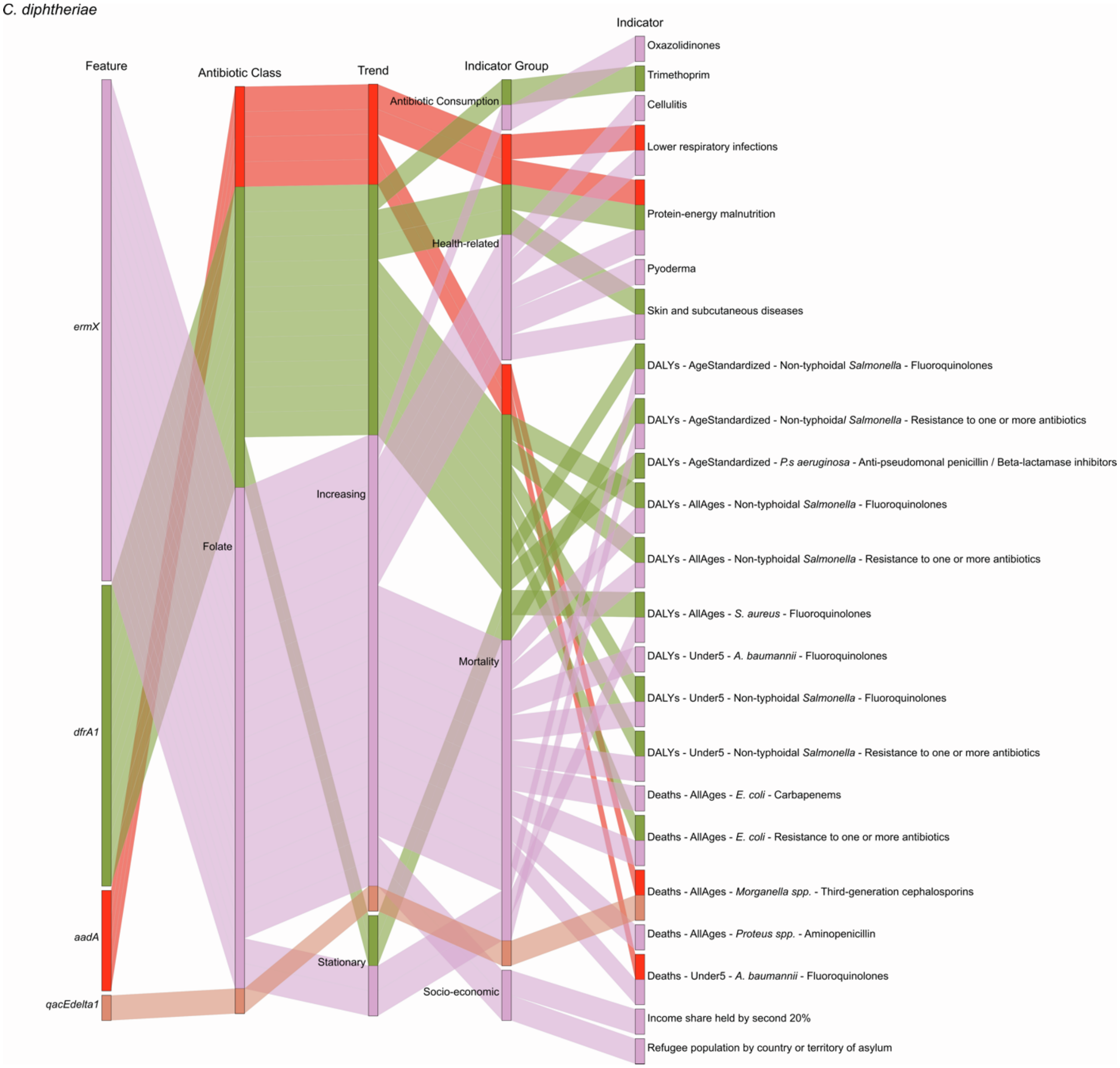

Figure S34. ***C. diphtheriae*** **Sankey flow diagram, related to Figure 5.** This Sankey diagram illustrates the relationships between AMR genomic features (Feature), their associated antibiotic class identified via ML to each feature (Antibiotic Class), the projected trend of each indicator (whether the indicator was forecast to increase or remain stationary, Trend), indicator groups (Indicator Group), and specific indicators (Indicator) within each group. Only rank I/II features associated with increasing or stationary indicators are displayed. The thickness of the links represents the number of different indicators, while the colours correspond to the associated genomic feature.

Species and infection site (infection site for prevalence only)	Spearman correlation (data aggregated by year and country)	Spearman correlation (data aggregated by year and income group)
E. coli BSI	-0.09	-0.19
E. coli UTI	0.16	0.34
A. baumannii BSI	-0.06	0.00
K. pneumoniae BSI	-0.01	-0.14
K. pneumoniae UTI	-0.04	0.07
S. enterica BSI	0.02	-0.02
S. enterica GI	0.00	0.26
S. aureus BSI	0.03	0.18
N. gonorrhoeae UG	0.10	0.15

Table S4. **Spearman correlation coefficients between GLASS body-site-specific pathogen prevalence rates (incidence per million population) and counts of the number of sequence isolates in the study, related to STAR Methods**. Data are aggregated by country and year in column 2 and World Bank income group (FY26 classification)[3] and year in column 3.

Species	Reference Name	GenBank Accession	Use
A. baumannii	*A. baumannii* ATCC 19606	CP045110.1	ANI
C. difficile	*C. difficile* S-0253	CP076401.1	ANI
C .diphtheriae	*C. diphtheriae* NCTC11397	LN831026.1	ANI
E. faecium	*E. faecium* SRR24	CP038996.1	ANI
E. coli	*E. coli* str. K-12 substr. MG1655	U00096.3	ANI
K. pneumoniae	*K. pneumoniae* subsp. pneumoniae HS11286	CP003200.1	ANI
M. tuberculosis	*M. tuberculosis* H37Rv	AL123456.3	ANI
N. gonorrhoeae	*N. gonorrhoeae* TUM19854	AP023069.1	ANI
P. aeruginosa	*P. aeruginosa* PAO1	AE004091.2	ANI
S. enterica	*S. enterica* subsp. enterica serovar Typhimurium str. LT2	AE006468.2	ANI
S. flexneri	*S. flexneri* 2a str. 301	AE005674.2	ANI
S. sonnei	*S. sonnei* strain ATCC 29930	CP026802.1	ANI
S. aureus	*S. aureus* subsp. aureus NCTC 8325	CP000253.1	ANI
S. agalactiae	*S. agalactiae* strain NGBS128	CP012480.1	ANI
S. pneumoniae	*S. pneumoniae* assembly NCTC7465	LN831051.1	ANI
S. suis	*S. suis* BM407	FM252032.1	ANI
S. flexneri	*S. flexneri* 2a assembly ASM2962549v1	GCF_029625495.1	KmerID
S. flexneri	*S. flexneri* assembly ASM2235464v1	GCF_022354645.1	KmerID
S. flexneri	*S. flexneri* assembly ASM295033v1	GCF_002950335.1	KmerID
S. flexneri	*S. flexneri* G1663 assembly ASM102185v1	GCF_001021855.1	KmerID
S. flexneri	*S. flexneri* 1b assembly ASM3050607v1	GCF_030506075.1	KmerID
S. flexneri	*S. flexneri* 1a assembly ASM157812v1	GCF_001578125.1	KmerID
S. flexneri	*S. flexneri* assembly ASM2473232v1	GCF_024732325.1	KmerID
S. flexneri	*S. flexneri* assembly ASM2235396v1	GCF_022353965.1	KmerID
S. flexneri	*S. flexneri* 1bassembly ASM3050605v1	GCF_030506055.1	KmerID
S. flexneri	*S. flexneri* 2a str 2457T	NC_004741.1	KmerID
S. flexneri	*S. flexneri* 2002017	GCA_000022245.1	KmerID
S. sonnei	*S. sonnei* assembly ASM972999v1	GCF_009729995.1	KmerID
S. sonnei	*S. sonnei* assembly ASM360642v1	GCF_003606425.1	KmerID
S. sonnei	*S. sonnei* assembly ASM3829866v1	GCF_038298665.1	KmerID

S. sonnei	*S. sonnei* assembly ASM3704248v1	GCF_037042485.2	KmerID
S. sonnei	*S. sonnei* assembly ASM966451v1	GCF_009664515.1	KmerID
S. sonnei	*S. sonnei* assembly ASM3836717v1	GCF_038367175.1	KmerID
S. sonnei	*S. sonnei* assembly ASM3836735v1	GCF_038367355.1	KmerID
S. sonnei	*S. sonnei* assembly ASM244253v1	GCF_002442535.1	KmerID
S. sonnei	*S. sonnei* assembly ASM2590845v1	GCF_025908455.1	KmerID
S. sonnei	*S. sonnei* Ss046	NC_007384.1	KmerID
S. sonnei	*S. sonnei* 53G	GCA_000283715.1	KmerID

Table S20. **Reference genomes and accessions used for ANI- and KmerID-based species validation of genomes included in the study, related to STAR Methods.**

Species	Total ARG-IS Combinations	Mean Isolates per ARG-IS	Standard Deviation	Range	Mean Isolates for Selected Features (rank I/II)
A. baumannii	187	8.7	36.2	1-455	25.3
C. difficile	2	1.0	0.0	-	-
C. diphtheriae	11	5.6	10.0	1-35	35.0
E. faecium	41	3.7	5.7	1-29	-
E. coli	584	6.1	26.2	1-384	77.2
K. pneumoniae	769	10.4	56.0	1-1006	95.0
M. tuberculosis	2	3.0	2.8	1-5	-
N. gonorrhoeae	0	-	-	-	-
P. aeruginosa	67	2.3	6.0	1-48	-
S. enterica	256	6.4	14.0	1-116	21.9
S. flexneri	45	4.4	11.4	1-77	-
S. sonnei	106	8.1	29.5	1-208	49.2
S. aureus	88	17.1	95.3	1-880	880.0
S. agalactiae	1	1.0	-	-	-
S. suis	23	6.0	15.0	1-72	-

Table S21. **Number and descriptive statistics (mean, standard deviation and range) of ARG-carrying IS combinations in the machine learning inputs and the mean number of isolates per ARG-carrying IS combination, related to STAR Methods.**

Supplemental Data 1: Forecasting analysis of *M. tuberculosis*, *N. gonorrhoeae*, and *C. difficile*

The machine learning pipeline was unable to robustly correlate AMR phenotypes with AMR genomic features in *M. tuberculosis*, *N. gonorrhoeae*, and *C. difficile*. Despite this, given the public-health importance of these pathogens, forecasting analysis was conducted, considering all AMR genomic features found in these pathogens, using the same pipeline as shown in **Figure S22**. Only a small number of features were forecast to increase in prevalence in association with increasing/stationary indicators in each species (**Table S9**), seven in *M. tuberculosis* (*aac(2')-Ic*, *bla$_C$*, *efpA*, *erm(37), mfpA*, *rv1877*, *rv2856*), all associated with cycloserine resistance; two in *N. gonorrhoeae* (IncP, bla$_{TEM-135}$) associated with fluoroquinolone and penicillin resistance; and four in *C. difficile* (*cfrE*, *aph(3')-IIIa, ermB, sat-4*) associated with fluoroquinolone resistance.
The genomic feature *ermB* in *C. difficile* was associated with 144 indicators of which 91.6% were mortality or health-related, reflecting the importance of *C. difficile* as a nosocomial infection[4]. In *M. tuberculosis*, 18 indicators were associated with each of the seven genomic features. Of these indicators 11 were mortality indicators, three health-related and four were socioeconomic. Notably, two of the health-related indicators were related to diabetes risk factors (raised blood glucose and obesity levels), suggesting that the well-established comorbid relationship between *M. tuberculosis* and diabetes[5] is associated with increasing resistance in this species. Increasing prevalence of *N. gonorrhoeae* was associated with three mortality indicators, two socioeconomic indicators and antibiotic consumption of lipopeptides. Notably both socioeconomic indicators were related to increasing elderly populations ("population ages 65 and above" and "age dependency ratio"), potentially reflecting a growing concern of increasing rates of *N. gonorrhoeae* infections among older individuals[6] and an increased likelihood of resistant strains being isolated in older individuals[7].

Supplemental Methods 1
When using mixed indicator sources, definitional heterogeneity is an inherent limitation of global-scale analyses and cannot be completely removed. In this analysis, we sought to minimise this by restricting our data sources to repositories that apply standardised collection, curation, and quality-control procedures, collate data at a global level, and provide a clear definition and source for each indicator used (Table S2). Each data provider used in this study employs extensive data harmonisation and validation.
Importantly, indicators were analysed independently within the forecasting framework and were not aggregated into composite indices spanning heterogeneous repositories. Each indicator entered the model as a separate temporal trajectory.
All indicators were scaled between 0 and 1 prior to modelling to standardise numerical ranges and avoid artefacts arising from differences in measurement units. This scaling harmonises magnitude but does not alter construct definitions. Associations are therefore interpreted directionally (increasing, decreasing or stationary) rather than as absolute cross-domain effect size comparisons.
Countries with insufficient temporal overlap between genomic data and indicator time series were excluded to ensure adequate longitudinal alignment.

Specifically:
- World Bank: standardises data using harmonised accounting frameworks, alignment to national standards, statistical modelling of missing values, and cross validation procedures. Although data gaps and definitional heterogeneity cannot be fully resolved, the World Bank applies harmonised accounting frameworks and metadata standards that support cross-country trend analysis, which aligns with the trend-based forecasting objective of this study (https://datatopics.worldbank.org/world-development-indicators/sources-and-methods.html)
- ResistanceMap" antibiotic consumption data are derived from IQVIA MIDAS sales data curated from national sample surveys and converted into defined daily doses using WHO methodology (https://resistancemap.onehealthtrust.org/MethodologyAU.php).
-OurWorldinData: applies a robust and scalable data-cleaning, unit-harmonisation, age-standardisation and quality-control pipelines (https://github.com/owid/etl).
- GHDx: provides extensive metadata, including coverage, and definitions, standardised country coding, age-standardised estimates, harmonised units, and multi-layer validation of completeness and plausibility (https://www.healthdata.org/data-tools-practices/data-collection).

Robustness to Coverage and Source Heterogeneity

To assess whether the completeness across source types differed and mixing repositories disproportionately influenced the results, we examined the distribution of retained high-priority feature–indicator associations across indicator source categories (World Bank, ResistanceMap, Our World in Data, GHDx). Across repositories there were no statistical differences in the completeness of non-mortality indicators, only differences between mortality (GHDx) and non-mortality indicators (Kruskal-Wallis test: $H = 926.4$, $p = 5.14 \times 10^{-198}$; Dunn's post-hoc test with Bonferroni correction: GHDx vs Klein *et al*., Meteostat, Our World in Data, ResistanceMap and World Bank all adjusted *p*-values ≈ 0; Klein *et al.* vs Meteostat, Our World in Data, ResistanceMap and World Bank all adjusted *p*-values ≈ 1; Meteostat vs Our World in Data and ResistanceMap adjusted *p*-values ≈ 1; Meteostat vs World Bank adjusted *p*-value = 0.9573; Our World in Data vs ResistanceMap and World Bank all adjusted *p*-values ≈ 1; ResistanceMap vs World Bank adjusted *p*-value = 0.3985). Additionally, we assessed the forecastable associations relative to the number of input indicators across repositories, for those with greater than 10 indicators (World Bank, Our World in Data, GHDx) using two-tailed pairwise proportion *Z-tests* (*GHDx vs ResistanceMap: Z = 0.511, p-*value=0.610; *GHDx vs WorldBank Data: Z = 0.007, p*-value=0.995; ResistanceMap vs WorldBank: Z=0.443, *p*-value=0.656), indicating that no single repository accounted for a disproportionate share of forecastable associations, and that the findings are not driven by any single data provider. Meteostat (n=6), Klein *et al*. (n=2) and Our World in Data (n=6) were excluded from the proportion Z-test due to their smaller number of indicators (n < 10), which precluded reliable statistical testing.

Despite these mitigation strategies, residual heterogeneity across countries and reporting systems remains unavoidable in global comparative analyses and should be considered when interpreting cross-country indicator associations.

Supplemental References

1. BV-BRC (2024). The Bacterial and Viral Bioinformatics Resource Center (BV-BRC). https://www.bv-brc.org/ Last accessed 01-09-24.
2. Natural Earth (2024). Natural Earth Data. https://www.naturalearthdata.com/ Last accessed 01-09-24.
3. World Bank Group (2025). World Bank country and lending groups FY26 (Last accessed 01-01-2026). https://datahelpdesk.worldbank.org/knowledgebase/articles/906519-world-bank-country-and-lending-groups.
4. Akorful, R.A.A., Odoom, A., Awere-Duodu, A., and Donkor, E.S. (2025). The Global Burden of Clostridioides difficile Infections, 2016-2024: A Systematic Review and Meta-Analysis. Infect Dis Rep *17*. https://doi.org/10.3390/idr17020031.
5. Boadu, A.A., Yeboah-Manu, M., Osei-Wusu, S., and Yeboah-Manu, D. (2024). Tuberculosis and diabetes mellitus: The complexity of the comorbid interactions. International Journal of Infectious Diseases *146*, 107140. https://doi.org/10.1016/j.ijid.2024.107140.
6. Kumar, B., Kaushal, I., Narayanan, B., and Narang, T. (2025). Sexually transmitted infections in the elderly: A growing concern in geriatric care. Indian J Sex Transm Dis AIDS *46*, 16-21. https://doi.org/10.4103/ijstd.ijstd_108_24.
7. Sarenje, K.L., Ngalamika, O., Maimbolwa, M.C., Siame, A., Munsaka, S.M., and Kwenda, G. (2022). Antimicrobial resistance of Neisseria gonorrhoeae isolated from patients attending sexually transmitted infection clinics in Urban Hospitals, Lusaka, Zambia. BMC Infect Dis *22*, 688. https://doi.org/10.1186/s12879-022-07674-y.
